# Integrative analysis identifies PIGK as an oncogenic glycosylphosphatidylinositol transamidase subunit with prognostic, immunological, and therapeutic relevance in head and neck cancer

**DOI:** 10.7150/ijms.121484

**Published:** 2026-01-01

**Authors:** Yi-Fang Yang, Jia-Bin Liao, Pei-Lun Yu, Chih-Yu Chou, Yu-Hsuan Lin

**Affiliations:** 1 Department of Medical Education and Research, Kaohsiung Veterans General Hospital, Kaohsiung 813, Taiwan.; 2 Department of Pathology and Laboratory Medicine, Kaohsiung Veterans General Hospital, Kaohsiung 813, Taiwan.; 3 Department of Otolaryngology, Head and Neck Surgery, Kaohsiung Veterans General Hospital, Kaohsiung 813, Taiwan.; 4 School of Medicine, National Yang Ming Chiao Tung University, Taipei 112, Taiwan.; 5 School of Medicine, Chung Shan Medical University, Taichung 402, Taiwan.; 6 School of Medicine, College of Medicine, National Sun Yat-sen University, Kaohsiung 804, Taiwan.

**Keywords:** PIGK, head and neck cancer, FAM20C, tumor microenvironment, taxane sensitivity

## Abstract

Background: Glycosylphosphatidylinositol transamidase (GPI-T) catalyzes the attachment of glycosylphosphatidylinositol (GPI) anchors to membrane proteins implicated in oncogenic signaling. However, the specific contribution of individual GPI-T subunits to head and neck cancer (HNC) remains unclear.

Methods: We first compare the expression profiles of GPI-T subunits in HNC and then integrate multi-omics analyses to assess *phosphatidylinositol glycan class K* (*PIGK*) expression, genomic alterations, function and pathway enrichment, molecular interactions, and immune associations. Clinical relevance is validated by immunohistochemistry on tissue microarray, and *in vitro* assays were conducted to assess PIGK-mediated phenotypes, regulation of *Family with sequence similarity 20-member C* (*FAM20C*), taxane response, and cancer-associated fibroblast (CAF) formation.

Results: Among the five subunits, PIGK was uniquely and consistently upregulated at both mRNA and protein levels in tumors. High PIGK expression correlates with aggressive clinicopathological features and poor survival across independent cohorts. Genomic analysis shows that *PIGK* overexpression is associated with copy number gains and inversely correlated with mutations in *FAT1*, *CDKN2A*, *NOTCH1*, and *CASP8*. Functionally, PIGK knockdown significantly suppressed cell migration, invasion, proliferation, and colony formation, reduced *FAM20C* expression, decreased sensitivity to paclitaxel and docetaxel, and attenuated fibroblast activation. Enrichment analysis of co-expressed genes showed involvement in cancer-related biological processes, while protein-level interactors of PIGK were enriched in GPI-anchor biosynthesis and membrane-associated pathways. Clinically, patients with* PIGK*^high^/*FAM20C*^high^ profile exhibited the worst survival outcomes.

Conclusion: PIGK functions as a potential oncogenic driver in HNC with prognostic and therapeutic relevance. Its association with FAM20C, taxane response, and modulation of fibroblast activation provides insights into PIGK-mediated oncogenesis and may inform patient stratification strategies.

## Introduction

Head and neck cancer (HNC) poses a global health burden, with over 900,000 new cases and 450,000 deaths annually 1. Although treatment outcomes have improved for human papillomavirus (HPV)-related HNC, especially for those arising in the oropharynx, the prognosis for HPV-unrelated cases, which comprise most HNC cases, remains poor 2, 3. This is partly due to the absence of targetable oncogenic drivers, which are characterized by frequent mutations in tumor suppressor genes such as *TP53*, *CDKN2A*, *FAT1*, and *AJUBA* 4. Additionally, growing evidence indicates that the aggressiveness and treatment resistance of HNC are shaped by tumor-intrinsic alterations and immune and stromal components of the intricate and heterogeneous tumor microenvironment (TME) 2, 3. Therefore, the identification of biomarkers that reflect both malignant cell behavior and microenvironmental interactions, especially those with prognostic value, is urgently needed. Given this need, we turned to the glycosylphosphatidylinositol transamidase (GPI-T) complex, a key enzyme involved in glycosylphosphatidylinositol (GPI)-anchor biosynthesis 5-9. Dysregulation of GPI-anchored proteins (GPI-APs) has been implicated in several cancers 10, raising the possibility that their maturation machinery may contribute to HNC progression.

The GPI-T complex is a membrane-bound enzyme located in the endoplasmic reticulum (ER) that catalyzes the attachment of GPI anchors to proteins. It comprises five subunits:* phosphatidylinositol glycan class U* (*PIGU*),* glycosylphosphatidylinositol anchor attachment protein 1* (*GPAA1*), *phosphatidylinositol glycan class K* (*PIGK* or* GPI8*),* phosphatidylinositol glycan class S* (*PIGS*), and *phosphatidylinositol glycan class T* (*PIGT*) 5, 9. These subunits coordinate the final step of GPI-anchor biosynthesis by recognizing and cleaving the C-terminal signal sequence of substrate proteins and attaching the GPI anchor to a new terminus via a transamidation reaction 8, 9. GPI-APs are localized in the outer leaflet of the plasma membrane and are often enriched in lipid rafts, where they contribute to membrane trafficking, cell polarity, and signal transduction 6, 7. Several GPI-APs have been implicated in cancer progression 10, suggesting that dysregulation of their maturation machinery, such as GPI-T subunits, may contribute to tumorigenesis 10-14.

Emerging evidence has supported the oncogenic potential of certain GPI-T subunits. For instance, PIGU is overexpressed in bladder cancer and promotes uroepithelial cell malignancy via the urokinase receptor/signal transducer and activator of transcription 3 axis 11, whereas GPAA1 amplification enhances GPI-APs expression and activates lipid raft-mediated epidermal growth factor receptor/Erb-B2 receptor tyrosine kinase 2 signaling in gastric cancer 14. In HNC, a transcriptomic study focusing on three GPI-T subunits (PIGU, PIGT, and GPAA1) has found elevated *GPAA1* expression and genomic copy number gain in tumor tissues 12. Another functional study has demonstrated that exposure to cigarette smoke extract upregulated GPAA1 and activated epidermal growth factor receptor signaling, thereby promoting HNC cell proliferation and invasion 13. However, no study has comprehensively evaluated the expression and functional relevance of all GPI-T subunits in HNC. In this study, we applied a multi-omics approach to systematically investigate the prognostic value, molecular functions, and microenvironmental associations of GPI-T subunits in HNC, with a particular focus on PIGK as a potential oncogenic driver.

## Materials and Methods

### Data from The Cancer Genome Atlas (TCGA) and Gene Expression Omnibus

RNA sequencing (RNA-seq) data and clinical information of 523 HNC and 44 healthy samples were obtained from cBioPortal (https://www.cbioportal.org/) 15 and the UCSC Xena platform (https://xena.ucsc.edu/) up to February 2025, as previously described 16. Gene expression levels were quantified using RNA-Seq by Expectation-Maximization. Clinical variables from the cBioPortal included age at diagnosis, HPV status, histologic grade, disease status, time-to-event intervals, and survival outcomes. Additional data, such as anatomical subsite, American Joint Committee on Cancer (AJCC) T and N classifications, stage, and substance use history, were obtained from UCSC Xena. Patients with distant metastases at diagnosis (*n* = 5) or incomplete clinical data (*n* = 28) were excluded, yielding a final cohort of 490 patients. Differential gene expression between tumor and normal tissues was evaluated using the GSE23036 dataset (63 HNC and five normal tissues) from the Gene Expression Omnibus (https://www.ncbi.nlm.nih.gov/geo). GSE41613 (97 oral cancer cases) was used as an independent cohort for survival validation.

### Clinical specimens and tissue microarrays for HNC

Formalin-fixed, paraffin-embedded tumors and matched adjacent non-cancerous tissues (>1 cm from the tumor margin) were collected from 100 patients with histologically confirmed HNC treated at Kaohsiung Veterans General Hospital, Taiwan, between 2010 and 2016. All patients had nonmetastatic disease at diagnosis and underwent surgical resection. The tumor sites included the oral cavity, oropharynx, hypopharynx, and larynx. Clinical variables recorded included age, sex, AJCC T/N classification, stage, substance use, treatment strategy, and disease status. Ethical approval, including a waiver of informed consent, was granted by the Ethics Committee of Kaohsiung Veterans General Hospital (approval no. KSVGH23-CT8-10).

### Immunohistochemistry (IHC)

IHC staining was performed according to the manufacturer's instructions, as previously described 17. A primary antibody targeting PIGK (1:200; GTX105967; GeneTex) was used for detection. Quantification of PIGK expression was conducted using the HistoQuest analysis software (TissueGnostics, version 7.1), which incorporates a deep-learning-based nuclear segmentation algorithm. Based on the percentage of PIGK-positive tumor cells, protein expression was categorized into high (>10% positive cells) and low (≤10% positive cells) expression groups.

### Genomic alteration analysis

Genetic alterations and copy number variations in *PIGK* were analyzed using the cBioPortal database based on TCGA PanCancer Atlas and HNC datasets. Copy number alterations (CNA) were stratified according to the GISTIC2-defined categories to evaluate their effects on *PIGK* mRNA expression. The correlation between *PIGK* expression and Log_2_-transformed copy number was assessed in TCGA/HNC cohort. The "Plots" module was used to examine the association between *PIGK* levels and tumor mutation burden (TMB). TIMER 2.0 (http://timer.cistrome.org/) 18 was used to compare *PIGK* expression between the wild-type and mutant groups of the ten most frequently mutated genes in TCGA/HNC. Statistical differences were assessed using the Wilcoxon rank-sum test, with a *P*-value < 0.05 considered significant.

### Function and pathway analyses of the co-expressed genes

The LinkedOmics database (http://linkedomics.org/admin.php) 19 identified *PIGK*-co-expressed transcripts in the TCGA/HNC cohort (*n* = 517), with filtering criteria of *P*-value < 0.01, false discovery rate (FDR) < 0.01, and absolute correlation coefficient > 0.3. Gene Ontology (GO) enrichment analyses were performed using the DAVID platform (https://davidbioinformatics.nih.gov/summary.jsp) 20. Significant terms were defined by a Benjamini-Hochberg FDR < 0.01 and ranked accordingly. Ingenuity Pathway Analysis (IPA QIAGEN 2000-2024) was used to explore disease associations, functional implications, and canonical pathways. IPA terms were considered biologically relevant if *P*-value < 0.01 and |Z-score| > 2, and were ranked based on the predicted activation Z-scores.

### Protein-protein interaction (PPI) network construction and enrichment analysis

The BioGRID (https://thebiogrid.org/) 21 database was used to identify protein interactions with PIGK. We then selected interactors that showed a significant expression correlation with *PIGK* in the TCGA/HNC cohort using the TIMER2.0 database, with tumor purity adjusted. The Metascape (https://metascape.org/gp/index.html) platform 22 was used to further refine the list by selecting proteins with STRING physical interaction scores > 0.132. The resulting PPI network was visualized using Cytoscape (https://cytoscape.org/). GO and pathway enrichment analyses were performed via Metascape using cumulative hypergeometric testing with Benjamini-Hochberg correction. Terms with *P*-value < 0.01, FDR < 0.01, enrichment factor > 1.5, and gene count ≥ 3 were considered significant. Molecular Complex Detection (MCODE) analysis was used to identify the densely connected sub-networks 22. Each cluster was annotated based on the top three enriched terms ranked by the *P*-value, and the cluster containing PIGK was selected for further analysis.

### Identification and analysis of the potential interactors with PIGK

To explore potential functional partners of PIGK, we cross-referenced genes from the MCODE-derived cluster containing PIGK and PIGK-associated molecules from the Pathway Commons database (https://www.pathwaycommons.org/) 23. The expression profiles of the overlapping candidates were visualized using TIMER2.0, and correlation and survival analyses were performed using GEPIA2 (http://gepia2.cancer-pku.cn/) 24.

### Tumor immune microenvironment analysis

The TIMER2.0 platform 18 was used to evaluate the associations between *PIGK* expression and the abundance of immune and stromal cell populations in bulk tumors from the TCGA/HNC cohort using the CIBERSORT and EPIC deconvolution algorithms. Correlation analyses were conducted using Spearman's method. Patients were stratified into *PIGK*-high and *PIGK*-low groups based on the median values. Kaplan-Meier survival analysis was performed to assess the prognostic relevance of the selected immune and stromal cell types within each subgroup. Cancer-associated fibroblast (CAF) scores were calculated as the average expression of decorin (*DCN*), podoplanin (*PDPN*), and fibroblast activation protein alpha (*FAP*), as previously described 25. For single-cell analysis, the TISCH2 database (http://tisch.comp-genomics.org/) 26 was used to explore *PIGK* expression across the annotated cell types in the GSE103322 dataset. The same resource was employed for gene set enrichment analysis and cell-cell interaction profiling. A *P*-value < 0.05 was considered statistically significant for all analyses.

### Cell lines and cell viability assay

The human HNC cell line TW1.5 was maintained in Dulbecco's modified Eagle medium/Ham's F-12 nutrient mixture medium, SAS in Dulbecco's modified Eagle medium, and supplemented with 10% fetal bovine serum (FBS). MRC-5 (fibroblast) cells were maintained in α-minimum essential medium (α-MEM; HyClone™, Cat. #SH30008.02, USA) supplemented with 0.1 mM non-essential amino acids, 1 mM sodium pyruvate, and 10% FBS. For cell viability assay, cells (5,000 cells) were plated in a 96-well plate with complete medium overnight at 37°C in 5% CO_2_. Following incubation, eight replicates of each concentration of various chemotherapeutic drugs were added to the wells, which were then incubated for 72 h. Next, MTT assay (#101-298-93-1, Cyrus Bioscience) was performed for 4 h, cell proliferation was assessed, and signals were measured using an ELISA reader (Thermo Fisher Scientific; Multiskan FC). Cisplatin (HY-17394), paclitaxel (HY-B0015), and docetaxel (HY-B0011) were purchased from MCE.

### Lentivirus infection

Lentiviral supernatants containing either the vector control (pLKO.1-shLuc967) or shPIGK shRNAs (TRCN0000050118 and TRCN0000050120) were obtained from the National RNAi Core Facility (Taipei, Taiwan). TW1.5 cells were infected with the viral supernatants in the presence of 8 μg/ml polybrene. After 72 h of infection, cells were selected with 2 μg/ml puromycin to establish stable knockdown cell lines.

### Reverse transcription-quantitative polymerase chain reaction (RT-qPCR)

According to the manufacturer, TRIzol® (Thermo Fisher Scientific, Inc.; #15596018) was used to extract total RNA from HNC cells. Following the manufacturer's instructions, cDNA was generated using the PrimeScriptTM RT Reagent kit (#RR037A; Takara Biotechnology Co., Ltd.). To assess PIGK expression, qPCR was conducted using a SYBR Green PCR Master Mix (PCR Biosystems Ltd. qPCRBIO SyGreen Mix Lo-ROX). The following primer sequences were used: *PIGK*, forward: 5′-ACTCCTCGGTCAAAACGTCTT-3′; *PIGK*, reverse: 5′-CCGCGAGTTCTATGTTGGTAAT-3′; *FAM20C*, forward: 5′-CCTTCCAGAATTACGGGCAAG-3′; *FAM20C*, reverse: 5′-TGCCTCTCGTAGTCAGAGAAAT-3′; *ACTA2*, forward: 5′-CCAACTGGGACGACATGGAA-3′; *ACTA2*, reverse: 5′-ATTTTCTCCCGGTTGGCCTT-3′; *VIM*, reverse: 5′-GCTAACCAACGACAAAGCCC-3′; *VIM*, reverse: 5′-GATTGCAGGGTGTTTTCGGC-3′; *ACTB*, forward: 5′ AGAAAATCTGGCACCACACC-3′ and *ACTB*, reverse: 5′ AGAGGCGTACAGGGATAGCA-3′.

### Cell invasion and migration assay

The migratory and invasive abilities of TW1.5/shluc, TW1.5/shPIGK-1, and TW1.5/shPIGK-2 cells were determined using Boyden chambers, as previously described 17. First, the cells were resuspended in serum-free medium (3 × 10^5^ cells/ml), and 50 μl of suspension was loaded into the upper chamber. After 24 h, the cells were stained with crystal violet and measured under a light microscope.

### Colony formation assay

A density of 1 × 10^4^ cells/well stable lines was seeded into a six-well plate. The cells were fixed and stained with crystal violet after seven days. The NIH Image J software was used to count the colonies.

### Statistical analysis

Statistical comparisons were performed using parametric and nonparametric tests. Student's *t*-test and chi-square test were used for continuous and categorical variables, respectively, whereas the Wilcoxon rank-sum test and Kruskal-Wallis test were used for nonparametric analysis. Correlations between variables were evaluated using Spearman's rank correlation coefficient. The prognostic significance of the clinical and molecular features was assessed using univariate Cox regression analysis with hazard ratios (HRs) and 95% confidence intervals (CI) reported. Survival distributions were estimated using the Kaplan-Meier method, and group differences were compared using the log-rank test. A two-sided *P*-value of less than 0.05 was considered statistically significant.

## Results

### PIGK is the GPI-T subunit consistently upregulated in HNC across independent transcriptomic datasets

**Figure 1:**
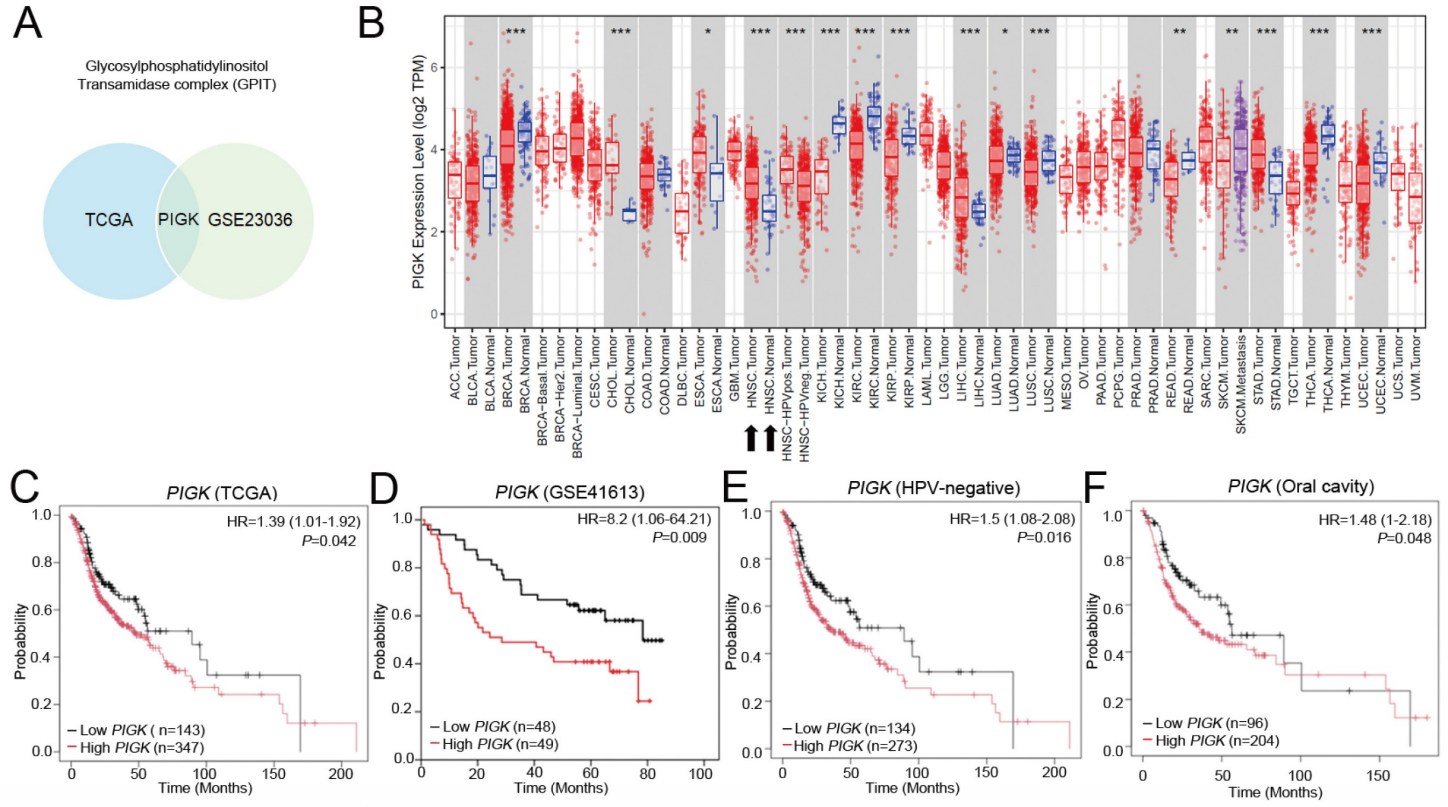
** Transcriptomic expression and survival analyses of *PIGK* in head and neck cancer.** (A) Venn diagram illustrating differentially expressed GPI-T subunits between tumor and normal tissues in the TCGA/HNC and GSE23036 cohorts. (B) *PIGK* levels in tumors and normal tissues across various cancer subtypes in TCGA dataset (TIMER 2.0). (C) Kaplan-Meier analysis of overall survival (OS) based on *PIGK* levels in the TCGA/HNC cohort. (D) OS analysis of* PIGK* levels in the GSE41613 validation cohort. (E-F) Subgroup OS analyses in HPV-negative (E) and oral cavity (F) subgroups in the TCGA/HNC cohort.

As previous studies have demonstrated that members of the GPI-T play essential roles in the occurrence and development of malignancies, we compared the transcript levels of five GPI-T subunits (GPAA1, PIGK, PIGS, PIGT, and PIGU) between tumor and normal tissues using two independent datasets, GSE23036 and TCGA/HNC. Among these, only *PIGK* was consistently upregulated in tumor tissues across both datasets (Fig. 1A, Supplementary Fig. S1A and B), suggesting its potential oncogenic relevance. In TCGA transcriptome data visualized using TIMER 2.0, *PIGK* was found to be aberrantly expressed across multiple cancer types. In HNC, *PIGK* levels were significantly higher in tumor tissues (*n* = 520) than in normal tissues (*n* = 44) (*P* < 1 × 10^-3^) (Fig. 1B), supported by the TNMplot gene-chip data (Supplementary Fig. S1C and D). The Human Protein Atlas database also indicates that *PIGK* is moderately expressed in normal tongue and salivary gland tissues, which are representative anatomical sites in the head and neck region, suggesting that *PIGK* may not be entirely tumor-specific (Supplementary Fig. S1E).

Further correlation analyses demonstrated that *PIGK* levels were associated with a positive N classification (*P* = 4.23 × 10^-2^), poor histological differentiation (*P* = 6.38 × 10^-4^), HPV-positive status (*P* < 1 × 10^-5^), and non-oral cavity subsites (*P* =1.31 × 10^-4^) (*n* = 490; Table 1). Kaplan-Meier survival curves showed that low *PIGK*, with a cut-off value of 3.32, was associated with better overall survival (OS) (HR = 1.39, 95% CI: 1.01-1.92, *P* = 4.2 × 10^-2^) (Fig. 1C), a result supported by the GSE41613 dataset (HR = 8.2, 95% CI: 1.06-64.21, *P* = 9 × 10^-3^) (Fig. 1D). Subgroup analyses showed its prognostic significance in both HPV-unrelated patients (*n*= 407; HR = 1.5, 95% CI: 1.08-2.08, *P* = 1.6 × 10^-2^) (Fig. 1E) and those with oral cavity cancers (*n* = 300; HR = 1.48, 95% CI: 1-2.18,* P* = 4.8 × 10^-2^) (Fig. 1F). Additional analyses of disease-specific survival (DSS) and progression-free survival are illustrated in Supplementary Fig. S2A and B.

### Protein-level validation confirms the clinical relevance of PIGK expression in HNC

**Figure 2:**
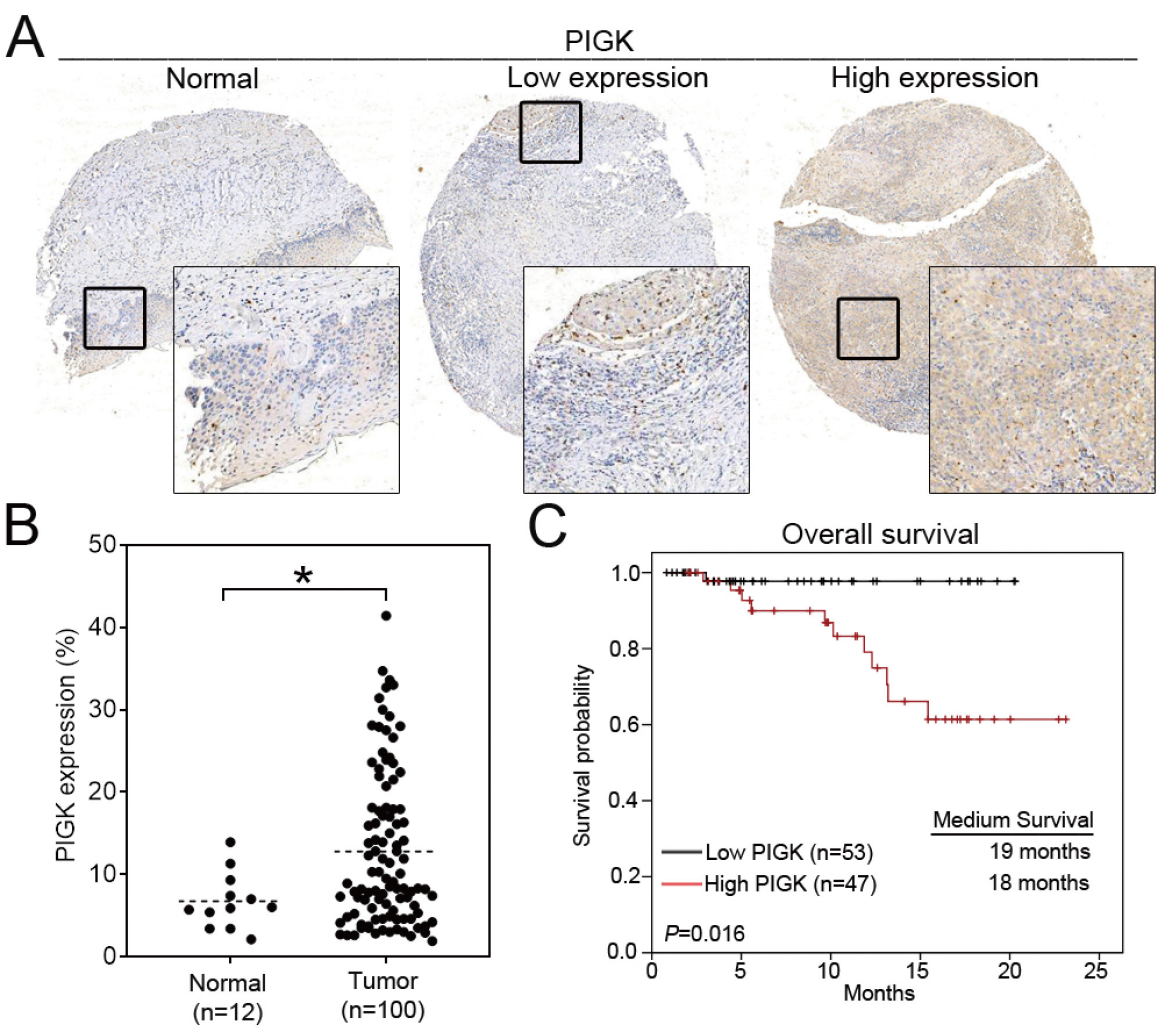
** Immunohistochemical (IHC) analysis of PIGK in HNC tissues. (**A) Representative IHC staining of PIGK in paired tumor and adjacent normal tissues. (B) Quantitative comparison of PIGK protein expression levels in tumor (*n* = 100) and normal tissues (*n* = 12). (C) Overall survival analysis by PIGK protein expression in the TMA cohort. Statistical significance: *P < 0.05.

To validate the transcriptome-based findings, we performed IHC staining of PIGK in a tissue microarray consisting of tumor (*n* = 100) and normal (*n* = 12) tissues. Consistent with the transcriptomic data, PIGK protein expression was significantly higher in tumor tissues (*P* = 2.4 × 10^-2^, Fig. 2A and B). Clinical association analysis showed that high PIGK expression (*n* = 47) was more likely to have a higher AJCC T stage (*P* = 1.1 × 10^-2^), nodal metastasis (*P* = 1.7 × 10^-2^), advanced overall stage (*P* = 4.8 × 10^-2^), and a higher risk of recurrence (*P* = 4.7 × 10^-2^) (Table 2). Although not statistically significant, the high PIGK group also showed trends toward greater tumor thickness (mean: 5.56 mm, *P* = 8.2 × 10^-2^) (Table 2). Kaplan-Meier analysis demonstrated that patients with high PIGK expression had significantly worse OS (*P* = 1.6 × 10^-2^) (Fig. 2C). Univariate Cox analysis further demonstrated that high PIGK expression (*P* = 4.3 × 10^-2^), advanced T (*P* = 1× 10^-2^), and positive N classification (*P* = 2.9 × 10^-2^) were significantly associated with poor OS (Table 3). Collectively, these results support the clinical relevance of PIGK in HNC and suggest that its overexpression is correlated with more aggressive disease features and poorer survival outcomes.

### Analysis of genetic alterations of PIGK in HNC

**Figure 3:**
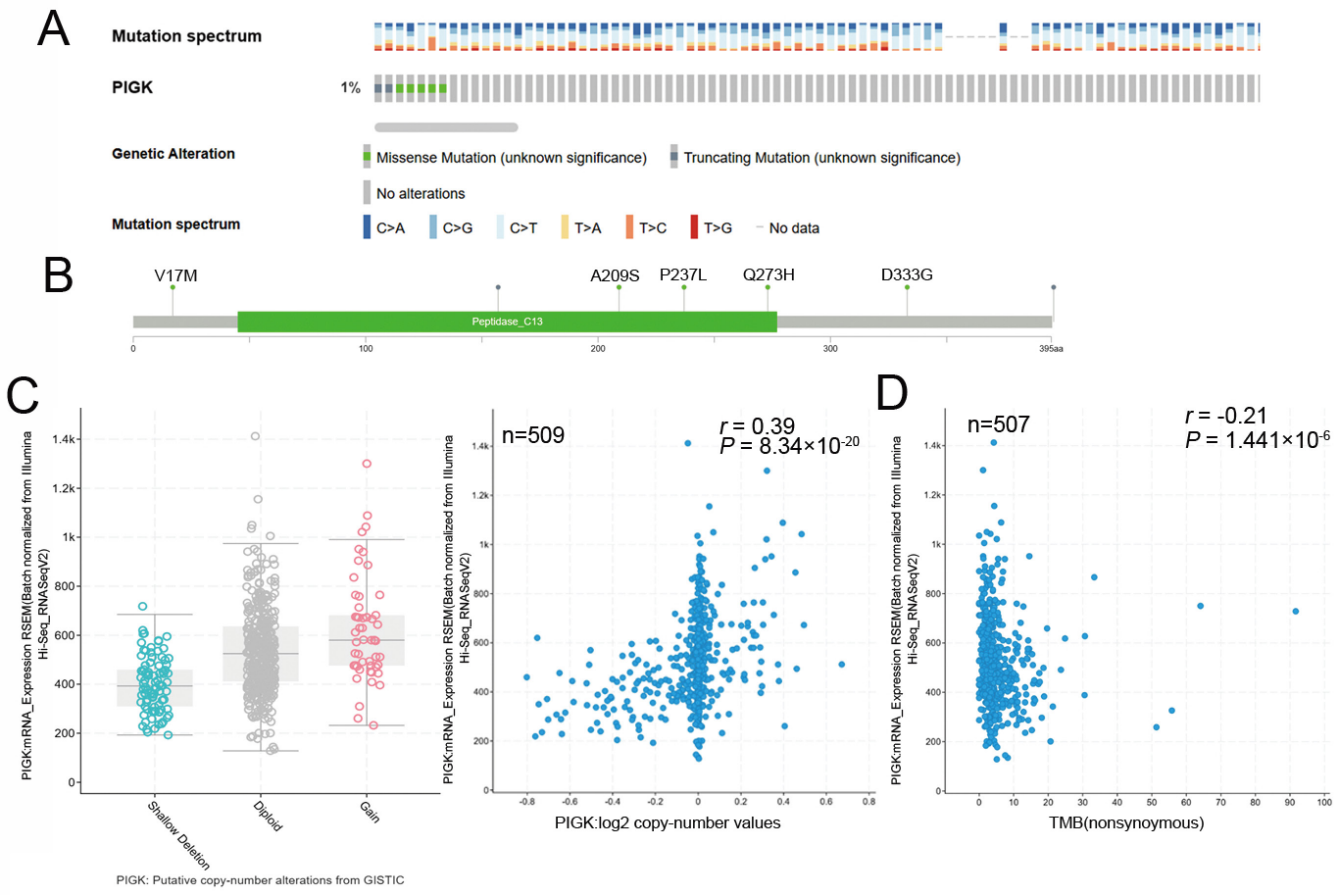
** Genomic alterations of *PIGK* in HNC.** cBioPortal shows the frequencies (A) and types (B) of *PIGK* genetic alterations in the TCGA/HNC cohort. (C) *PIGK* mRNA expression (RSEM) stratified by GISTIC2-defined copy number alteration (CNA) categories (left panel) and correlation with Log2 copy number values (right panel) (*n* = 509). (D) Correlation between *PIGK* level (RSEM) and tumor mutation burden (TMB) in TCGA/HNC (*n* = 507).

Using the cBioPortal database, we analyzed *PIGK* genetic alterations across various cancer subtypes in the TCGA PanCancer Atlas Studies dataset. The alteration frequency was 2% in 2922 samples, and the most common variation type was amplification (*n* = 32) (Supplementary Fig. S3A and B). In the TCGA/HNC cohort, the alteration rate was 1%, primarily consisting of missense mutations (V17M, A209S, P237L, Q273H, and D333G) (Fig. 3A and B). To explore potential mechanisms underlying *PIGK* overexpression, we analyzed GISTIC2-defined CNA in 509 patients with HNC and found a significant positive correlation between PIGK expression and log₂ copy number values (*r* = 0.39, *P* = 8.34 × 10^-20^) (Fig. 3C), suggesting that copy number gain may partially account for *PIGK* upregulation.

Additionally, *PIGK* mRNA levels were inversely correlated with TMB (*r* = -0.21, *P* = 1.44 × 10^-6^) (Fig. 3D), suggesting that *PIGK* may be associated with a low-mutation phenotype. To further investigate this association, we assessed *PIGK* levels across the mutation status of the ten most frequently altered cancer genes in TCGA/HNC cohort, including *TP53* (69.3%), *FAT1* (21.6%), *CDKN2A* (20.4%), *PIK3CA* (17.5%), *NOTCH1* (17.1%), *LRP1B* (16.5%), *PCLO* (15.3%), *KMT2D* (15%), *NSD1*(11.7%), and *CASP8* (10.7%). These mutations are predominantly found in HPV-negative HNC. TIMER2.0 analysis showed that *PIGK* levels were significantly higher in the wild-type groups of *FAT1* (Wilcoxon rank-sum test, *P* = 3.1 × 10^-2^), *CDKN2A* (*P* = 3.7 × 10^-3^), *NOTCH1* (*P* = 1.4 × 10^-2^), and *CASP8* (*P* = 1.9 × 10^-4^) (Supplementary Fig. S4A-D). These findings suggest that the lower *PIGK* levels in HPV-negative HNC may be partially explained by the higher mutation burden and frequent alterations in tumor suppressor genes in this subgroup.

### Functional and pathway analyses of PIGK co-expressed genes implicate its oncogenic potential in HNC

**Figure 4:**
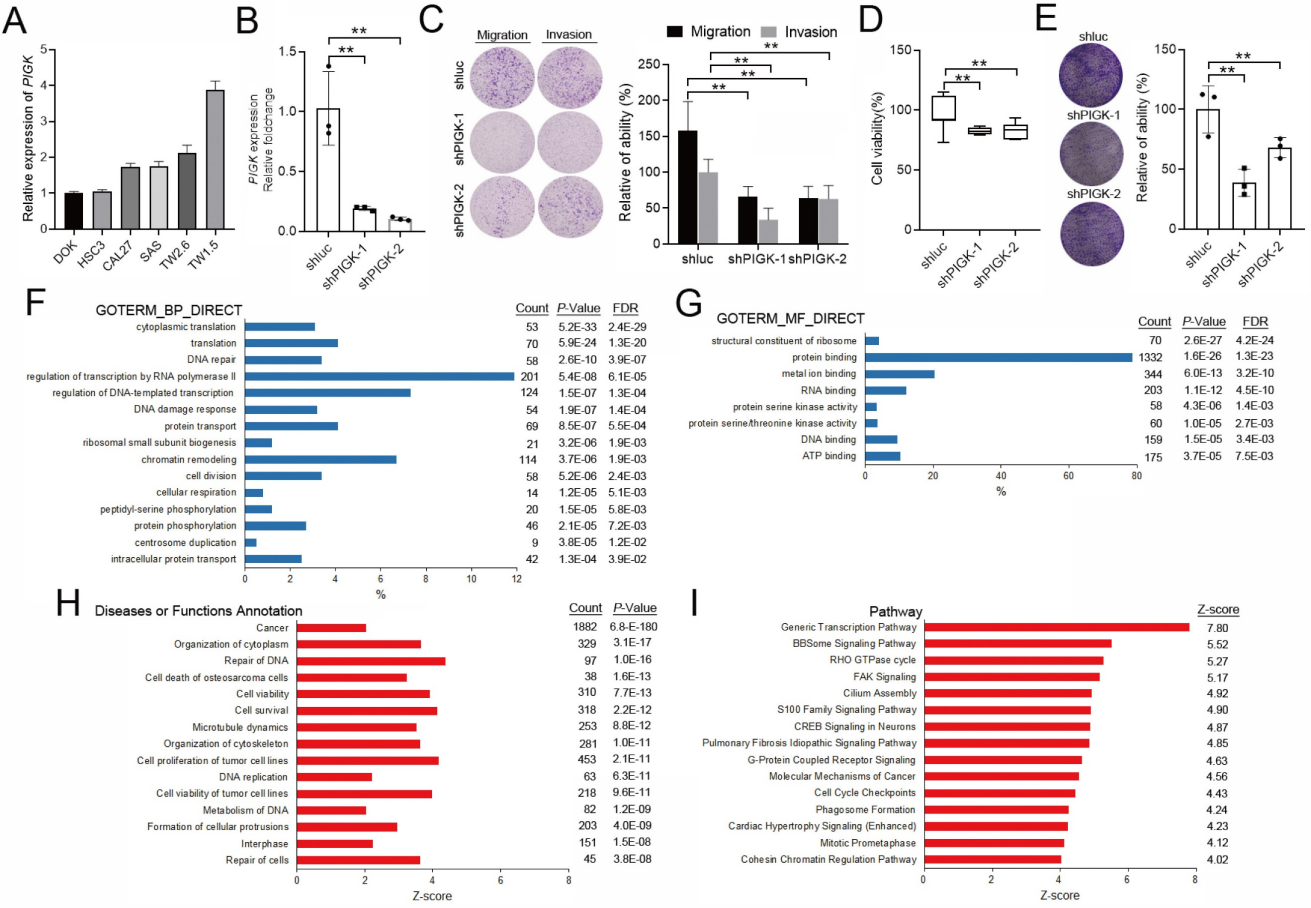
** Functional and pathway enrichment analyses of *PIGK* co-expressed genes.** (A) RT-qPCR measured the endogenous *PIGK* levels in six HNC cell lines. (B) RT-qPCR analysis of *PIGK* expression in TW1.5 after lentiviral-mediated RNA interference. The effect of PIGK knockdown on migration, invasion (C), colony formation assay (D), and cell viability (E) was evaluated in TW1.5 cells infected with shluc or shPIGK. Data were presented as the mean ± SD; **P < 0.01; *P < 0.05. (F) Gene Ontology (GO) Biological Process (BP) terms enriched among *PIGK* co-expressed transcripts (*n* = 517, LinkedOmics). Top-ranked terms with FDR < 0.01 are shown. (G) GO Molecular Function (MF) enrichment showing representative terms. (H) Ingenuity Pathway Analysis (IPA) disease/function annotation of *PIGK* co-expressed genes, which are ranked by *P*-value. (I) IPA canonical pathway analysis identifying enriched cancer-related signaling pathways, ordered by activation Z-score.

We evaluated the effects of PIGK knockdown on the migration, invasion, colony formation, and proliferation abilities of HNC cells. To validate *PIGK* expression, six HNC cell lines with different* PIGK* expression levels were screened, and SAS (low *PIGK*) and TW1.5 (high *PIGK*) were selected (Fig. 4A). Knockdown of PIGK expression in TW1.5 cells significantly reduced their cell viability, migration, invasion, and colony formation abilities compared with shluc control cells (Fig. 4B-E). Next, to explore the functional relevance of PIGK in HNC, we identified 1963 transcripts co-expressed with *PIGK* using the LinkedOmics database, based on the criteria of |correlation coefficient| > 0.3, *P* < 0.01, and FDR < 0.01 (Supplementary Table 1). GO enrichment analysis was performed using the DAVID database. The top-ranked biological processes included “cytoplasmic translation (GO:0002181, FDR = 2.4×10^-29^),” and “DNA repair (GO:0006281, FDR = 3.9 × 10^-7^)” (Fig. 4F). For molecular function, enriched terms included “structural constituent of ribosome (GO:0003735, FDR = 4.2 × 10^-24^)”, and “protein binding (GO:0005515, FDR = 1.3 × 10^-23^)” (Fig. 4G).”

IPA further revealed that 1,882 of these genes (95.9%) were annotated under the disease/function category “cancer,” which showed a high significance (Z-score = 2.05, *P* = 6.8 × 10^-^¹⁸⁰) (Fig. 4H, Supplementary Table 2). Pathway analysis further identified several cancer-related signaling pathways with activation predictions, among which the “Generic Transcription Pathway (Z-score = 7.80)” and “BBSome Signaling Pathway (Z-score = 5.52) ranked highest. The “Molecular Mechanism of Cancer (Z-score = 4.56)” was also among the enriched terms (Fig. 4I), supporting the notion that PIGK may contribute to tumor progression in HNC by involving multiple cancer-associated signaling and regulatory pathways.

### PIGK-associated PPI network highlights GPI-anchor biosynthesis in HNC

**Figure 5:**
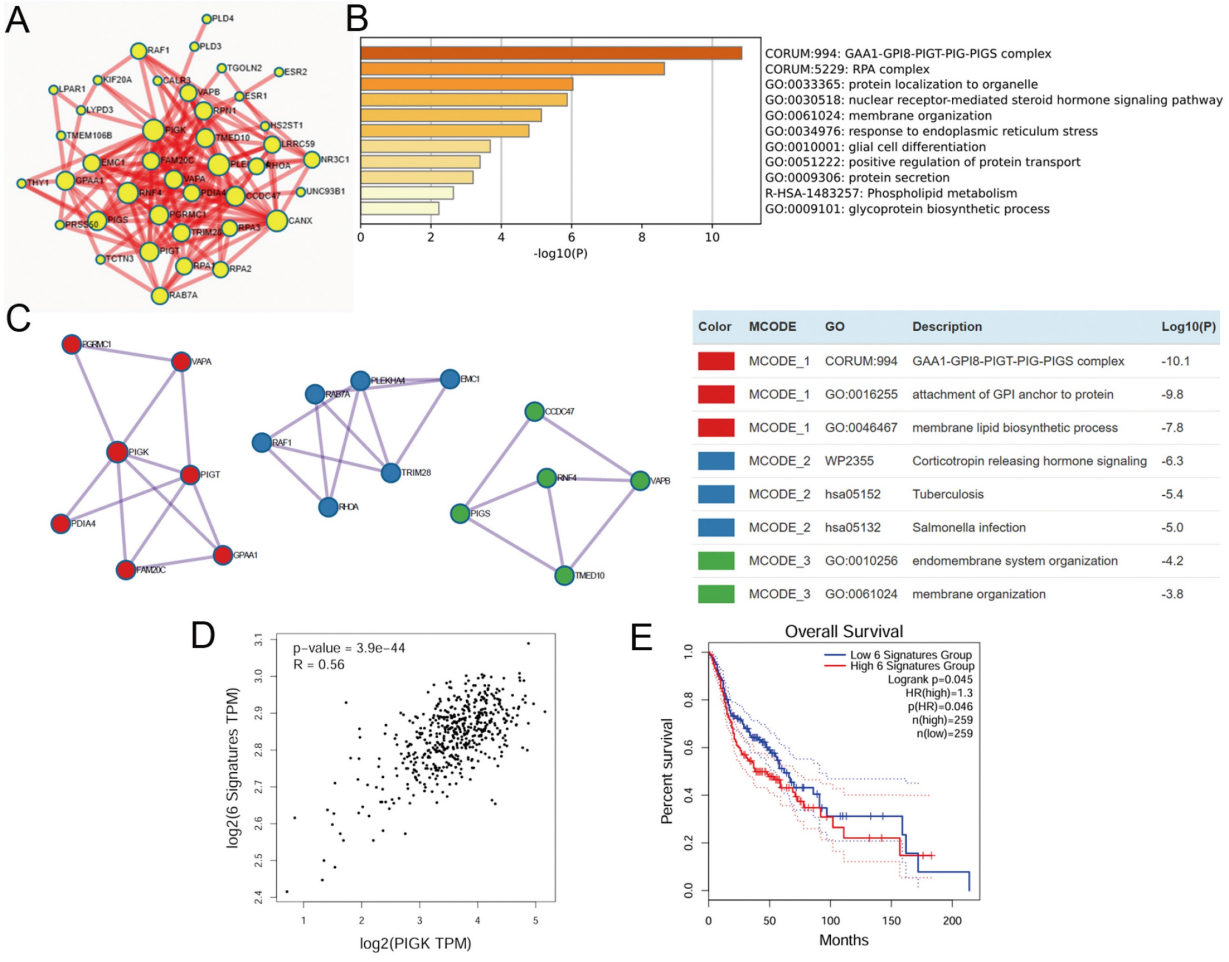
** Construction and enrichment analysis of PIGK-associated protein-protein interaction (PPI) network in HNC.** (A) PPI network of 40 physical interactors of PIGK identified via Metascape with STRING interaction score > 0.132. (B) Enriched terms from the PPI network (FDR < 0.01). (C) Molecular Complex Detection (MCODE) clustering of the PPI network, identifying three sub-networks (left panel). The top three enriched terms for each cluster. Red denotes the cluster containing PIGK (right panel). (D) Correlation between a six-gene signature derived from the PIGK-containing cluster and *PIGK* expression. (E) Kaplan-Meier overall survival analysis stratified by the signature score.

Building on our co-expression analysis implicating PIGK in HNC pathogenesis, we explored its protein-level interactions. A total of 76 experimentally validated PIGK-interacting proteins were retrieved from the BioGRID database (Supplementary Table 3). Of these, 56 were significantly correlated with PIGK in TCGA/HNC cohort after adjusting for tumor purity (Supplementary Table 4). To refine the network, we used Metascape to select physical interactors with a STRING interaction score > 0.132, yielding 40 proteins for the PPI network construction (Fig. 5A, Supplementary Table 5). Enrichment analysis of the PPI network (FDR < 0.01) revealed significant enrichment in “protein localization to organelle (GO:0033365),” “response to endoplasmic reticulum stress (GO: 0034976),” and “glycoprotein biosynthetic process (GO: 0009101)” (Fig. 5B), which are partly consistent with transcriptome-level findings.

Subsequent MCODE clustering identified three sub-networks (Fig. 5C), with PIGK localized in a cluster enriched in terms such as “GAA1-GPI8-PIGT-PIG-PIGS complex (CORUM:994),” “attachment of GPI anchor to protein (GO:0016255),” and “membrane lipid biosynthetic process (GO:0046467)” (Fig. 5C). To assess the clinical relevance of this cluster, we constructed a six-gene signature based on its members (*VAPA*,* PIGT*, *GPAA1*, *PGRMC1*, *FAM20C*, and *PDIA4*), which showed a significant positive correlation with *PIGK* (*r* = 0.56, *P* = 3.9 × 10^-44^) (Fig. 5D) and was modestly associated with worse OS (*P* = 4.5 × 10^-2^) (Fig. 5E). Additionally, *PIGK* levels positively correlated with other GPI-T subunits (*PIGU*, *PIGT*,* GPAA1,* and *PIGS*) (Supplementary Figure S5A-D). Given that GPI-anchored proteins are typically localized in lipid rafts that organize oncogenic signaling 8-10, we further examined the association between *PIGK* levels and a lipid-rapid-related signature (*STOM*, *PHB1*, *FLOT1*, *FLOT2*,* CAV1*, *CAV2*, and *CAV3*), which revealed a significant positive correlation (*r* = 0.46, *P* = 5.7 × 10^-28^) (Supplementary Fig. S5E). Collectively, these findings suggest that PIGK promotes HNC progression through its involvement in GPI-anchor biosynthesis and membrane-associated signaling pathways that modulate the localization or activity of signaling proteins.

### FAM20C is a potential interactor of PIGK associated with prognosis in HNC

**Figure 6:**
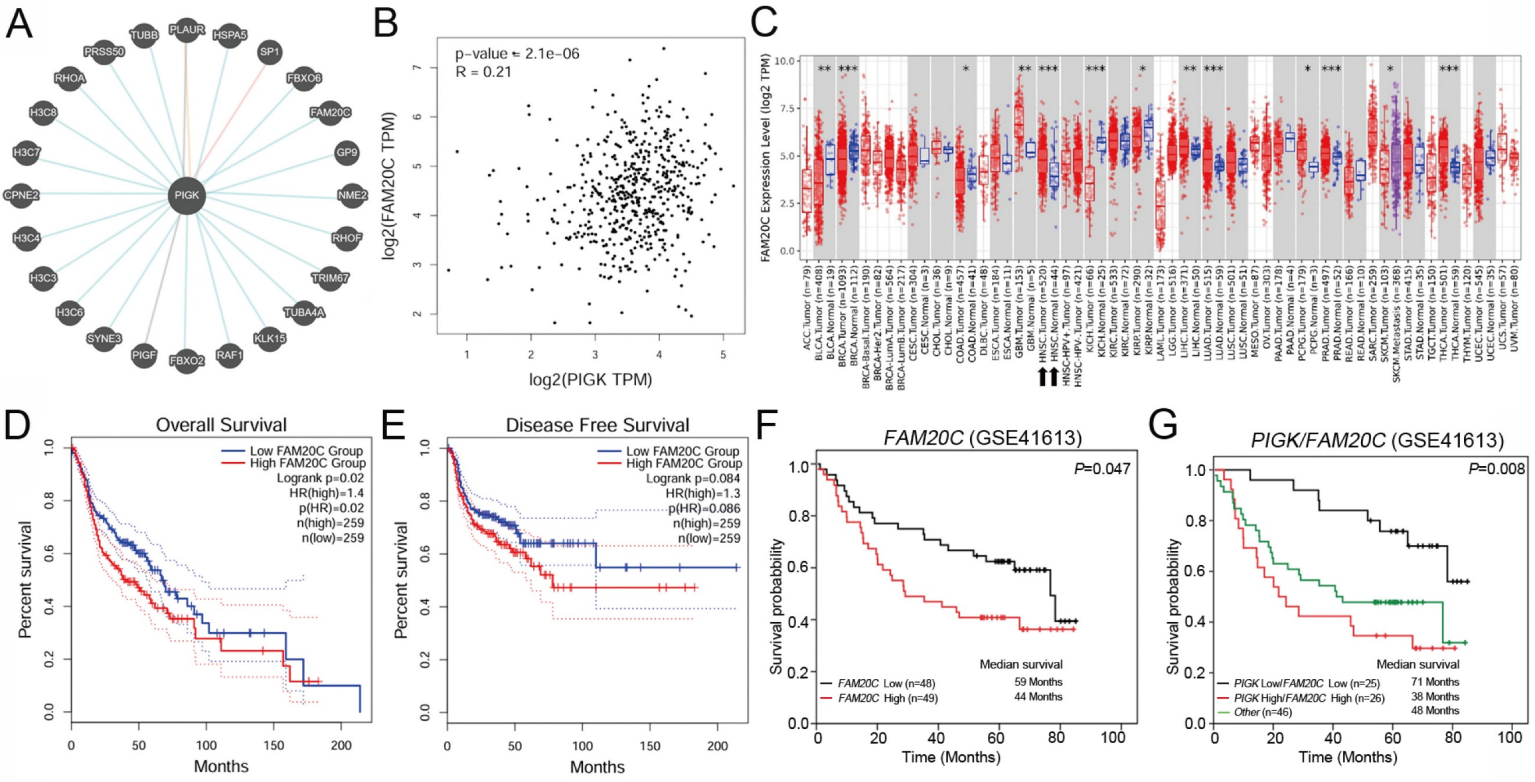
** FAM20C is a potential PIGK interactor associated with poor prognosis in HNC.** (A) PIGK-associated genes from Pathway Commons database. (B) Correlation between *PIGK* and* FAM20C* levels in the TCGA/HNC cohort. (C) *FAM20C* levels in multiple cancers from TCGA cohort. Kaplan-Meier survival analysis showing overall survival (OS) (D) and disease-free survival (DFS) (E) stratified by *FAM20C* levels in TCGA/HNC cohort. (F) OS analysis by *FAM20C* levels in the GSE41613 dataset. (G) Combined OS analysis based on *PIGK* and *FAM20C* expression levels in the GSE41613 dataset.

To identify functionally relevant PIGK interactors, we intersected six molecules (*VAPA*,* PIGT*, *GPAA1*, *PGRMC1*, *FAM20C*, and *PDIA4*) in the same MCODE cluster with PIGK-linked molecules in the Pathway Commons database (Fig. 6A), an integrative resource of curated pathways and interaction data 21. FAM20C emerged as the only overlapping gene, indicating its potential biological relevance. In the TCGA/HNC cohort, *PIGK* was positively correlated with *FAM20C* (*r* = 0.21, *P* = 2.1 × 10^-6^) (Fig. 6B). Additionally, *FAM20C* was significantly upregulated in tumor tissues compared with normal tissues (*P <* 1 × 10^-3^) and correlated with nodal metastasis (*P* = 4.53 × 10^-3^) and advanced overall stage (*P* = 5.04 × 10^-3^) (Fig. 6C, Table 4). Survival analysis revealed that patients with high *FAM20C* (*FAM20C*^high^) levels had significantly worse OS (log-rank *P* = 2 × 10^-2^, HR = 1.4), although disease-free survival showed only a marginal trend (*P* = 8.4 × 10^-2^) (Fig. 6D and E). These findings were validated in the GSE41613 cohort, where low *FAM20C* levels (*FAM20*C^low^) were associated with markedly better OS (*P* = 4.7 × 10^-2^) (Fig. 6F). Further combined expression analysis revealed that patients with *PIGK*^low^/*FAM20C*^low^ (*n* =25) had the most favorable OS, whereas *PIGK*^high^/*FAM20C*^high^ correlated with the poorest prognosis (*P* = 8 × 10^-3^) (Fig. 6G). Next, we further evaluated whether PIGK mediated FAM20C expression in HNC cells. Knockdown of *PIGK* significantly reduced *FAM20C* levels in TW1.5 cells (Supplementary Fig. S6). These results suggest that FAM20C might act in concert with PIGK and jointly contribute to HNC progression.

### PIGK expression is associated with stromal remodeling and adverse immune contexture in the HNC TME

**Figure 7:**
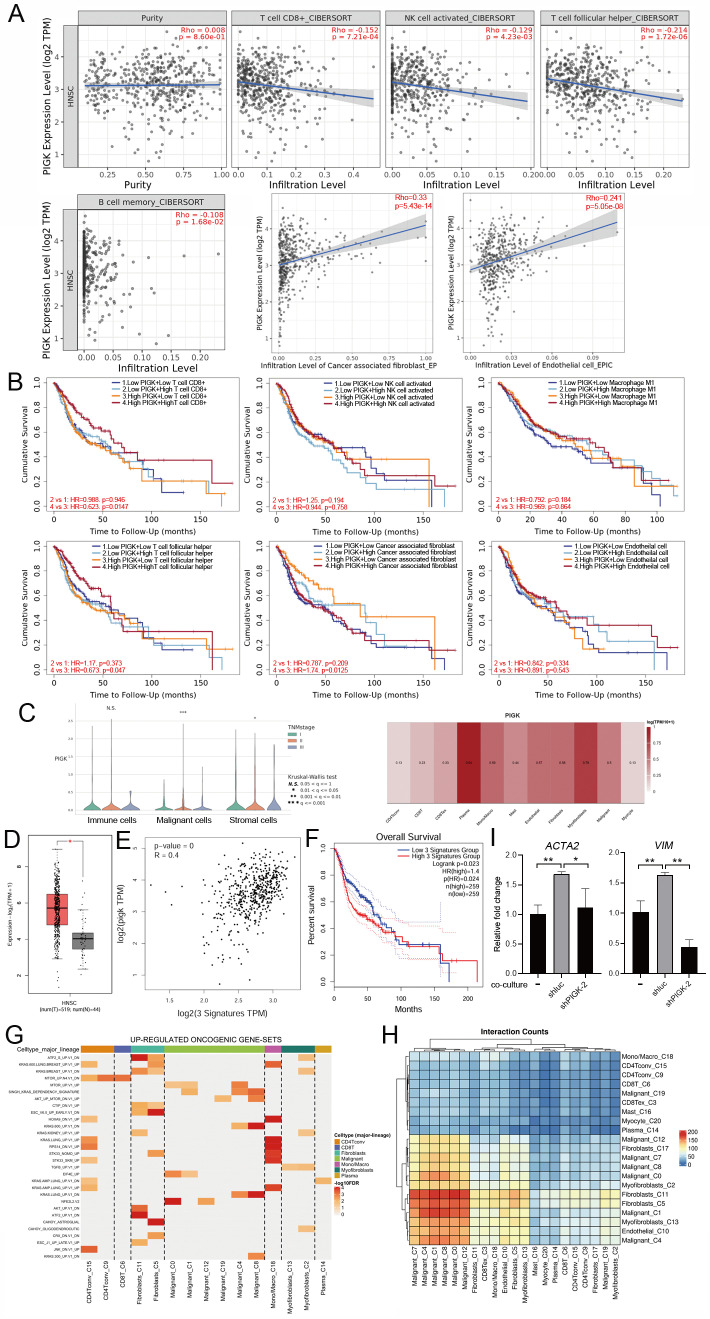
** PIGK expression is associated with stromal enrichment and altered immune infiltration in HNC.** (A) The TIMER2.0 database revealed a correlation of *PIGK* expression with immune and stromal cell infiltration estimated using CIBERSORT and EPIC algorithms in TCGA/HNC cohort (*n* = 522). (B) Survival analysis stratified by CD8⁺ T cell, NK activated cell, T follicular helper cell, memory B cells, cancer-associated fibroblast (CAF), and endothelial cell infiltration and *PIGK* levels in bulk tumors. (C) Single-cell RNA-seq analysis from the GSE103322 dataset showing *PIGK* levels correlation with tumor stage in malignant cells (left) and across cell types (right), based on TISCH2. (D-F) CAF scores based on decorin (*DCN*), podoplanin (*PDPN*), and fibroblast activation protein alpha (*FAP*) expression, showing differential expression between normal (*n* = 519) and tumor tissues (*n* = 44) (D), correlation with *PIGK* (E), and prognostic significance (F). (G) Gene set enrichment analysis showing numerous upregulated oncogenic signatures in fibroblast populations (TISCH2). (H) Cell-cell interaction analysis showing frequent interaction counts between malignant and fibroblast clusters (TISCH2). Statistical significance: *q < 0.05, **q < 0.01, ***q <0.001. (I) MRC-5 cells (fibroblasts) were co-cultured with TW1.5/shluc and TW1.5/shPIGK cells for 72 h, followed by RT-qPCR analysis. Data were presented as the mean ± SD; **P < 0.01; *P < 0.05.

To investigate the relationship between PIGK expression and TME in HNC, we analyzed bulk RNA-seq data from the TCGA/HNC cohort. Using the CIBERSORT and EPIC algorithms, we found that* PIGK* expression was inversely correlated with the infiltration of CD8⁺ T-, natural killer activated-, T follicular helper-, and memory B cells and positively correlated with CAFs and endothelial cells (Fig. 7A). Stratified survival analysis showed that in *PIGK*-high tumors, low CD8^+^ and T follicular helper cell infiltration and high CAF abundance were associated with worse survival, suggesting that the prognostic significance of these cell types may be influenced by *PIGK* expression (Fig. 7B).

Single-cell RNA-seq data from the GSE103322 dataset showed that *PIGK* expression in malignant cells increased with the tumor stage (Fig. 7C). Among the stromal and immune cell populations with prognostic significance, fibroblasts exhibited the highest *PIGK* expression (Fig. 7C). Given that CAFs are prominent in the tumor stroma of HNC and are known to exert immunosuppressive effects 27-29, we hypothesized that PIGK may be involved in CAF-mediated tumor-promoting processes. To test this, we analyzed CAF scores based on *DCN*, *PDPN*, and *FAP* expression, as previously described by Calon et al. 25. The three-gene signature was significantly higher in bulk tumors, positively correlated with *PIGK* (*r* = 0.4) and associated with poor prognosis (*P* = 2.3 × 10^-2^) (Fig. 7D-F). Functional enrichment analysis using TISCH2 revealed that the upregulated oncogenic gene sets were predominantly enriched in fibroblast (C5 and C11) and mono/macrophage (C18) populations (Fig. 7G). Moreover, cell-cell interaction analysis showed that malignant clusters (C0, C1, C4, C7, C8, and C12) had abundant interaction counts with fibroblast clusters (C5 and C11) and myofibroblast (C13) (Fig. 7H). We further examined the effect of PIGK on fibroblast activation. The RT-qPCR results showed CAF markers were downregulated in fibroblasts co-cultured with PIGK-knockdown cells compared with those co-cultured with control cells (Fig. 7I). These data support the hypothesis that PIGK-expressing tumor cells might interact with fibroblasts to shape an immunosuppressive TME and contribute to adverse survival outcomes in HNC.

### *PIGK* level correlates with the response to taxane-based chemotherapy in HNC

**Figure 8:**
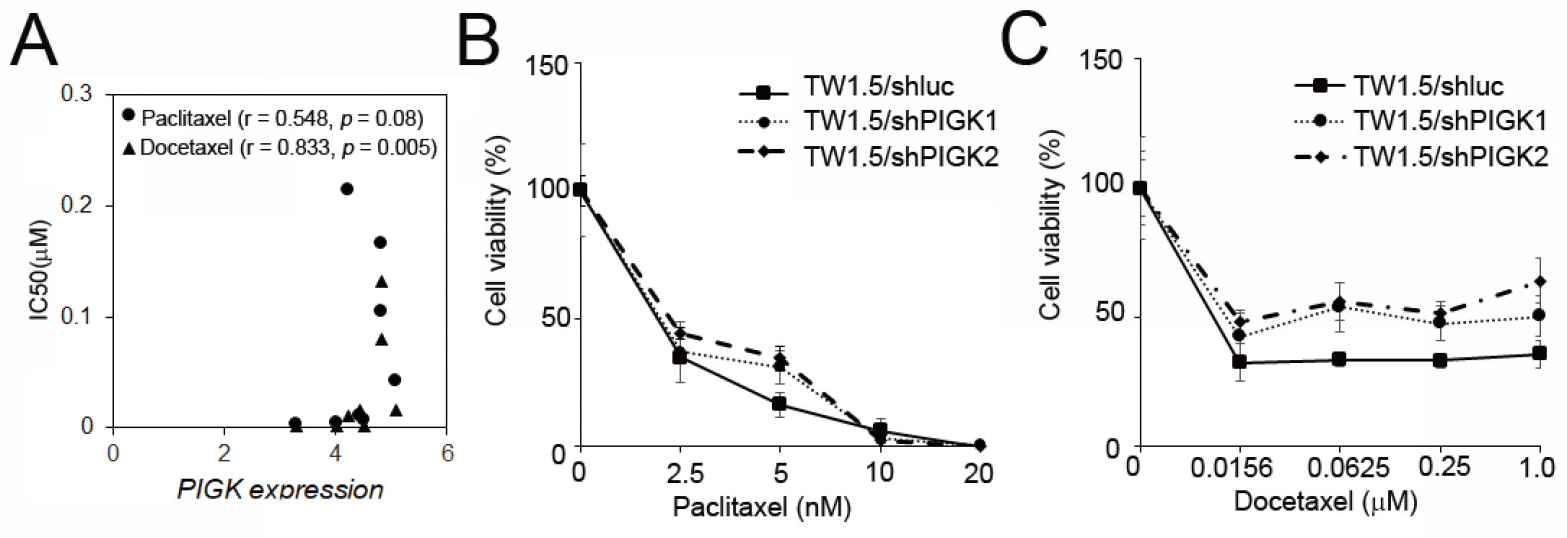
***PIGK* levels associated with chemotherapy drug response in HNC cell lines.** (A) *PIGK* levels and the IC_50_ values for paclitaxel and docetaxel in eight HNC cell lines. The data from Cancer Cell Line Encyclopedia (CCLE) and Genomics of Drug Sensitivity in Cancer (GDSC) databases. MTT assay showed the cell viability of TW1.5/shPIGK and TW1.5/shluc cells after treatment with paclitaxel (B) and docetaxel (C).

To assess the potential link between* PIGK* expression and chemotherapeutic resistance, we analyzed the HNC cell line data from the Cancer Cell Line Encyclopedia (CCLE) and Genomics of Drug Sensitivity in Cancer (GDSC) databases. *PIGK* mRNA expression across eight HNC cell lines was compared with the IC_50_ values of six commonly used drugs: cisplatin, 5-fluorouracil (5-FU), docetaxel, paclitaxel, methotrexate, and doxorubicin. A significant positive correlation was observed between *PIGK* levels and docetaxel IC_50_ (*r* = 0.833, *P* = 5 × 10^-3^), while a positive but nonsignificant trend was also noted for paclitaxel (*r* = 0.548, *P* = 8× 10^-2^) (Table 5, Fig. 8A). To further examine the effect of PIGK on HNC cells' response to docetaxel and paclitaxel, we knocked down PIGK in TW1.5 cells. As shown in Fig. 8B-C, TW1.5/shPIGK cells displayed resistance to paclitaxel and docetaxel, suggesting increased sensitivity in a high *PIGK* background. These results support the potential role of PIGK in mediating taxane-based chemotherapy responses in HNC.

## Discussion

PIGK, located on chromosome 1p34.1, encodes a 47 kDa protein that serves as the catalytic subunit of the GPI-T complex 30-32. As a member of the C13 cysteine protease family, PIGK contains a luminal caspase-like domain and a single transmembrane region 30-32.

Recent cryogenic electron microscopy studies revealed a catalytic triad (His164-Cys206-Asn58) within PIGK, supporting a legumain-like transamidation mechanism for GPI attachment 33, 34. A systematic knockout screen in HEK293 cells has further demonstrated that the loss of *PIGK* alone abolished the surface expression of GPI-APs, underscoring its indispensable role in GPI biosynthesis 35. Given the importance of GPI-AP biosynthesis for cellular functions, PIGK dysregulation has been implicated in human diseases. Inherited biallelic loss-of-function mutations in* PIGK* cause GPI-deficiency syndromes characterized by neurodevelopmental phenotypes 36. In cancer, PIGK shows tissue-specific expression changes that are upregulated in ovarian, uterine, and breast cancers but downregulated in bladder, liver, and colorectal cancers 37. Functional assays have further demonstrated that PIGK overexpression enhanced the proliferation and invasion of breast cancer cell lines, suggesting an oncogenic role 37. In colon cancer, a common 3′UTR single-nucleotide polymorphism (rs1048575, C/G or G/G alleles) is associated with reduced PIGK protein expression levels, suggesting post-transcriptional regulatory effects 38. In the current study, we showed that PIGK functions as an oncogene in HNC and that its overexpression is likely driven by copy number gains.

While high PIGK expression is associated with poor prognosis and increased CAF infiltration, it is inversely correlated with TMB. This observation suggests that PIGK-mediated tumor progression occurs through non-mutational mechanisms. In support of this hypothesis, we found that tumors harboring wild-type forms of tumor suppressor genes that are frequently mutated in HNC, including *FAT1*, *CDKN2A*, *CASP8*, and *NOTCH1*, exhibited higher *PIGK* expression levels. A previous study has shown that *NOTCH1* amplification in CAFs suppresses the DNA damage response and promotes stromal cell expansion and tumor progression in cutaneous squamous cell carcinoma 39. Another report has found that *FAT1* upregulation in lung cancer enhanced transforming growth factor-β and epithelial-mesenchymal transition signaling, increased CAF abundance, reduced CD8⁺ T cell infiltration, and was linked to low TMB 40. These findings suggest that PIGK may contribute to oncogenesis through the transcriptional activation of oncogenic programs or remodeling of the tumor stroma rather than through the accumulation of genetic mutations, which is consistent with its association with an immunosuppressive phenotype.

One potential mechanism linking high *PIGK* expression to poor outcomes involves *FAM20C*, which was identified as a candidate co-target of *PIGK* in a two-gene combination analysis. FAM20C is a Golgi-residing kinase that phosphorylates secretory proteins at the Ser-x-Glu/phospho-Ser motif and accounts for most of the extracellular phosphoproteome 41. As a type II transmembrane protein, FAM20C is activated by proteolytic cleavage by site-1 protease, a process that connects its regulation to lipid homeostasis and osteoblast differentiation 42. Functionally, FAM20C is a non-canonical kinase that modifies over 100 substrates involved in ER homeostasis, metabolism, and coagulation 43, 44. Consistent with these broad roles, loss-of-function mutations in *FAM20C* cause several human diseases, including amelogenesis imperfecta, Raine syndrome, and cardiovascular and endocrine disorders 44. Emerging evidence implicates FAM20C in cancer progression. In breast cancer, FAM20C promotes bone metastasis by phosphorylating bone morphogenetic protein 4 and enhancing osteoclastogenesis 45. In glioma, FAM20C drives tumor cell migration and invasion *in vitro*, and the FAM20C antibody significantly reduces tumor size *in vivo* 46. In our study, transcriptomic analysis identified *FAM20C* as an adverse prognostic factor for HNC. Moreover, MCODE-based clustering revealed that PIGK and FAM20C belong to the same protein-protein interaction module enriched in cancer-related pathways. This co-clustering, together with their synergistic prognostic significance, suggests that PIGK and FAM20C may function in related pathways. Mechanistically, prior proteomic and co-immunoprecipitation studies have identified PIGK as part of the FAM20C interactome under ER stress, with both proteins localized in the ER lumen 47. FAM20C phosphorylates protein disulfide isomerase at Ser357, modulating its chaperone function to alleviate ER stress 47. However, it remains to be determined whether FAM20C-driven mechanisms under ER stress influence GPI-anchoring processes and PIGK function, particularly in the context of tumor biology.

In the present study, *in vitro* analyses demonstrated that *PIGK* knockdown reduced chemosensitivity to both paclitaxel and docetaxel, suggesting that high *PIGK* expression may be associated with enhanced responsiveness to taxane-based chemotherapy. Although paclitaxel is less commonly used for HNC, it remains a recognized option in the salvage/recurrent setting. Paclitaxel combined with cetuximab has demonstrated activity in patients who are unfit for or refractory to platinum-based treatment​ 48-50. Docetaxel, on the other hand, is widely used in various treatment settings. For example, in locally advanced unresectable diseases, the addition of docetaxel to cisplatin/5-FU (TPF induction regimen) prolongs patient survival 51, 52. Similarly, docetaxel has been adopted in concurrent chemoradiotherapy settings 53 and in the recurrent setting when combined with methotrexate and cetuximab 54 or as a substitute for 5-FU in the EXTREME regimen (platinum-fluorouracil-cetuximab) 55. Taken together, these results suggest that PIGK expression may serve not only as a prognostic biomarker but also as a predictive marker for taxane responsiveness. Identifying PIGK-high patients may help optimize treatment decisions by favoring taxane-based regimens, potentially improving the therapeutic outcomes in this subgroup. Further mechanistic studies and clinical validation are warranted to establish the role of PIGK in personalized chemotherapy for HNC.

## Conclusion

Taken together, we found that PIGK mRNA and protein levels were significantly elevated in tumor tissues and were consistently associated with adverse clinical features across independent HNC cohorts. Genomic analysis linked *PIGK* upregulation with copy number gain, whereas tumors harboring common tumor suppressor mutations (frequent in HPV-negative HNC) showed lower *PIGK* levels, potentially explaining the differences in* PIGK* levels by HPV status. Protein interaction analyses further connected PIGK to GPI-anchor biosynthesis and membrane-associated signaling, with FAM20C emerging as a potential co-regulated partner. Functional assays confirmed that *PIGK* knockdown inhibited malignant phenotypes, decreased *FAM20C* expression, reduced taxane sensitivity, and weakened fibroblast activation, supporting PIGK as a potential oncogenic driver and predictive biomarker with prognostic and therapeutic relevance in HNC.

## Supplementary Material

Supplementary figures and tables.

## Figures and Tables

**Table 1:** The correlation of *PIGK* level with clinicopathological characteristics in TCGA/ head and neck cancer.

		*PIGK*		
Variable		Mean ± SD	*n* = 490	*P*-value
Age				0.10503
	≥ 60	4.19±1.56	273	
	< 60	4.42±1.65	216	
Sex				0.00574*
	Male	4.41±1.67	360	
	Female	3.96±1.35	130	
Anatomic				0.00013*
	Oral	4.07±1.49	300	
	Non-oral	4.63±1.71	190	
HPV stats				< 0.00001*
	Negative	4.05±1.46	407	
	Positive	5.71±1.74	69	
Alcohol consumption				0.02222*
	Ever/active	4.41±1.64	324	
	No	4.06±1.50	166	
Smoking status				0.28085
	Ever/active	4.23±1.61	305	
	No	4.39±1.60	185	
Differentiation				0.000638*
	Well/moderate	4.10±1.51	353	
	Poor	4.67±1.74	118	
AJCC T				0.01238*
	1+2	4.52±1.64	189	
	3+4	4.14±1.57	301	
AJCC N				0.0423*
	N0	4.13±1.47	222	
	N+	4.43±1.71	264	
Stage				0.3668
	I-III	4.21±1.45	202	
	IV	4.34±1.71	288	

TCGA: The Cancer Genome Atlas Program; SD: standard deviation; HPV: human papillomavirus; AJCC: American Joint Committee of Cancer

**Table 2:** The association of PIGK expression with clinicopathological variables in 100 head and neck cancer patients.

			PIGK			
		Total	Low		High	
Variables		*n* = 100	*n* = 53		*n* = 47	P-value
Gender						1
	Female	6	3		3	
	Male	94	50		44	
Age, years						0.681
	< 60	64	35		29	
	≥ 60	36	18		18	
HPV						0.29
	Positive	17	8		9	
	Negative	83	45		38	
Anatomic location						0.422
	Oral cavity	18	8		10	
	Non-oral cavity	82	45		37	
Alcohol use						0.812
	No	22	11		11	
	Yes	78	42		36	
Betel nut use						0.832
	No	31	17		14	
	Yes	69	36		33	
Smoking status						0.806
	No	20	10		10	
	Yes	80	43		37	
Differentiation						0.136
	1	14	10		4	
	2	80	42		38	
	3	6	1		5	
Tumor thickness						0.082
	> 5.56	59	27		32	
	≤ 5.56	41	26		15	
PNI						0.418
	Present	16	7		9	
	Absent	84	46		38	
T classification						0.011*
	1	31	24		7	
	2	27	11		16	
	3	9	3		6	
	4	33	15		18	
N classification						0.017*
	N0	77	46		31	
	N+	23	7		16	
Stage						0.048*
	I/II	49	31		18	
	III/IV	51	22		29	
Recurrence						0.047*
	No	85	45		36	
	Yes	15	4		11	

HPV: human papillomavirus; PNI: perineural invasion

**Table 3:** Univariate analysis of overall survival in 100 HNC patients.

			Univariate	
Variables	No.	HR	95% CI	*P*-value
Gender				0.841
Male	94	0.841	0.105,6.296	
Female	6	1		
Age				0.322
> 60	36	1.775	0.570,5.524	
≤ 60	64	1		
T classification				
3/4	42	7.372	1.614,33671	0.010*
1/2	58	1		
N classification				0.029*
N+	23	3.591	1.126,11.351	
N0	77	1		
Stage				0.095
III/IV	51	54.087	0.501,5834.7	
I/II	49	1		
PIGK				0.043*
High	47	8.270	1.065,64.216	
Low	53	1		

**Table 4:** The correlation of *FAM20C* levels with clinicopathological features in TCGA/ head and neck cancer.

		*FAM20C*		
Variable		Mean ± SD	*n* = 490	*P*-value
Age				0.14756
	≥ 60	12.09±10.51	273	
	< 60	10.90±6.65	216	
Sex				0.82959
	Male	11.61±9.13	360	
	Female	11.42±8.71	130	
Anatomic				0.02293
	Oral	12.30±9.92	300	
	Non-Oral	10.40±7.23	190	
HPV stats				0.26879
	Negative	11.65±9.05	407	
	Positive	5.71±1.74	69	
Alcohol consumption				0.80042
	Ever/active	11.64±8.85	324	
	No	11.42±9.34	166	
Smoking status				0.93868
	ever/active	11.54±7.51	305	
	No	11.60±11.07	185	
Differentiation				0.19304
	Well/moderate	11.20±8.23	353	
	Poor	12.56±10.87	118	
AJCC T				0.59241
	1+2	11.29±8.92	189	
	3+4	11.73±9.08	301	
AJCC N				0.00453*
	N0	10.33±7.62	222	
	N+	12.66±9.97	264	
Stage				0.00504*
	I-III	10.20±7.01	202	
	IV	12.52±10.09	288	

TCGA: The Cancer Genome Atlas Program; SD: standard deviation; HPV: human papillomavirus; AJCC: American Joint Committee of Cancer

**Table 5:** The association of *PIGK* level and the IC_50_ of chemotherapeutic agents

	*PIGK* level	
Drug	Correlation	*P*-value
Cisplatin	-0.143	0.394
5-fluorouracil	0.095	0.411
Paclitaxel	0.548	0.080
Docetaxel	0.833	0.005*
Methotrexate	-0.310	0.228
Doxorubicin	-0.143	0.368
