# Supplementary material for: Integrative analysis identifies PIGK as an oncogenic glycosylphosphatidylinositol transamidase subunit with prognostic, immunological, and therapeutic relevance in head and neck cancer
Source: Int J Med Sci. 2026 Jan 1;23(1):161–76. doi: 10.7150/ijms.121484 (PMC12701967; doi:10.7150/ijms.121484)
Supplement: Supplementary file 1 — Supplementary figures and tables. [file ijmsv23p0161s1.pdf]

***Integrative analysis identifies PIGK as an oncogenic glycosylphosphatidylinositol transamidase subunit with prognostic, immunological, and therapeutic relevance in head and neck cancer***

**Type of article:** Original Article

**Author:** Yi-Fang Yang, Jia-Bin Liao, Pei-Lun Yu, Chih-Yu Chou, Yu-Hsuan Lin

**Running title:** PIGK as an Oncogenic Driver and Therapeutic Target in Head and Neck Cancer

**Numbers:** Table-5; Figures-8; Supplementary Tables-5, Supplementary Figures-6

**Supplementary information**

| | |
|---|---|
| Supplementary Table 1 | Related to Figure 4 |
| Supplementary Table 2 | Related to Figure 4 |
| Supplementary Table 3 | Related to Figure 5 |
| Supplementary Table 4 | Related to Figure 5 |
| Supplementary Table 5 | Related to Figure 5 |

Supplementary Table 1

Co-expressed transcripts with *PIGK* in TCGA/HNC cohort

Supplementary Table 2

Cancer-related annotations of *PIGK* co-expressed genes identified by IPA

Supplementary Table 3

PIGK-interacting proteins from BioGRID

Supplementary Table 4

PIGK-interacting proteins significantly correlated with *PIGK* expression in TCGA/HNC

Supplementary Table 5

Refined physical interactors of PIGK used for PPI network construction

**Figure S1.**

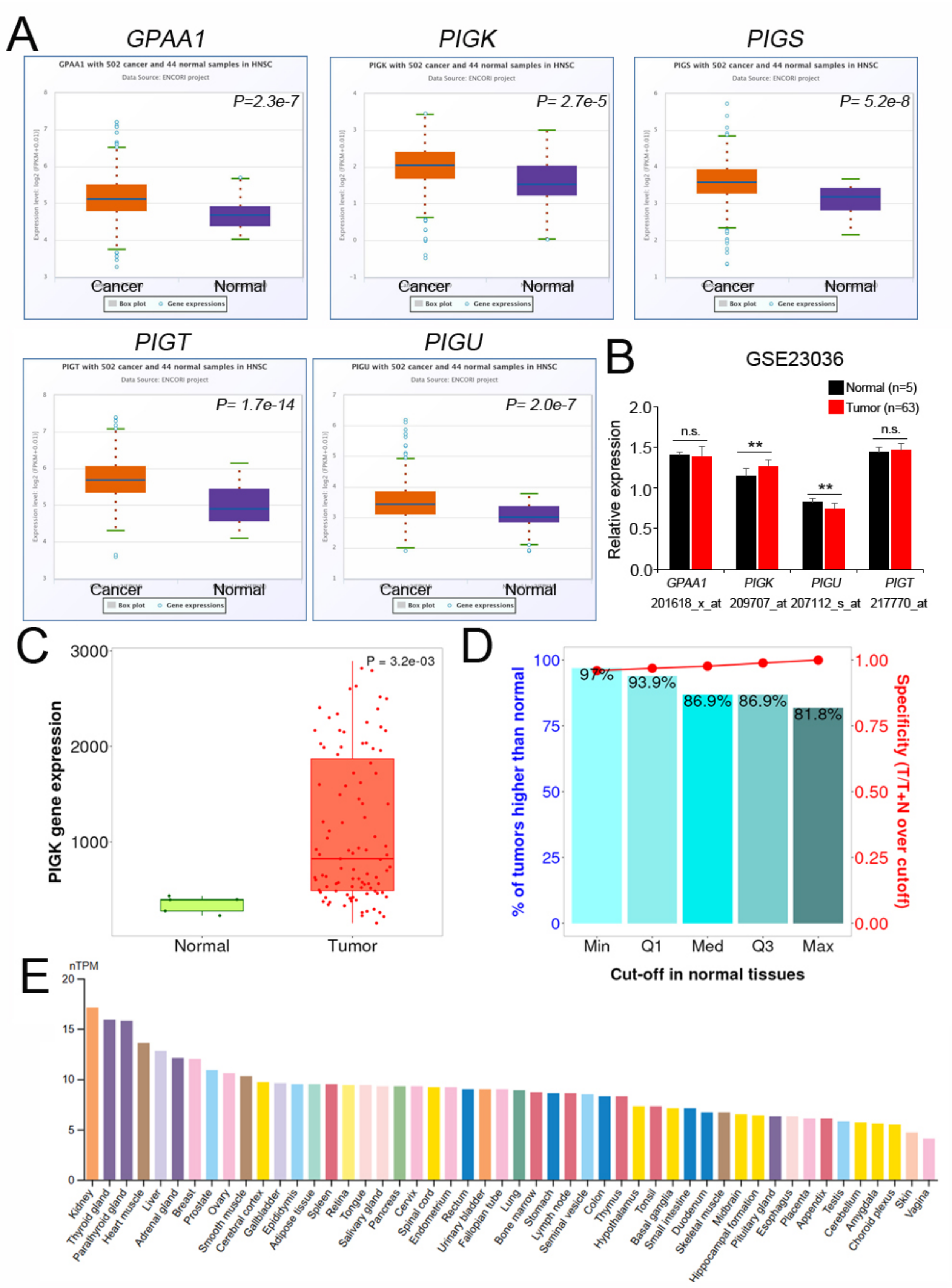
A
GPAA1
PIGK
PIGS
PIGT
PIGU
P=2.3e-7
P= 2.7e-5
P= 5.2e-8
P= 1.7e-14
P= 2.0e-7
Cancer
Normal
B
GSE23036
Normal (n=5)
Tumor (n=63)
Relative expression
n.s.
**
GPAA1
PIGK
PIGU
PIGT
201618_x_at
209707_at
207112_s_at
217770_at
C
PIGK gene expression
P = 3.2e-03
Normal
Tumor
D
% of tumors higher than normal
Specificity (T/T+N over cutoff)
97%
93.9%
86.9%
86.9%
81.8%
Min
Q1
Med
Q3
Max
Cut-off in normal tissues
E
nTPM
Kidney
Thyroid gland
Parathyroid gland
Heart muscle
Liver
Adrenal gland
Breast
Prostate
Ovary
Smooth muscle
Cerebral cortex
Gallbladder
Epididymis
Adipose tissue
Spleen
Retina
Tongue
Salivary gland
Pancreas
Cervix
Spinal cord
Endometrium
Rectum
Urinary bladder
Fallopian tube
Lung
Bone marrow
Stomach
Lymph node
Seminal vesicle
Colon
Thymus
Hypothalamus
Tonsil
Basal ganglia
Small intestine
Duodenum
Skeletal muscle
Midbrain
Hippocampal formation
Pituitary gland
Esophagus
Placenta
Appendix
Testis
Cerebellum
Amygdala
Choroid plexus
Skin
Vagina

**Figure S1. Supplementary transcriptomic and protein-level expression of PIGK in HNC.**

(A) mRNA levels of *GPAA1*, *PIGK*, *PIGS*, *PIGT*, and *PIGU* in patients (*n* = 502) and healthy samples (*n* = 44), based on data from TCGA database (ENCORI). (B) Comparison of *GPAA1*, *PIGK*, *PIGU*, and *PIGT* mRNA levels in normal (*n* = 5) and tumor tissues (*n* = 63) from GSE23036. $^{**}P<0.01$. (C) The differential expression of *PIGK* in tumor and normal tissues based on a gene chip from the TNMplot database. (D) The bar plot shows the ratio of tumor-to-normal expression of *PIGK* across multiple cut-off thresholds. (E) *PIGK* mRNA expression in human tissues based on the Human Protein Atlas (HPA) database.

**Figure S2.**

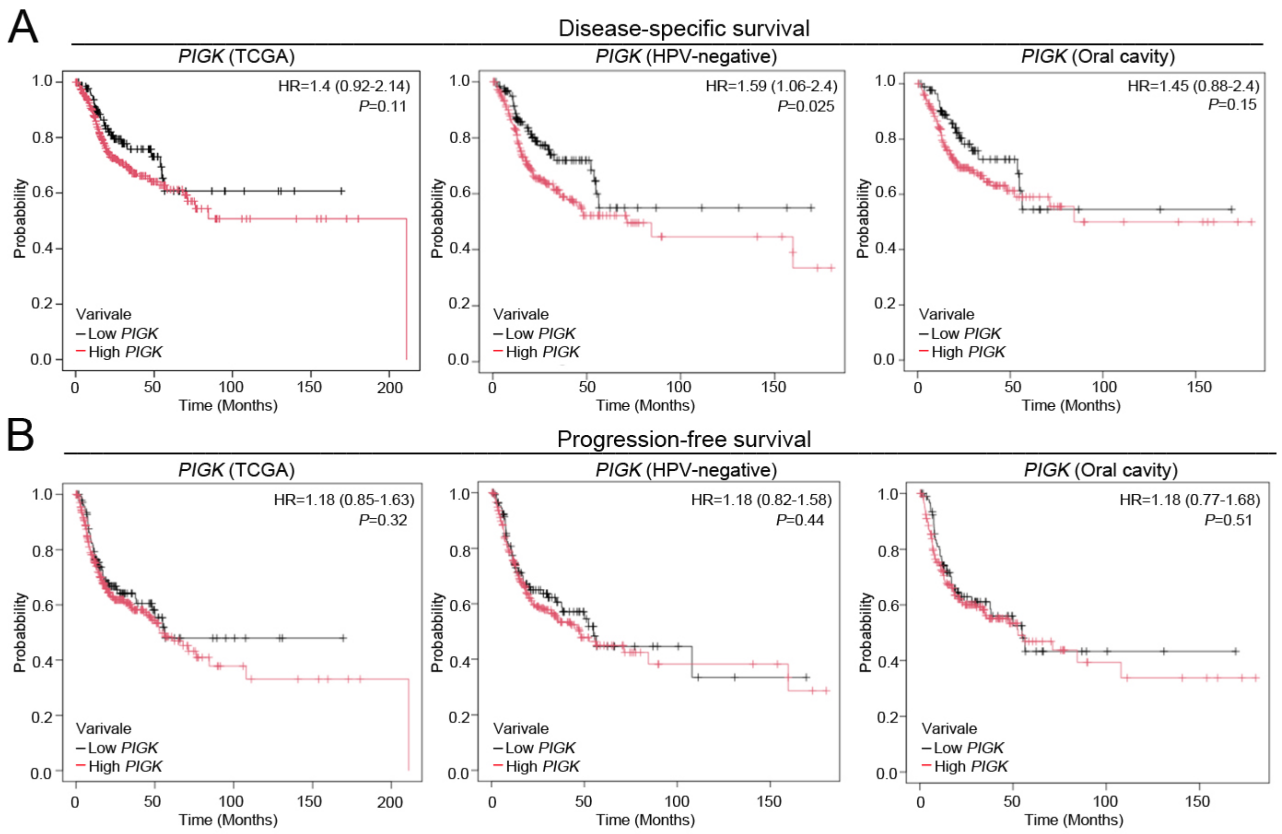


**Figure S2. Kaplan–Meier survival analyses of *PIGK* in the TCGA/HNC cohort.**

(A) Disease-specific survival (DSS) analysis stratified by *PIGK* expression. (B) Progression-free survival (PFS) analysis stratified by *PIGK* expression.

**Figure S3.**

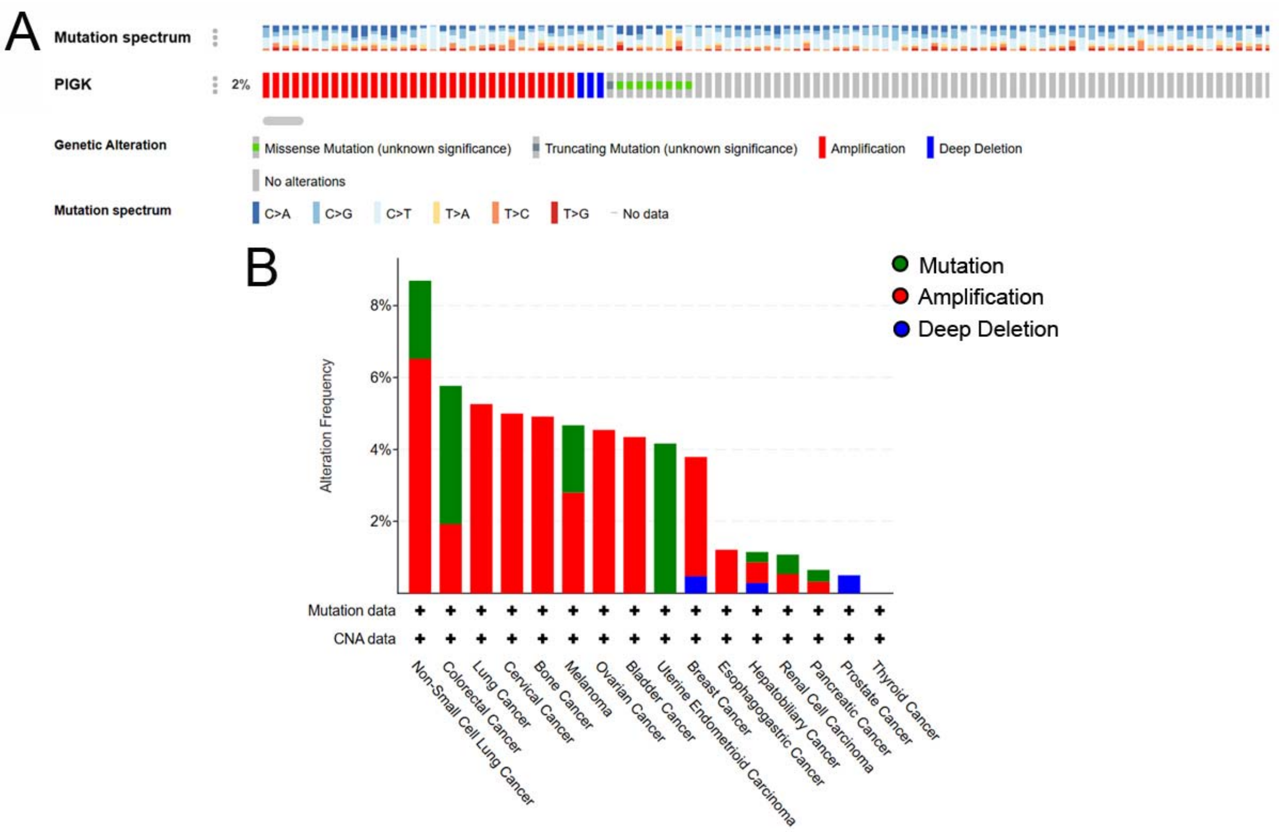


**Figure S3. Genomic alterations of *PIGK* in PanCancer**

(A) Overview of *PIGK* genetic alterations in pan-cancer shown by Oncoprint. (B) Frequency of *PIGK* copy number alterations across various cancer types.

**Figure S4**

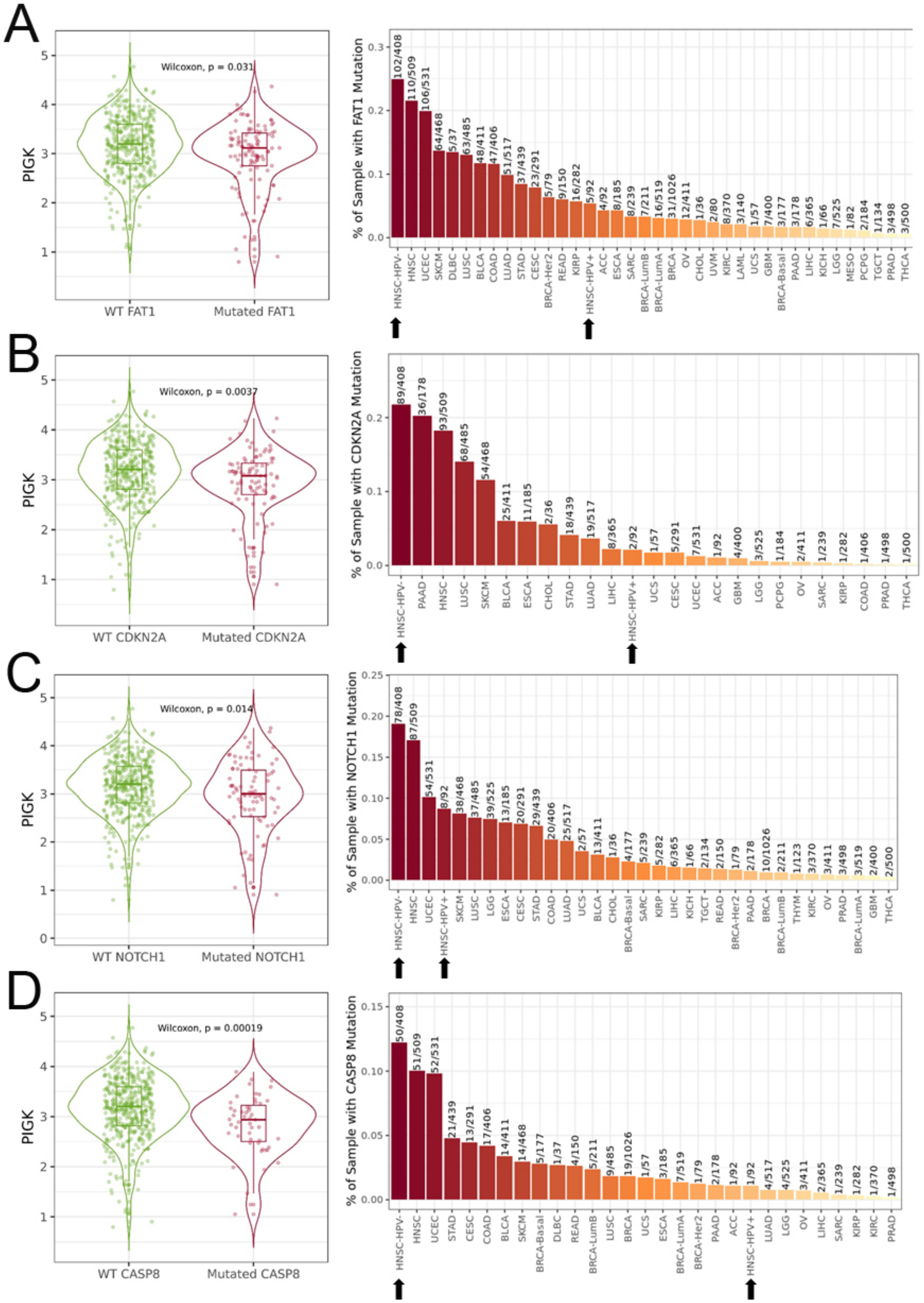


**Figure S4. Comparison of *PIGK* level based on mutation status of top frequently altered genes**

**in the TCGA/HNC cohort.**

Comparison of *PIGK* levels between wild-type and mutant tumors for *FAT1* (A), *CDKN2A* (B), *NOTCH1* (C), and *CASP8* (D). Statistical analysis was performed using the Wilcoxon rank-sum test.

**Figure S5**

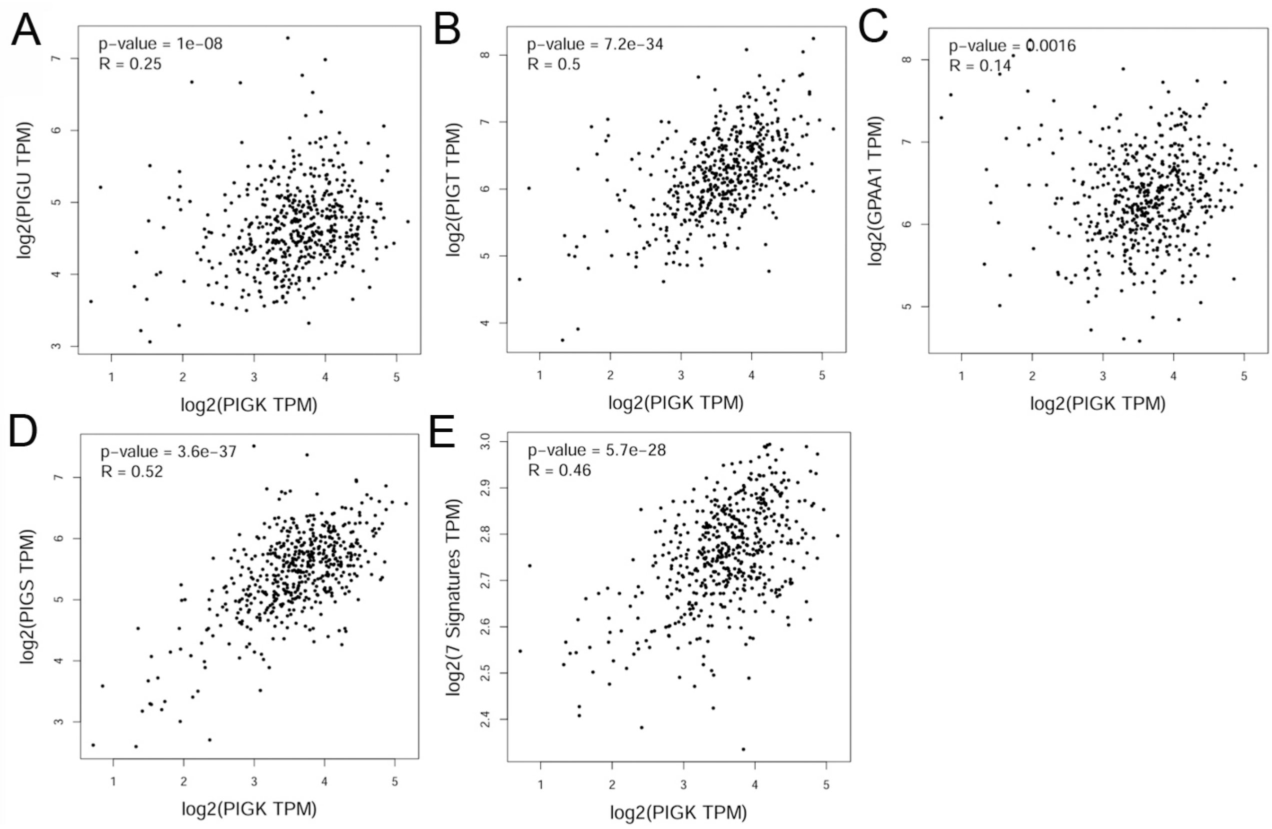


**Figure S5. Correlation of *PIGK* with GPI-T subunits and lipid raft gene signature.**
(A–D) Correlation of *PIGK* expression with other GPI-T subunits, including *PIGU* (A), *PIGT* (B), *GPAA1* (C), and *PIGS* (D). (E) Correlation between *PIGK* expression and a lipid raft–related gene signature (*STOM*, *PHB1*, *FLOT1*, *FLOT2*, *CAV1*, *CAV2*, *CAV3*).

**Figure S6**

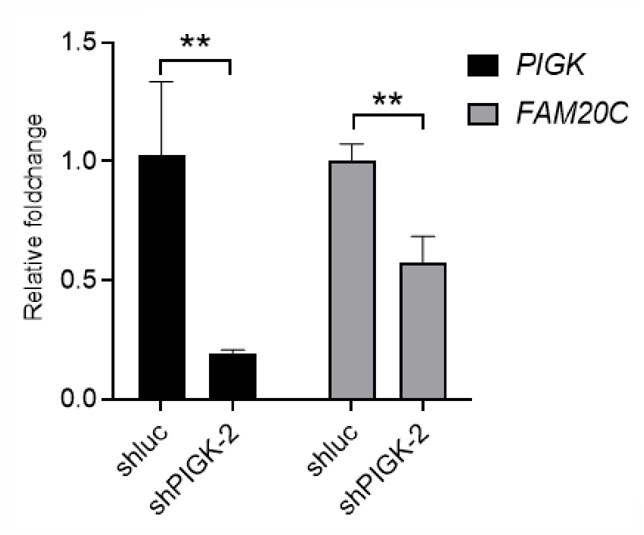


**Figure S6. Knockdown of *PIGK* reduces *FAM20C* expression in TW1.5 cells.** RT-qPCR analysis of *PIGK* and *FAM20C* expression in *PIGK* knockdown cells. Data were presented as the mean ± SD; $^{**}P < 0.01$

**Supplementary Table 1. Co-expressed transcripts with *PIGK* in TCGA/HNC cohort**

| Query | Statistic | *P*-value | FDR (BH) |
|---|---|---|---|
| ABCC9 | 0.338015 | 4.53701E-15 | 8.20446E-14 |
| ABCD3 | 0.4285631 | 3.73219E-24 | 3.78151E-22 |
| ABI3BP | 0.3519888 | 2.72082E-16 | 6.17088E-15 |
| ABL1 | 0.3123103 | 5.60187E-13 | 6.80425E-12 |
| ACAA1 | -0.392882 | 3.10761E-20 | 1.4778E-18 |
| ACADM | 0.4703445 | 2.22303E-29 | 6.59162E-27 |
| ACSL4 | 0.3497919 | 4.27469E-16 | 9.30784E-15 |
| ACTL6A | 0.3245793 | 5.95384E-14 | 8.71171E-13 |
| ACTR2 | 0.3298858 | 2.18673E-14 | 3.49374E-13 |
| ADAM10 | 0.3819668 | 3.98085E-19 | 1.49471E-17 |
| ADAM17 | 0.3132723 | 4.71642E-13 | 5.80214E-12 |
| ADAM19 | 0.3486241 | 5.42724E-16 | 1.16279E-14 |
| ADAM9 | 0.3019282 | 3.44891E-12 | 3.63325E-11 |
| ADAMTS6 | 0.3521072 | 2.65513E-16 | 6.04237E-15 |
| ADAT1 | 0.3348185 | 8.46763E-15 | 1.44444E-13 |
| ADAT3 | -0.381539 | 4.39091E-19 | 1.64256E-17 |
| ADCK2 | -0.333654 | 1.06091E-14 | 1.78259E-13 |
| ADNP | 0.3970864 | 1.1341E-20 | 5.89354E-19 |
| AFAP1 | 0.3807809 | 5.22189E-19 | 1.92133E-17 |
| AGAP3 | -0.439599 | 1.82528E-25 | 2.42125E-23 |
| AGGF1 | 0.37418 | 2.31761E-18 | 7.67324E-17 |
| AGL | 0.3715862 | 4.12416E-18 | 1.28724E-16 |
| AGPAT2 | -0.369634 | 6.34193E-18 | 1.92289E-16 |
| AHCYL1 | 0.3460929 | 9.0742E-16 | 1.86317E-14 |
| AKAP10 | 0.4088854 | 6.19757E-22 | 4.04776E-20 |
| AKAP11 | 0.3492654 | 4.76107E-16 | 1.02781E-14 |
| AKAP2 | 0.4065845 | 1.10249E-21 | 6.9251E-20 |
| AKAP8L | -0.38045 | 5.63156E-19 | 2.06078E-17 |
| AKR1B10 | -0.319334 | 1.57212E-13 | 2.12173E-12 |
| AKR1C1 | -0.306175 | 1.65421E-12 | 1.85402E-11 |
| AKR1C2 | -0.310275 | 8.04462E-13 | 9.57519E-12 |
| AKT3 | 0.3123517 | 5.56061E-13 | 6.7582E-12 |
| ALDH1B1 | 0.3203727 | 1.29904E-13 | 1.78302E-12 |
| ALDH3A1 | -0.301144 | 3.94516E-12 | 4.12103E-11 |
| ALDOA | -0.337674 | 4.85119E-15 | 8.73344E-14 |
| ALG10B | 0.4040361 | 2.07581E-21 | 1.23465E-19 |
| ALG10 | 0.325263 | 5.23876E-14 | 7.73427E-13 |
| ALMS1 | 0.3617834 | 3.47601E-17 | 9.36283E-16 |
| ALS2CR8 | 0.3101549 | 8.21833E-13 | 9.73597E-12 |
| AMN | -0.31648 | 2.64518E-13 | 3.42989E-12 |
| ANAPC11 | -0.322338 | 9.0354E-14 | 1.27936E-12 |
| ANGEL2 | 0.3213841 | 1.07806E-13 | 1.50013E-12 |
| ANKIB1 | 0.3780586 | 9.69465E-19 | 3.39954E-17 |
| ANKRD13C | 0.4422773 | 8.62977E-26 | 1.24878E-23 |
| ANKRD13D | -0.317164 | 2.33633E-13 | 3.06689E-12 |
| ANKRD19 | -0.325803 | 4.73404E-14 | 7.03408E-13 |
| ANKRD26 | 0.3429027 | 1.72301E-15 | 3.34691E-14 |
| ANKRD28 | 0.3163138 | 2.72621E-13 | 3.52589E-12 |

| | | | |
|---|---|---|---|
| ANKRD32 | 0.3871015 | 1.21373E-19 | 5.02513E-18 |
| ANKRD36BP1 | 0.3048232 | 2.09271E-12 | 2.30324E-11 |
| ANKRD39 | -0.323969 | 6.67194E-14 | 9.69909E-13 |
| ANKRD40 | 0.324726 | 5.79279E-14 | 8.48837E-13 |
| ANKRD44 | 0.312599 | 5.3203E-13 | 6.49354E-12 |
| ANKRD9 | -0.345881 | 9.47091E-16 | 1.9387E-14 |
| ANLN | 0.3827611 | 3.31722E-19 | 1.26676E-17 |
| ANO6 | 0.3743271 | 2.24274E-18 | 7.4621E-17 |
| ANO9 | -0.329613 | 2.30327E-14 | 3.66395E-13 |
| ANTXR2 | 0.3598205 | 5.28021E-17 | 1.36319E-15 |
| ANXA5 | 0.3681077 | 8.86074E-18 | 2.60816E-16 |
| ANXA9 | -0.329626 | 2.29764E-14 | 3.66196E-13 |
| AP1S2 | 0.3265099 | 4.14495E-14 | 6.22298E-13 |
| AP2S1 | -0.315787 | 2.99896E-13 | 3.83681E-12 |
| AP3B1 | 0.3481744 | 5.94825E-16 | 1.26513E-14 |
| APAF1 | 0.4695538 | 2.83574E-29 | 8.28652E-27 |
| APOA1BP | -0.333528 | 1.08721E-14 | 1.8192E-13 |
| APPBP2 | 0.3274816 | 3.45092E-14 | 5.27528E-13 |
| APP | 0.3531893 | 2.12242E-16 | 4.9189E-15 |
| APRT | -0.459177 | 6.53075E-28 | 1.44703E-25 |
| ARF5 | -0.384769 | 2.0875E-19 | 8.33469E-18 |
| ARFGAP3 | 0.3457312 | 9.7621E-16 | 1.98821E-14 |
| ARFGEF2 | 0.3108033 | 7.3258E-13 | 8.75869E-12 |
| ARFIP1 | 0.3295095 | 2.34921E-14 | 3.7209E-13 |
| ARHGAP11A | 0.3603812 | 4.68726E-17 | 1.2338E-15 |
| ARHGAP11B | 0.375705 | 1.64751E-18 | 5.58299E-17 |
| ARHGAP18 | 0.347395 | 6.96989E-16 | 1.46848E-14 |
| ARHGAP29 | 0.4302382 | 2.3775E-24 | 2.53638E-22 |
| ARHGAP31 | 0.3262448 | 4.35699E-14 | 6.50741E-13 |
| ARHGAP6 | 0.3182894 | 1.90315E-13 | 2.53289E-12 |
| ARHGDIA | -0.31453 | 3.76268E-13 | 4.72397E-12 |
| ARHGEF5 | -0.32772 | 3.29871E-14 | 5.05794E-13 |
| ARHGEF6 | 0.3479079 | 6.27984E-16 | 1.33005E-14 |
| ARID1B | 0.300584 | 4.34111E-12 | 4.48871E-11 |
| ARL13B | 0.4113465 | 3.33037E-22 | 2.33973E-20 |
| ARL15 | 0.3802126 | 5.94472E-19 | 2.16751E-17 |
| ARL1 | 0.3045981 | 2.17603E-12 | 2.38712E-11 |
| ARL6IP1 | 0.3849262 | 2.01269E-19 | 8.06797E-18 |
| ARL6IP5 | 0.3810652 | 4.89347E-19 | 1.81373E-17 |
| ARL6IP6 | 0.4259674 | 7.46925E-24 | 7.2405E-22 |
| ARL6 | 0.3349273 | 8.29068E-15 | 1.41545E-13 |
| ARMC1 | 0.416739 | 8.38717E-23 | 6.73747E-21 |
| ARMC5 | -0.365725 | 1.48817E-17 | 4.20094E-16 |
| ARNT | 0.3098948 | 8.60552E-13 | 1.01481E-11 |
| ARPC5L | -0.3212 | 1.1153E-13 | 1.54768E-12 |
| ARRDC1 | -0.404874 | 1.68698E-21 | 1.01536E-19 |
| ARSB | 0.401006 | 4.37383E-21 | 2.45653E-19 |
| ARSK | 0.5303049 | 2.94527E-38 | 3.49326E-35 |
| ASAH2B | 0.3173674 | 2.25144E-13 | 2.96705E-12 |
| ASB3 | 0.3825331 | 3.49562E-19 | 1.32485E-17 |
| ASPG | -0.356167 | 1.14104E-16 | 2.77859E-15 |
| ASPM | 0.3124814 | 5.43329E-13 | 6.61543E-12 |

| | | | |
|---|---|---|---|
| ASXL2 | 0.3152488 | 3.30538E-13 | 4.19159E-12 |
| ATAD2 | 0.389682 | 6.6286E-20 | 2.92456E-18 |
| ATAD5 | 0.3517992 | 2.82939E-16 | 6.39564E-15 |
| ATF1 | 0.4672326 | 5.77378E-29 | 1.59475E-26 |
| ATF2 | 0.3834571 | 2.82611E-19 | 1.09372E-17 |
| ATF7 | 0.3537592 | 1.88569E-16 | 4.38536E-15 |
| ATG4C | 0.6308793 | 7.48384E-58 | 5.02989E-54 |
| ATG4D | -0.360337 | 4.73101E-17 | 1.24207E-15 |
| ATG9B | -0.338807 | 3.8831E-15 | 7.13719E-14 |
| ATM | 0.3560724 | 1.16383E-16 | 2.82726E-15 |
| ATP13A3 | 0.3372915 | 5.22853E-15 | 9.3212E-14 |
| ATP2C1 | 0.3474982 | 6.82538E-16 | 1.43954E-14 |
| ATP5D | -0.462789 | 2.21821E-28 | 5.27024E-26 |
| ATP5G1 | -0.325762 | 4.77076E-14 | 7.08342E-13 |
| ATP5H | -0.319487 | 1.52849E-13 | 2.06839E-12 |
| ATP6AP2 | 0.3402376 | 2.92738E-15 | 5.44007E-14 |
| ATP6V0A1 | -0.326138 | 4.44561E-14 | 6.63485E-13 |
| ATP6V0C | -0.416195 | 9.64933E-23 | 7.65982E-21 |
| ATP6V1C1 | 0.3117815 | 6.156E-13 | 7.444E-12 |
| ATP6V1F | -0.354105 | 1.75492E-16 | 4.12887E-15 |
| ATP8B2 | 0.3118648 | 6.06531E-13 | 7.34062E-12 |
| ATRX | 0.3137869 | 4.30057E-13 | 5.34601E-12 |
| ATR | 0.3209526 | 1.16744E-13 | 1.61669E-12 |
| AURKAIP1 | -0.360132 | 4.94203E-17 | 1.28576E-15 |
| AXL | 0.3291962 | 2.49349E-14 | 3.92477E-13 |
| AZIN1 | 0.3306292 | 1.89745E-14 | 3.05822E-13 |
| B3GNT1 | 0.3036497 | 2.56419E-12 | 2.7589E-11 |
| B3GNT8 | -0.32159 | 1.03773E-13 | 1.44901E-12 |
| B3GNT9 | 0.3264202 | 4.21552E-14 | 6.31013E-13 |
| B4GALT6 | 0.3004635 | 4.43137E-12 | 4.57266E-11 |
| BACH2 | 0.3161138 | 2.82683E-13 | 3.642E-12 |
| BAG1 | -0.346887 | 7.72632E-16 | 1.59944E-14 |
| BAIAP2 | -0.414935 | 1.33414E-22 | 1.02283E-20 |
| BARD1 | 0.4051806 | 1.56336E-21 | 9.49459E-20 |
| BBS10 | 0.4833218 | 3.72999E-31 | 1.60016E-28 |
| BBS12 | 0.5018324 | 8.02402E-34 | 5.77815E-31 |
| BBS1 | 0.3694169 | 6.65184E-18 | 2.0048E-16 |
| BBS4 | 0.3637506 | 2.27953E-17 | 6.26187E-16 |
| BBS9 | 0.4919799 | 2.20984E-32 | 1.14249E-29 |
| BBX | 0.4633347 | 1.88245E-28 | 4.62875E-26 |
| BCAP31 | -0.314082 | 4.07829E-13 | 5.08224E-12 |
| BCKDHB | 0.3419057 | 2.10213E-15 | 4.02138E-14 |
| BCOR | 0.3041226 | 2.36287E-12 | 2.5711E-11 |
| BCR | -0.314329 | 3.90151E-13 | 4.877E-12 |
| BDH1 | -0.32276 | 8.35557E-14 | 1.19062E-12 |
| BDP1 | 0.3799421 | 6.32253E-19 | 2.27645E-17 |
| BFAR | 0.3038314 | 2.4849E-12 | 2.68505E-11 |
| BICC1 | 0.3094337 | 9.33622E-13 | 1.09318E-11 |
| BMI1 | 0.3548625 | 1.49871E-16 | 3.556E-15 |
| BMPR2 | 0.3215706 | 1.04154E-13 | 1.45333E-12 |
| BNIPL | -0.326487 | 4.16255E-14 | 6.23547E-13 |
| BOD1L | 0.3673284 | 1.05035E-17 | 3.04286E-16 |

| | | | |
|---|---|---|---|
| BOK | -0.337712 | 4.81491E-15 | 8.68363E-14 |
| BOLA2 | -0.348619 | 5.43247E-16 | 1.16279E-14 |
| BRCA2 | 0.3821207 | 3.84275E-19 | 1.44555E-17 |
| BRI3 | -0.304873 | 2.07452E-12 | 2.28572E-11 |
| BRIP1 | 0.4348427 | 6.79391E-25 | 8.15391E-23 |
| BRWD1 | 0.3013366 | 3.81696E-12 | 3.99799E-11 |
| BVES | 0.386644 | 1.35037E-19 | 5.53406E-18 |
| C10orf116 | -0.307865 | 1.23069E-12 | 1.41554E-11 |
| C10orf25 | 0.3786418 | 8.49543E-19 | 2.99464E-17 |
| C10orf88 | 0.3187204 | 1.759E-13 | 2.34878E-12 |
| C10orf95 | -0.391556 | 4.25796E-20 | 1.96911E-18 |
| C11orf2 | -0.308578 | 1.0856E-12 | 1.25871E-11 |
| C11orf30 | 0.3430405 | 1.67619E-15 | 3.26542E-14 |
| C11orf35 | -0.338818 | 3.87457E-15 | 7.12801E-14 |
| C11orf83 | -0.391609 | 4.20509E-20 | 1.94913E-18 |
| C12orf23 | 0.4357086 | 5.35659E-25 | 6.58566E-23 |
| C12orf44 | -0.361095 | 4.0266E-17 | 1.06827E-15 |
| C14orf106 | 0.4097367 | 5.00242E-22 | 3.31789E-20 |
| C14orf135 | 0.4800437 | 1.06496E-30 | 4.12938E-28 |
| C14orf145 | 0.4735478 | 8.23725E-30 | 2.67884E-27 |
| C14orf2 | -0.322063 | 9.50836E-14 | 1.33881E-12 |
| C14orf45 | 0.4288641 | 3.44233E-24 | 3.52324E-22 |
| C15orf62 | -0.311632 | 6.32267E-13 | 7.62008E-12 |
| C15orf63 | -0.300686 | 4.26619E-12 | 4.42031E-11 |
| C16orf13 | -0.334534 | 8.94891E-15 | 1.51755E-13 |
| C16orf42 | -0.38256 | 3.47413E-19 | 1.31919E-17 |
| C16orf74 | -0.304071 | 2.38388E-12 | 2.58838E-11 |
| C17orf104 | 0.3702679 | 5.51679E-18 | 1.68794E-16 |
| C17orf106 | -0.393406 | 2.74278E-20 | 1.32303E-18 |
| C17orf57 | 0.3613434 | 3.81846E-17 | 1.01959E-15 |
| C17orf71 | 0.3077521 | 1.25528E-12 | 1.44032E-11 |
| C17orf89 | -0.304376 | 2.26134E-12 | 2.47532E-11 |
| C17orf90 | -0.320912 | 1.1761E-13 | 1.62757E-12 |
| C18orf54 | 0.4204969 | 3.16099E-23 | 2.67795E-21 |
| C19orf24 | -0.361272 | 3.87693E-17 | 1.03127E-15 |
| C19orf28 | -0.307143 | 1.39667E-12 | 1.59372E-11 |
| C19orf40 | 0.313065 | 4.89483E-13 | 6.00696E-12 |
| C19orf43 | -0.309965 | 8.49895E-13 | 1.00448E-11 |
| C19orf53 | -0.327017 | 3.76703E-14 | 5.71947E-13 |
| C19orf55 | 0.3060294 | 1.69666E-12 | 1.89911E-11 |
| C19orf60 | -0.350991 | 3.34194E-16 | 7.43747E-15 |
| C19orf73 | -0.331363 | 1.64886E-14 | 2.68113E-13 |
| C1orf109 | 0.4398224 | 1.71532E-25 | 2.30573E-23 |
| C1orf170 | -0.409784 | 4.94325E-22 | 3.28947E-20 |
| C1orf174 | 0.3020979 | 3.34983E-12 | 3.54183E-11 |
| C1orf177 | -0.312692 | 5.23259E-13 | 6.39422E-12 |
| C1orf25 | 0.3281373 | 3.0484E-14 | 4.70635E-13 |
| C1orf26 | 0.3113594 | 6.63639E-13 | 7.97434E-12 |
| C1orf35 | -0.320423 | 1.2871E-13 | 1.76904E-12 |
| C1orf58 | 0.3204292 | 1.2856E-13 | 1.76819E-12 |
| C1orf83 | 0.45865 | 7.63672E-28 | 1.63808E-25 |
| C1orf9 | 0.3034774 | 2.6416E-12 | 2.82895E-11 |

| | | | |
|---|---|---|---|
| C20orf12 | 0.3087776 | 1.04817E-12 | 1.22022E-11 |
| C20orf135 | -0.317998 | 2.00708E-13 | 2.66417E-12 |
| C20orf177 | 0.3039394 | 2.43894E-12 | 2.64389E-11 |
| C21orf70 | -0.405093 | 1.59767E-21 | 9.64488E-20 |
| C3orf17 | 0.3055939 | 1.83039E-12 | 2.03676E-11 |
| C3orf18 | 0.3170497 | 2.38534E-13 | 3.12309E-12 |
| C3orf63 | 0.430569 | 2.17427E-24 | 2.35698E-22 |
| C3orf64 | 0.3570092 | 9.56165E-17 | 2.36845E-15 |
| C4orf29 | 0.334185 | 9.57406E-15 | 1.62084E-13 |
| C4orf42 | 0.3159573 | 2.90808E-13 | 3.73713E-12 |
| C4orf46 | 0.4668392 | 6.50975E-29 | 1.75008E-26 |
| C4orf48 | -0.363956 | 2.18076E-17 | 6.03163E-16 |
| C5orf15 | 0.4657546 | 9.05433E-29 | 2.37094E-26 |
| C5orf22 | 0.3898625 | 6.35273E-20 | 2.82137E-18 |
| C5orf24 | 0.4283627 | 3.93834E-24 | 3.95068E-22 |
| C5orf36 | 0.4372135 | 3.53804E-25 | 4.57292E-23 |
| C5orf41 | 0.3413034 | 2.36967E-15 | 4.49902E-14 |
| C5orf43 | 0.3311045 | 1.73254E-14 | 2.8104E-13 |
| C5orf44 | 0.3239022 | 6.75605E-14 | 9.80016E-13 |
| C5orf51 | 0.3734566 | 2.72314E-18 | 8.87021E-17 |
| C5orf53 | 0.3090403 | 1.00075E-12 | 1.16906E-11 |
| C6orf108 | -0.32732 | 3.55814E-14 | 5.42273E-13 |
| C6orf122 | 0.3296223 | 2.29928E-14 | 3.66196E-13 |
| C6orf132 | -0.348565 | 5.49337E-16 | 1.17458E-14 |
| C6orf150 | 0.3148918 | 3.52531E-13 | 4.44811E-12 |
| C6orf167 | 0.4330469 | 1.10989E-24 | 1.26434E-22 |
| C6orf170 | 0.3191227 | 1.63414E-13 | 2.1925E-12 |
| C6orf182 | 0.4110044 | 3.63179E-22 | 2.53384E-20 |
| C6orf204 | 0.31451 | 3.77631E-13 | 4.73813E-12 |
| C6orf211 | 0.456239 | 1.55689E-27 | 3.20321E-25 |
| C6orf72 | 0.4328673 | 1.16554E-24 | 1.32026E-22 |
| C7orf27 | -0.356385 | 1.09004E-16 | 2.66084E-15 |
| C7orf40 | -0.303454 | 2.65223E-12 | 2.83848E-11 |
| C7orf59 | -0.307808 | 1.24301E-12 | 1.4289E-11 |
| C8orf37 | 0.4245462 | 1.0893E-23 | 9.8952E-22 |
| C8orf83 | 0.3835114 | 2.79095E-19 | 1.08219E-17 |
| C9orf102 | 0.3230039 | 7.98574E-14 | 1.1444E-12 |
| C9orf110 | 0.3385284 | 4.10156E-15 | 7.51133E-14 |
| C9orf129 | 0.3305835 | 1.91412E-14 | 3.08016E-13 |
| C9orf142 | -0.355322 | 1.36168E-16 | 3.26853E-15 |
| C9orf169 | -0.393266 | 2.8358E-20 | 1.36463E-18 |
| C9orf16 | -0.41019 | 4.46158E-22 | 3.01875E-20 |
| C9orf23 | -0.326747 | 3.96375E-14 | 5.98211E-13 |
| C9orf64 | 0.4127457 | 2.33421E-22 | 1.69298E-20 |
| C9orf80 | 0.3428006 | 1.7585E-15 | 3.40929E-14 |
| C9orf86 | -0.365982 | 1.40749E-17 | 3.99146E-16 |
| CACNA2D1 | 0.3087621 | 1.05103E-12 | 1.22285E-11 |
| CALU | 0.3994312 | 6.42351E-21 | 3.48164E-19 |
| CANX | 0.4340893 | 8.35024E-25 | 9.73214E-23 |
| CAPN12 | -0.306742 | 1.49814E-12 | 1.6969E-11 |
| CAPZA1 | 0.3340468 | 9.83369E-15 | 1.65922E-13 |
| CARD8 | 0.3639409 | 2.18805E-17 | 6.0435E-16 |

| | | | |
|---|---|---|---|
| CASC2 | 0.3159199 | 2.92781E-13 | 3.7577E-12 |
| CASC4 | 0.4411687 | 1.17767E-25 | 1.64898E-23 |
| CASC5 | 0.374128 | 2.34469E-18 | 7.71379E-17 |
| CASD1 | 0.4114663 | 3.23081E-22 | 2.27772E-20 |
| CASP8AP2 | 0.4775426 | 2.35318E-30 | 8.9523E-28 |
| CBLB | 0.3011383 | 3.94874E-12 | 4.12103E-11 |
| CBLC | -0.399682 | 6.0428E-21 | 3.293E-19 |
| CBX5 | 0.3011812 | 3.91986E-12 | 4.09726E-11 |
| CCDC110 | 0.3045147 | 2.20773E-12 | 2.41927E-11 |
| CCDC111 | 0.3718426 | 3.89668E-18 | 1.22956E-16 |
| CCDC124 | -0.405541 | 1.42937E-21 | 8.75971E-20 |
| CCDC12 | -0.364847 | 1.79952E-17 | 5.0324E-16 |
| CCDC132 | 0.3608945 | 4.20202E-17 | 1.11188E-15 |
| CCDC137 | -0.340027 | 3.05223E-15 | 5.66687E-14 |
| CCDC14 | 0.3081507 | 1.17041E-12 | 1.34852E-11 |
| CCDC15 | 0.3640329 | 2.14511E-17 | 5.94937E-16 |
| CCDC163P | 0.4670357 | 6.13136E-29 | 1.67063E-26 |
| CCDC18 | 0.393214 | 2.87129E-20 | 1.37515E-18 |
| CCDC21 | 0.3889174 | 7.93423E-20 | 3.41776E-18 |
| CCDC42B | -0.337294 | 5.22604E-15 | 9.3212E-14 |
| CCDC46 | 0.3148113 | 3.57683E-13 | 4.50466E-12 |
| CCDC50 | 0.3878113 | 1.02824E-19 | 4.31924E-18 |
| CCDC51 | -0.307075 | 1.41335E-12 | 1.61094E-11 |
| CCDC64B | -0.39346 | 2.70776E-20 | 1.30927E-18 |
| CCDC75 | 0.3887883 | 8.17843E-20 | 3.49368E-18 |
| CCDC76 | 0.4000351 | 5.54461E-21 | 3.0629E-19 |
| CCDC82 | 0.3152659 | 3.29518E-13 | 4.1813E-12 |
| CCDC85B | -0.337814 | 4.71992E-15 | 8.51994E-14 |
| CCDC88A | 0.4983214 | 2.648E-33 | 1.72231E-30 |
| CCDC99 | 0.4252461 | 9.04782E-24 | 8.56485E-22 |
| CCNE2 | 0.3966714 | 1.25355E-20 | 6.48086E-19 |
| CCNI | 0.3870635 | 1.22453E-19 | 5.04912E-18 |
| CCNT1 | 0.3229132 | 8.12142E-14 | 1.16136E-12 |
| CCPG1 | 0.4297354 | 2.72282E-24 | 2.82991E-22 |
| CCR8 | 0.3065248 | 1.55616E-12 | 1.7529E-11 |
| CCT7 | -0.326978 | 3.79509E-14 | 5.75775E-13 |
| CD164 | 0.4245859 | 1.07793E-23 | 9.87921E-22 |
| CD2BP2 | -0.351539 | 2.98524E-16 | 6.72529E-15 |
| CD302 | 0.3009444 | 4.08187E-12 | 4.2446E-11 |
| CD46 | 0.3345944 | 8.84379E-15 | 1.50431E-13 |
| CDC23 | 0.4018663 | 3.54243E-21 | 2.03493E-19 |
| CDC27 | 0.3988907 | 7.32585E-21 | 3.91807E-19 |
| CDC34 | -0.390364 | 5.64404E-20 | 2.53454E-18 |
| CDC40 | 0.305176 | 1.96835E-12 | 2.18065E-11 |
| CDC5L | 0.3695956 | 6.39586E-18 | 1.93429E-16 |
| CDC73 | 0.361338 | 3.82288E-17 | 1.01959E-15 |
| CDC7 | 0.3759793 | 1.5491E-18 | 5.2672E-17 |
| CDCA8 | 0.3872474 | 1.17308E-19 | 4.86685E-18 |
| CDH2 | 0.3223843 | 8.95902E-14 | 1.27122E-12 |
| CDK14 | 0.3807691 | 5.23598E-19 | 1.92301E-17 |
| CDK17 | 0.4027885 | 2.82395E-21 | 1.65042E-19 |
| CDK2 | 0.4333442 | 1.02347E-24 | 1.17251E-22 |

| | | | |
|---|---|---|---|
| CDK8 | 0.3362011 | 6.47042E-15 | 1.13348E-13 |
| CDR2 | 0.3437919 | 1.44207E-15 | 2.84227E-14 |
| CEBPA | -0.351712 | 2.88047E-16 | 6.5038E-15 |
| CENPE | 0.3070117 | 1.42917E-12 | 1.62437E-11 |
| CENPI | 0.3945335 | 2.09513E-20 | 1.03034E-18 |
| CENPK | 0.374082 | 2.36884E-18 | 7.76633E-17 |
| CENPL | 0.4055293 | 1.43367E-21 | 8.75971E-20 |
| CENPQ | 0.4251216 | 9.35198E-24 | 8.68959E-22 |
| CEP110 | 0.3223691 | 8.98432E-14 | 1.27392E-12 |
| CEP120 | 0.3736177 | 2.62718E-18 | 8.5715E-17 |
| CEP135 | 0.4737658 | 7.69622E-30 | 2.54391E-27 |
| CEP152 | 0.3492097 | 4.8156E-16 | 1.03847E-14 |
| CEP170 | 0.4500109 | 9.55159E-27 | 1.68937E-24 |
| CEP192 | 0.3082205 | 1.15614E-12 | 1.33416E-11 |
| CEP290 | 0.3323294 | 1.36964E-14 | 2.26175E-13 |
| CEP97 | 0.3760519 | 1.52404E-18 | 5.19646E-17 |
| CFD | -0.329022 | 2.57735E-14 | 4.04413E-13 |
| CFL2 | 0.3120583 | 5.85955E-13 | 7.10013E-12 |
| CGGBP1 | 0.3727487 | 3.18741E-18 | 1.025E-16 |
| CHCHD5 | -0.377282 | 1.15544E-18 | 3.99608E-17 |
| CHIC1 | 0.3431696 | 1.63346E-15 | 3.19143E-14 |
| CHMP2A | -0.363469 | 2.42197E-17 | 6.62609E-16 |
| CHMP6 | -0.350214 | 3.92028E-16 | 8.62932E-15 |
| CHM | 0.4328011 | 1.18674E-24 | 1.33678E-22 |
| CHST15 | 0.3309966 | 1.76872E-14 | 2.86447E-13 |
| CHSY1 | 0.3360553 | 6.65704E-15 | 1.15712E-13 |
| CHSY3 | 0.3639038 | 2.20555E-17 | 6.07601E-16 |
| CISD3 | -0.391518 | 4.29628E-20 | 1.98228E-18 |
| CIT | 0.3130474 | 4.91026E-13 | 6.02223E-12 |
| CKAP2L | 0.4151944 | 1.24815E-22 | 9.60548E-21 |
| CLASP2 | 0.3030518 | 2.84285E-12 | 3.03443E-11 |
| CLCC1 | 0.4423276 | 8.50847E-26 | 1.24878E-23 |
| CLDN4 | -0.338584 | 4.05714E-15 | 7.4435E-14 |
| CLGN | 0.308237 | 1.1528E-12 | 1.33127E-11 |
| CLIC3 | -0.399483 | 6.3423E-21 | 3.4469E-19 |
| CLIC4 | 0.4299091 | 2.59826E-24 | 2.72858E-22 |
| CLK3 | -0.356829 | 9.92998E-17 | 2.45667E-15 |
| CLPP | -0.309527 | 9.18302E-13 | 1.07712E-11 |
| CLSPN | 0.4539959 | 3.00521E-27 | 5.94059E-25 |
| CLTA | -0.347384 | 6.985E-16 | 1.47013E-14 |
| CLTB | -0.33721 | 5.31239E-15 | 9.45399E-14 |
| CMTM1 | 0.5733213 | 8.18299E-46 | 2.35705E-42 |
| CMTM6 | 0.3614387 | 3.74157E-17 | 1.00188E-15 |
| CNFN | -0.368577 | 7.99696E-18 | 2.38172E-16 |
| CNN3 | 0.4530486 | 3.96148E-27 | 7.53541E-25 |
| CNOT8 | 0.3011101 | 3.96787E-12 | 4.13672E-11 |
| CNPY4 | 0.3117087 | 6.23638E-13 | 7.52508E-12 |
| CNTLN | 0.305193 | 1.96253E-12 | 2.1754E-11 |
| COASY | -0.330098 | 2.1002E-14 | 3.36884E-13 |
| COG6 | 0.4115142 | 3.1918E-22 | 2.26607E-20 |
| COMMD10 | 0.3457822 | 9.66203E-16 | 1.97182E-14 |
| COMMD2 | 0.3796863 | 6.70148E-19 | 2.40004E-17 |

| | | | |
|---|---|---|---|
| COMTD1 | -0.39259 | 3.33091E-20 | 1.56918E-18 |
| COPA | 0.3797402 | 6.6199E-19 | 2.37504E-17 |
| COPB1 | 0.3194134 | 1.54938E-13 | 2.09384E-12 |
| COPE | -0.336342 | 6.29534E-15 | 1.10665E-13 |
| COX4I1 | -0.395086 | 1.83526E-20 | 9.13914E-19 |
| COX5A | -0.309618 | 9.03697E-13 | 1.06123E-11 |
| COX5B | -0.382887 | 3.22225E-19 | 1.23283E-17 |
| COX6A1 | -0.304457 | 2.22994E-12 | 2.44228E-11 |
| COX6B1 | -0.345801 | 9.6246E-16 | 1.96816E-14 |
| COX6C | -0.330811 | 1.83257E-14 | 2.96074E-13 |
| COX7B | -0.301734 | 3.56551E-12 | 3.75021E-11 |
| COX8A | -0.335018 | 8.14665E-15 | 1.3944E-13 |
| CP110 | 0.4399604 | 1.65057E-25 | 2.26398E-23 |
| CPD | 0.3935088 | 2.67642E-20 | 1.29723E-18 |
| CPEB1 | 0.3423844 | 1.91084E-15 | 3.66935E-14 |
| CPE | 0.3204557 | 1.27936E-13 | 1.7608E-12 |
| CRB3 | -0.408522 | 6.78959E-22 | 4.37375E-20 |
| CREB1 | 0.3445847 | 1.22987E-15 | 2.4601E-14 |
| CRY1 | 0.3371737 | 5.35055E-15 | 9.5135E-14 |
| CRYBB3 | -0.306771 | 1.49052E-12 | 1.69124E-11 |
| CRYBG3 | 0.3050071 | 2.02697E-12 | 2.23821E-11 |
| CSF1 | 0.3394001 | 3.45432E-15 | 6.37231E-14 |
| CSGALNACT2 | 0.4036257 | 2.29731E-21 | 1.36237E-19 |
| CSNK1G3 | 0.3926014 | 3.32214E-20 | 1.56872E-18 |
| CSTA | -0.331785 | 1.52078E-14 | 2.495E-13 |
| CSTB | -0.341147 | 2.44422E-15 | 4.63184E-14 |
| CSTF2T | 0.4302738 | 2.35472E-24 | 2.52544E-22 |
| CTBS | 0.5327744 | 1.15956E-38 | 1.55868E-35 |
| CTDSPL2 | 0.448323 | 1.55171E-26 | 2.52315E-24 |
| CTU1 | -0.331549 | 1.59101E-14 | 2.59754E-13 |
| CTXN1 | -0.301504 | 3.70894E-12 | 3.89294E-11 |
| CXorf23 | 0.3335624 | 1.07992E-14 | 1.80851E-13 |
| CYB5R4 | 0.3123934 | 5.51943E-13 | 6.71221E-12 |
| CYB5RL | 0.3160588 | 2.85512E-13 | 3.67376E-12 |
| CYHR1 | -0.35519 | 1.3998E-16 | 3.34685E-15 |
| CYP2U1 | 0.3826489 | 3.40382E-19 | 1.29493E-17 |
| CYP4F22 | -0.316355 | 2.70607E-13 | 3.50434E-12 |
| CYSLTR1 | 0.3175934 | 2.16068E-13 | 2.85865E-12 |
| DAAM2 | 0.3122936 | 5.61861E-13 | 6.82047E-12 |
| DAB2 | 0.3266641 | 4.02631E-14 | 6.06292E-13 |
| DAP | 0.3168661 | 2.46625E-13 | 3.21857E-12 |
| DAZAP1 | -0.347546 | 6.75937E-16 | 1.42861E-14 |
| DBR1 | 0.329254 | 2.46624E-14 | 3.88794E-13 |
| DBT | 0.4719429 | 1.35632E-29 | 4.14357E-27 |
| DCBLD2 | 0.3752104 | 1.84071E-18 | 6.1857E-17 |
| DCK | 0.4304084 | 2.2707E-24 | 2.44835E-22 |
| DCLRE1A | 0.382388 | 3.61407E-19 | 1.36718E-17 |
| DCLRE1B | 0.4473644 | 2.04156E-26 | 3.24126E-24 |
| DCP2 | 0.3005783 | 4.34539E-12 | 4.49083E-11 |
| DCUN1D4 | 0.377503 | 1.09918E-18 | 3.80804E-17 |
| DCXR | -0.34753 | 6.78165E-16 | 1.43182E-14 |
| DDI2 | 0.3042468 | 2.31258E-12 | 2.52593E-11 |

| | | | |
|---|---|---|---|
| DDOST | 0.3409577 | 2.53806E-15 | 4.77824E-14 |
| DDRGK1 | -0.325124 | 5.37717E-14 | 7.90809E-13 |
| DDT | -0.320299 | 1.31677E-13 | 1.80613E-12 |
| DDX20 | 0.4345277 | 7.40628E-25 | 8.78428E-23 |
| DDX54 | -0.350765 | 3.50103E-16 | 7.74877E-15 |
| DEFB1 | -0.301468 | 3.73203E-12 | 3.9131E-11 |
| DEK | 0.3406417 | 2.7022E-15 | 5.05128E-14 |
| DEM1 | 0.4099385 | 4.75419E-22 | 3.19469E-20 |
| DENND5B | 0.3566847 | 1.02359E-16 | 2.51079E-15 |
| DEPDC1 | 0.4399022 | 1.67758E-25 | 2.27176E-23 |
| DET1 | 0.3272601 | 3.59835E-14 | 5.47987E-13 |
| DHFRL1 | 0.3394372 | 3.42913E-15 | 6.33164E-14 |
| DHRS4L1 | -0.323803 | 6.88212E-14 | 9.97121E-13 |
| DHRS4L2 | -0.410217 | 4.43154E-22 | 3.00852E-20 |
| DHRS4 | -0.399767 | 5.91922E-21 | 3.25202E-19 |
| DHX33 | 0.3361359 | 6.55331E-15 | 1.14402E-13 |
| DHX40 | 0.3889678 | 7.84084E-20 | 3.3926E-18 |
| DHX57 | 0.3000226 | 4.77731E-12 | 4.90703E-11 |
| DISP1 | 0.3695936 | 6.39869E-18 | 1.93429E-16 |
| DKFZp761E198 | -0.323316 | 7.53542E-14 | 1.08604E-12 |
| DLEU2L | 0.3065858 | 1.53966E-12 | 1.73671E-11 |
| DMGDH | 0.3020815 | 3.35928E-12 | 3.54996E-11 |
| DMKN | -0.334048 | 9.83119E-15 | 1.65922E-13 |
| DMXL1 | 0.3192596 | 1.59368E-13 | 2.14939E-12 |
| DNA2 | 0.3243807 | 6.17892E-14 | 9.02794E-13 |
| DNAJC10 | 0.4144579 | 1.50755E-22 | 1.15139E-20 |
| DNAJC13 | 0.3904163 | 5.57506E-20 | 2.51476E-18 |
| DNAJC14 | 0.3882505 | 9.27766E-20 | 3.9135E-18 |
| DNAJC16 | 0.3510245 | 3.31905E-16 | 7.3947E-15 |
| DNAJC18 | 0.3535421 | 1.97262E-16 | 4.57697E-15 |
| DNAJC24 | 0.3298408 | 2.20554E-14 | 3.52101E-13 |
| DNAJC30 | -0.32611 | 4.46869E-14 | 6.66437E-13 |
| DNAJC7 | -0.316265 | 2.75031E-13 | 3.5525E-12 |
| DNAL1 | 0.4649561 | 1.15353E-28 | 2.89264E-26 |
| DNLZ | -0.312959 | 4.98895E-13 | 6.11503E-12 |
| DNMT1 | 0.3117766 | 6.16131E-13 | 7.444E-12 |
| DOCK10 | 0.3213006 | 1.09482E-13 | 1.5224E-12 |
| DOCK1 | 0.3919207 | 3.90512E-20 | 1.82266E-18 |
| DOCK7 | 0.4032265 | 2.53508E-21 | 1.49458E-19 |
| DOHH | -0.302042 | 3.38228E-12 | 3.57052E-11 |
| DOPEY1 | 0.3346671 | 8.72006E-15 | 1.48499E-13 |
| DPH3B | 0.3237623 | 6.93453E-14 | 1.00302E-12 |
| DPM3 | -0.323794 | 6.89387E-14 | 9.97855E-13 |
| DPP8 | 0.3240019 | 6.6316E-14 | 9.6474E-13 |
| DPY19L1 | 0.407313 | 9.1914E-22 | 5.84625E-20 |
| DPY19L3 | 0.4448139 | 4.2184E-26 | 6.4928E-24 |
| DPY19L4 | 0.5030293 | 5.32406E-34 | 4.0485E-31 |
| DPYD | 0.4057481 | 1.35781E-21 | 8.37231E-20 |
| DR1 | 0.5167063 | 4.36224E-36 | 4.18837E-33 |
| DSG2 | 0.3571391 | 9.30394E-17 | 2.31314E-15 |
| DUS1L | -0.441385 | 1.10854E-25 | 1.57406E-23 |
| DUS2L | -0.304221 | 2.32294E-12 | 2.53313E-11 |

| | | | |
|---|---|---|---|
| DVL1 | -0.307615 | 1.28577E-12 | 1.47301E-11 |
| DYNC2H1 | 0.3099569 | 8.51153E-13 | 1.00538E-11 |
| DYNLL1 | -0.302101 | 3.34804E-12 | 3.54179E-11 |
| DYRK1A | 0.3864798 | 1.40303E-19 | 5.72659E-18 |
| DZIP3 | 0.3560654 | 1.16554E-16 | 2.82802E-15 |
| E2F7 | 0.3989942 | 7.14394E-21 | 3.83094E-19 |
| E2F8 | 0.3079238 | 1.21801E-12 | 1.40176E-11 |
| E4F1 | -0.331977 | 1.46553E-14 | 2.41418E-13 |
| EBPL | -0.400605 | 4.82411E-21 | 2.7019E-19 |
| ECM2 | 0.3040883 | 2.37691E-12 | 2.5822E-11 |
| ECT2 | 0.3709983 | 4.69631E-18 | 1.44949E-16 |
| EDEM3 | 0.391281 | 4.54442E-20 | 2.08722E-18 |
| EDF1 | -0.365368 | 1.60777E-17 | 4.5087E-16 |
| EEF1D | -0.32888 | 2.64777E-14 | 4.14174E-13 |
| EEF1G | -0.361318 | 3.83927E-17 | 1.02261E-15 |
| EFCAB7 | 0.506204 | 1.77953E-34 | 1.49503E-31 |
| EFNA3 | -0.336874 | 5.67389E-15 | 1.00618E-13 |
| EFR3A | 0.3684604 | 8.20304E-18 | 2.42519E-16 |
| EGLN2 | -0.303855 | 2.47469E-12 | 2.67689E-11 |
| EHHADH | 0.3232306 | 7.65608E-14 | 1.10107E-12 |
| EID1 | 0.3358289 | 6.95726E-15 | 1.20722E-13 |
| EIF2AK3 | 0.3376327 | 4.89038E-15 | 8.79614E-14 |
| EIF2B4 | -0.408984 | 6.04565E-22 | 3.97063E-20 |
| EIF3B | -0.36924 | 6.91462E-18 | 2.07469E-16 |
| EIF3CL | -0.36184 | 3.4344E-17 | 9.28256E-16 |
| EIF3F | -0.301112 | 3.96656E-12 | 4.13672E-11 |
| EIF3G | -0.328523 | 2.83334E-14 | 4.41489E-13 |
| EIF6 | -0.333227 | 1.15217E-14 | 1.92311E-13 |
| ELF2 | 0.3884434 | 8.86755E-20 | 3.76413E-18 |
| ELK3 | 0.3352604 | 7.77112E-15 | 1.33694E-13 |
| ELMO2 | 0.3086317 | 1.07545E-12 | 1.24766E-11 |
| ELMO3 | -0.30018 | 4.6508E-12 | 4.78928E-11 |
| ELOVL5 | 0.3972009 | 1.10316E-20 | 5.76244E-19 |
| ENDOG | -0.44226 | 8.67079E-26 | 1.24878E-23 |
| ENPP4 | 0.3174081 | 2.23484E-13 | 2.94903E-12 |
| ENSA | -0.302754 | 2.99231E-12 | 3.18385E-11 |
| EPB41L2 | 0.3497037 | 4.35267E-16 | 9.46741E-15 |
| EPC1 | 0.3613713 | 3.79583E-17 | 1.01506E-15 |
| EPC2 | 0.3731327 | 2.92666E-18 | 9.50244E-17 |
| EPHA3 | 0.3527085 | 2.3447E-16 | 5.39066E-15 |
| EPN1 | -0.356712 | 1.01769E-16 | 2.50554E-15 |
| EPR1 | 0.3048389 | 2.08699E-12 | 2.2982E-11 |
| EPS15 | 0.3559387 | 1.1969E-16 | 2.89712E-15 |
| EPS8L1 | -0.384467 | 2.23819E-19 | 8.84876E-18 |
| EPS8L2 | -0.302169 | 3.30933E-12 | 3.50636E-11 |
| ERAP1 | 0.3691643 | 7.03085E-18 | 2.10331E-16 |
| ERBB2IP | 0.428474 | 3.82247E-24 | 3.85362E-22 |
| ERCC1 | -0.300349 | 4.51876E-12 | 4.65807E-11 |
| ERCC4 | 0.4077437 | 8.25255E-22 | 5.28242E-20 |
| ERCC8 | 0.4164849 | 8.95539E-23 | 7.13706E-21 |
| ERLEC1 | 0.3692595 | 6.88555E-18 | 2.06905E-16 |
| ESRRA | -0.331878 | 1.49381E-14 | 2.45675E-13 |

| | | | |
|---|---|---|---|
| ETS1 | 0.3767074 | 1.31515E-18 | 4.53287E-17 |
| EVI5 | 0.3963946 | 1.34003E-20 | 6.84026E-19 |
| EVPLL | -0.303384 | 2.68434E-12 | 2.86979E-11 |
| EVPL | -0.399133 | 6.90621E-21 | 3.72005E-19 |
| EXD3 | -0.401539 | 3.83833E-21 | 2.18354E-19 |
| EXOC5 | 0.3399009 | 3.129E-15 | 5.79872E-14 |
| EXOSC4 | -0.423206 | 1.55244E-23 | 1.36689E-21 |
| EXOSC7 | -0.316233 | 2.76661E-13 | 3.56898E-12 |
| EXTL2 | 0.4767266 | 3.04358E-30 | 1.11578E-27 |
| F12 | -0.414159 | 1.62731E-22 | 1.2289E-20 |
| F2R | 0.3550351 | 1.44572E-16 | 3.44515E-15 |
| F8 | 0.3699139 | 5.96365E-18 | 1.81092E-16 |
| FAF2 | 0.336321 | 6.32085E-15 | 1.11017E-13 |
| FAM100A | -0.326937 | 3.82422E-14 | 5.78888E-13 |
| FAM100B | -0.384808 | 2.0687E-19 | 8.27603E-18 |
| FAM102B | 0.397114 | 1.12657E-20 | 5.86954E-19 |
| FAM105A | 0.3254765 | 5.03323E-14 | 7.45118E-13 |
| FAM107B | 0.3294517 | 2.3752E-14 | 3.75911E-13 |
| FAM108A1 | -0.371608 | 4.10464E-18 | 1.28313E-16 |
| FAM110A | -0.335588 | 7.29186E-15 | 1.26202E-13 |
| FAM111B | 0.3434324 | 1.54976E-15 | 3.0456E-14 |
| FAM114A1 | 0.3197025 | 1.46933E-13 | 1.99582E-12 |
| FAM114A2 | 0.3708492 | 4.85339E-18 | 1.49175E-16 |
| FAM125A | -0.323802 | 6.88386E-14 | 9.97121E-13 |
| FAM128A | -0.342448 | 1.8869E-15 | 3.63377E-14 |
| FAM128B | -0.389655 | 6.67151E-20 | 2.93707E-18 |
| FAM129B | -0.338221 | 4.35717E-15 | 7.93618E-14 |
| FAM136A | -0.317042 | 2.38875E-13 | 3.12552E-12 |
| FAM13B | 0.3590847 | 6.1716E-17 | 1.57716E-15 |
| FAM13C | 0.326739 | 3.9699E-14 | 5.98692E-13 |
| FAM158A | -0.306027 | 1.69727E-12 | 1.89911E-11 |
| FAM161A | 0.4190847 | 4.56785E-23 | 3.75925E-21 |
| FAM161B | 0.4883922 | 7.19785E-32 | 3.37512E-29 |
| FAM164A | 0.3309615 | 1.78063E-14 | 2.87914E-13 |
| FAM178A | 0.4085255 | 6.78395E-22 | 4.37375E-20 |
| FAM179B | 0.3525504 | 2.42267E-16 | 5.55095E-15 |
| FAM195A | -0.332128 | 1.42383E-14 | 2.3474E-13 |
| FAM198B | 0.3210278 | 1.15135E-13 | 1.59661E-12 |
| FAM21A | 0.3326122 | 1.29709E-14 | 2.15625E-13 |
| FAM25A | -0.40321 | 2.54518E-21 | 1.49617E-19 |
| FAM25B | -0.368056 | 8.96084E-18 | 2.63378E-16 |
| FAM38B | 0.3806815 | 5.34164E-19 | 1.95825E-17 |
| FAM3A | -0.306731 | 1.50117E-12 | 1.69854E-11 |
| FAM70A | 0.3086676 | 1.06869E-12 | 1.24124E-11 |
| FAM73A | 0.5336781 | 8.22818E-39 | 1.18503E-35 |
| FAM76A | 0.3567904 | 1.00114E-16 | 2.47075E-15 |
| FAM83F | -0.363534 | 2.38826E-17 | 6.54272E-16 |
| FAM83H | -0.358783 | 6.57838E-17 | 1.66843E-15 |
| FAM91A1 | 0.320128 | 1.35885E-13 | 1.86004E-12 |
| FAM96B | -0.316968 | 2.42121E-13 | 3.16469E-12 |
| FAM98B | 0.3336798 | 1.0557E-14 | 1.77532E-13 |
| FANCB | 0.3268898 | 3.85872E-14 | 5.83672E-13 |

| | | | |
|---|---|---|---|
| FANCM | 0.4236106 | 1.3951E-23 | 1.23918E-21 |
| FAR2 | 0.4070065 | 9.92306E-22 | 6.27206E-20 |
| FASTKD3 | 0.3398361 | 3.16933E-15 | 5.86807E-14 |
| FASTK | -0.402525 | 3.01296E-21 | 1.75073E-19 |
| FAS | 0.3852425 | 1.87042E-19 | 7.52762E-18 |
| FAT4 | 0.3538754 | 1.84069E-16 | 4.30055E-15 |
| FAU | -0.312473 | 5.44119E-13 | 6.62105E-12 |
| FBRS | -0.315855 | 2.96256E-13 | 3.79746E-12 |
| FBXL15 | -0.388987 | 7.80524E-20 | 3.38445E-18 |
| FBXL19 | -0.365407 | 1.59431E-17 | 4.48341E-16 |
| FBXL8 | -0.314967 | 3.4777E-13 | 4.39354E-12 |
| FBXO21 | 0.3169662 | 2.42182E-13 | 3.16469E-12 |
| FBXO5 | 0.451121 | 6.93176E-27 | 1.28225E-24 |
| FBXW4 | -0.337357 | 5.16159E-15 | 9.22863E-14 |
| FCF1 | 0.3255734 | 4.94256E-14 | 7.32771E-13 |
| FDXR | -0.449801 | 1.01486E-26 | 1.76279E-24 |
| FGD6 | 0.3407392 | 2.65051E-15 | 4.96674E-14 |
| FICD | 0.3083839 | 1.1234E-12 | 1.29954E-11 |
| FIGNL1 | 0.3472725 | 7.14546E-16 | 1.49765E-14 |
| FIGN | 0.3009522 | 4.0765E-12 | 4.2412E-11 |
| FILIP1L | 0.3133746 | 4.63074E-13 | 5.71417E-12 |
| FKBP14 | 0.3571025 | 9.37584E-17 | 2.32814E-15 |
| FKBP2 | -0.322122 | 9.40545E-14 | 1.32524E-12 |
| FKBP7 | 0.3097389 | 8.84613E-13 | 1.04063E-11 |
| FKBP8 | -0.331405 | 1.63567E-14 | 2.66397E-13 |
| FKTN | 0.4015327 | 3.84444E-21 | 2.18354E-19 |
| FLJ23867 | -0.314896 | 3.52276E-13 | 4.44768E-12 |
| FMNL3 | 0.3910931 | 4.75099E-20 | 2.1722E-18 |
| FNBP1L | 0.331027 | 1.75843E-14 | 2.8501E-13 |
| FNDC3A | 0.4458021 | 3.18675E-26 | 4.98097E-24 |
| FNDC3B | 0.4864295 | 1.3652E-31 | 6.11699E-29 |
| FNIP1 | 0.343837 | 1.42908E-15 | 2.82128E-14 |
| FOXH1 | -0.331674 | 1.55352E-14 | 2.53838E-13 |
| FOXJ3 | 0.399412 | 6.4536E-21 | 3.48857E-19 |
| FPGT | 0.4073308 | 9.15069E-22 | 5.83878E-20 |
| FRRS1 | 0.3617807 | 3.47803E-17 | 9.36283E-16 |
| FRS2 | 0.3546177 | 1.57721E-16 | 3.72381E-15 |
| FRYL | 0.389735 | 6.54633E-20 | 2.8946E-18 |
| FSD1L | 0.3051608 | 1.97356E-12 | 2.18457E-11 |
| FSIP1 | 0.3229676 | 8.0397E-14 | 1.15131E-12 |
| FTO | 0.4117486 | 3.00754E-22 | 2.15039E-20 |
| FUBP1 | 0.4308596 | 2.00998E-24 | 2.19066E-22 |
| FUT4 | 0.3208596 | 1.18762E-13 | 1.64239E-12 |
| FUT8 | 0.4961289 | 5.54221E-33 | 3.28669E-30 |
| FXYD3 | -0.30596 | 1.71743E-12 | 1.92036E-11 |
| G2E3 | 0.3197802 | 1.44853E-13 | 1.9721E-12 |
| G3BP1 | 0.31594 | 2.91718E-13 | 3.74644E-12 |
| G3BP2 | 0.3839313 | 2.53333E-19 | 9.87999E-18 |
| G6PD | -0.358265 | 7.3394E-17 | 1.8498E-15 |
| GABPA | 0.3326858 | 1.27884E-14 | 2.13102E-13 |
| GADD45GIP1 | -0.385211 | 1.88425E-19 | 7.56815E-18 |
| GALC | 0.4106861 | 3.93636E-22 | 2.70884E-20 |

| | | | |
|---|---|---|---|
| GALK1 | -0.324033 | 6.59307E-14 | 9.59827E-13 |
| GALNT10 | 0.3723849 | 3.45541E-18 | 1.1024E-16 |
| GALNT4 | 0.304931 | 2.05393E-12 | 2.26569E-11 |
| GANAB | 0.4017652 | 3.63143E-21 | 2.08013E-19 |
| GAS2L3 | 0.4533461 | 3.63261E-27 | 6.97565E-25 |
| GAST | -0.310254 | 8.07566E-13 | 9.60079E-12 |
| GCA | 0.3070318 | 1.42414E-12 | 1.62025E-11 |
| GCC2 | 0.3500707 | 4.03731E-16 | 8.86757E-15 |
| GDPD3 | -0.338249 | 4.33326E-15 | 7.89977E-14 |
| GET4 | -0.404122 | 2.03224E-21 | 1.21231E-19 |
| GFOD2 | -0.317325 | 2.26908E-13 | 2.98834E-12 |
| GGA1 | -0.347125 | 7.36189E-16 | 1.53401E-14 |
| GIN1 | 0.3229273 | 8.10008E-14 | 1.15913E-12 |
| GIPC1 | -0.491711 | 2.41586E-32 | 1.21778E-29 |
| GIT2 | 0.4243903 | 1.13523E-23 | 1.02186E-21 |
| GLB1 | 0.3723265 | 3.50047E-18 | 1.11501E-16 |
| GLI4 | -0.317088 | 2.36886E-13 | 3.10353E-12 |
| GLTPD1 | -0.359099 | 6.15318E-17 | 1.57445E-15 |
| GLTP | -0.352428 | 2.48489E-16 | 5.68059E-15 |
| GLTSCR2 | -0.313322 | 4.67494E-13 | 5.76166E-12 |
| GMCL1 | 0.3242864 | 6.28879E-14 | 9.16854E-13 |
| GMFB | 0.3274142 | 3.49512E-14 | 5.33476E-13 |
| GNB2 | -0.37931 | 7.3008E-19 | 2.59622E-17 |
| GNB4 | 0.4225562 | 1.8421E-23 | 1.60789E-21 |
| GNG12 | 0.3803264 | 5.79241E-19 | 2.1158E-17 |
| GNG2 | 0.3292354 | 2.47497E-14 | 3.89866E-13 |
| GNPDA2 | 0.3618266 | 3.44406E-17 | 9.29619E-16 |
| GNPTAB | 0.4437142 | 5.75754E-26 | 8.72852E-24 |
| GNS | 0.3808562 | 5.13276E-19 | 1.89199E-17 |
| GOLIM4 | 0.3718657 | 3.87681E-18 | 1.2252E-16 |
| GOLPH3 | 0.3684655 | 8.194E-18 | 2.42519E-16 |
| GOPC | 0.3376944 | 4.83157E-15 | 8.70589E-14 |
| GORAB | 0.3813979 | 4.53494E-19 | 1.68705E-17 |
| GPAM | 0.4059976 | 1.27607E-21 | 7.96579E-20 |
| GPBP1L1 | 0.3274641 | 3.46235E-14 | 5.28875E-13 |
| GPM6B | 0.3219313 | 9.74319E-14 | 1.369E-12 |
| GPN3 | 0.3639032 | 2.20584E-17 | 6.07601E-16 |
| GPR137C | 0.3610851 | 4.03477E-17 | 1.06903E-15 |
| GPR141 | 0.3035365 | 2.61483E-12 | 2.8044E-11 |
| GPR180 | 0.4131269 | 2.11818E-22 | 1.56443E-20 |
| GPR34 | 0.3300613 | 2.11476E-14 | 3.3895E-13 |
| GPR75 | 0.3564797 | 1.06861E-16 | 2.61484E-15 |
| GPRIN3 | 0.3014801 | 3.72429E-12 | 3.90702E-11 |
| GPX8 | 0.3446211 | 1.22089E-15 | 2.44458E-14 |
| GRINL1A | 0.4172188 | 7.4098E-23 | 5.97615E-21 |
| GSTCD | 0.4581662 | 8.81413E-28 | 1.87073E-25 |
| GSTP1 | -0.358942 | 6.36061E-17 | 1.62135E-15 |
| GTF2A1 | 0.3302253 | 2.04961E-14 | 3.29293E-13 |
| GTF2H3 | 0.3711772 | 4.51435E-18 | 1.39606E-16 |
| GUCY1A3 | 0.3214034 | 1.07423E-13 | 1.49583E-12 |
| GUCY1B3 | 0.3808975 | 5.08462E-19 | 1.87768E-17 |
| GUK1 | -0.361938 | 3.3631E-17 | 9.10203E-16 |

| | | | |
|---|---|---|---|
| H2AFJ | -0.415712 | 1.09273E-22 | 8.50681E-21 |
| HACE1 | 0.4296953 | 2.75235E-24 | 2.83141E-22 |
| HCFC2 | 0.4818281 | 6.02458E-31 | 2.47905E-28 |
| HDGFRP3 | 0.3065843 | 1.54007E-12 | 1.73671E-11 |
| HDHD3 | -0.435672 | 5.41065E-25 | 6.61182E-23 |
| HEATR6 | 0.3077958 | 1.24569E-12 | 1.43116E-11 |
| HEATR7A | -0.304798 | 2.10191E-12 | 2.3121E-11 |
| HECTD2 | 0.4576758 | 1.01906E-27 | 2.14035E-25 |
| HEG1 | 0.3160121 | 2.87933E-13 | 3.70255E-12 |
| HELB | 0.3417781 | 2.1562E-15 | 4.1131E-14 |
| HELZ | 0.3827374 | 3.33532E-19 | 1.27127E-17 |
| HERC1 | 0.3040577 | 2.38955E-12 | 2.59314E-11 |
| HFE | 0.3540348 | 1.78067E-16 | 4.17485E-15 |
| HGS | -0.344867 | 1.16187E-15 | 2.34738E-14 |
| HIAT1 | 0.4152497 | 1.23055E-22 | 9.50638E-21 |
| HIP1R | -0.314659 | 3.67616E-13 | 4.61822E-12 |
| HIP1 | 0.3125839 | 5.33473E-13 | 6.5072E-12 |
| HIPK1 | 0.3470399 | 7.4909E-16 | 1.5555E-14 |
| HIPK3 | 0.3127985 | 5.13392E-13 | 6.27745E-12 |
| HIVEP1 | 0.3156142 | 3.09424E-13 | 3.95119E-12 |
| HIVEP3 | 0.3492839 | 4.74307E-16 | 1.02502E-14 |
| HLCS | 0.3252426 | 5.25885E-14 | 7.7567E-13 |
| HLTF | 0.4341436 | 8.22713E-25 | 9.64439E-23 |
| HMBS | -0.328502 | 2.84468E-14 | 4.41573E-13 |
| HMG20A | 0.3450282 | 1.12487E-15 | 2.27947E-14 |
| HMGA1 | -0.338319 | 4.27367E-15 | 7.81232E-14 |
| HMGN4 | 0.331719 | 1.54004E-14 | 2.52044E-13 |
| HMGXB4 | 0.3874399 | 1.12152E-19 | 4.68184E-18 |
| HMMR | 0.3468789 | 7.73944E-16 | 1.60052E-14 |
| HMOX2 | -0.336404 | 6.21945E-15 | 1.09427E-13 |
| HN1 | -0.31751 | 2.19371E-13 | 2.90044E-12 |
| HNRNPR | 0.3765084 | 1.37541E-18 | 4.71638E-17 |
| HNRNPUL1 | 0.3057931 | 1.76797E-12 | 1.97057E-11 |
| HNRPLL | 0.3355774 | 7.30651E-15 | 1.26347E-13 |
| HOOK2 | -0.346121 | 9.02337E-16 | 1.85462E-14 |
| HP1BP3 | 0.3754203 | 1.75613E-18 | 5.93112E-17 |
| HPCAL4 | 0.3156472 | 3.07585E-13 | 3.93019E-12 |
| HPS3 | 0.4003661 | 5.11435E-21 | 2.84079E-19 |
| HRAS | -0.364138 | 2.09708E-17 | 5.82416E-16 |
| HRH2 | 0.3522109 | 2.59886E-16 | 5.92099E-15 |
| HS2ST1 | 0.5459428 | 7.04931E-41 | 1.09335E-37 |
| HSD17B1 | -0.344456 | 1.2622E-15 | 2.51729E-14 |
| HSP90B3P | 0.4245456 | 1.08949E-23 | 9.8952E-22 |
| HSPA13 | 0.4884402 | 7.08567E-32 | 3.37512E-29 |
| HSPB1 | -0.436109 | 4.79818E-25 | 6.00905E-23 |
| HSPBP1 | -0.310817 | 7.30858E-13 | 8.74558E-12 |
| ID1 | -0.302545 | 3.10221E-12 | 3.29905E-11 |
| IER2 | -0.317823 | 2.0722E-13 | 2.74519E-12 |
| IFNAR2 | 0.3829795 | 3.15469E-19 | 1.21158E-17 |
| IFT172 | 0.3260514 | 4.51829E-14 | 6.73336E-13 |
| IFT52 | 0.329139 | 2.52075E-14 | 3.96457E-13 |
| IFT80 | 0.4177476 | 6.46252E-23 | 5.23308E-21 |

| | | | |
|---|---|---|---|
| IFT81 | 0.3440986 | 1.35603E-15 | 2.6911E-14 |
| IGFBP7 | 0.3084027 | 1.11969E-12 | 1.29599E-11 |
| IKBIP | 0.4396588 | 1.79534E-25 | 2.39731E-23 |
| IKZF4 | 0.3653772 | 1.60463E-17 | 4.50614E-16 |
| IKZF5 | 0.3509618 | 3.36214E-16 | 7.47419E-15 |
| IL17RE | -0.334744 | 8.59056E-15 | 1.46417E-13 |
| IL1F10 | -0.346212 | 8.85866E-16 | 1.82262E-14 |
| IL1F5 | -0.307063 | 1.41635E-12 | 1.61331E-11 |
| IL1R1 | 0.3020065 | 3.40281E-12 | 3.58844E-11 |
| IL1RAP | 0.329566 | 2.32409E-14 | 3.68692E-13 |
| IL6ST | 0.406055 | 1.25798E-21 | 7.87719E-20 |
| IL7R | 0.4422854 | 8.6101E-26 | 1.24878E-23 |
| ILVBL | -0.358101 | 7.59841E-17 | 1.91125E-15 |
| IMP3 | -0.348123 | 6.01091E-16 | 1.27711E-14 |
| IMP4 | -0.314823 | 3.56927E-13 | 4.49795E-12 |
| IMPA1 | 0.3837793 | 2.62374E-19 | 1.01932E-17 |
| IMPACT | 0.3072409 | 1.37296E-12 | 1.56844E-11 |
| IMPAD1 | 0.3388383 | 3.85885E-15 | 7.10556E-14 |
| IMPDH2 | -0.302051 | 3.37697E-12 | 3.56678E-11 |
| ING3 | 0.3063159 | 1.61395E-12 | 1.81192E-11 |
| INO80B | -0.372182 | 3.61461E-18 | 1.14593E-16 |
| INTS12 | 0.3106718 | 7.49871E-13 | 8.95714E-12 |
| INTS4L2 | 0.3189627 | 1.68274E-13 | 2.25293E-12 |
| IPO11 | 0.3042995 | 2.29161E-12 | 2.50573E-11 |
| IPP | 0.328502 | 2.84483E-14 | 4.41573E-13 |
| IQGAP2 | 0.3087407 | 1.055E-12 | 1.22676E-11 |
| IQGAP3 | 0.3108513 | 7.26358E-13 | 8.69689E-12 |
| IRAK3 | 0.3967373 | 1.23378E-20 | 6.39503E-19 |
| IRF2 | 0.3387623 | 3.91704E-15 | 7.19302E-14 |
| ITFG1 | 0.3250437 | 5.45847E-14 | 8.01012E-13 |
| ITGA1 | 0.3513984 | 3.07316E-16 | 6.90024E-15 |
| ITGA2 | 0.3137234 | 4.34983E-13 | 5.39727E-12 |
| ITGA4 | 0.3235332 | 7.23689E-14 | 1.04451E-12 |
| ITGA8 | 0.3223024 | 9.09598E-14 | 1.28613E-12 |
| ITGAV | 0.3874603 | 1.11619E-19 | 4.66926E-18 |
| ITGB1 | 0.3647067 | 1.85493E-17 | 5.17303E-16 |
| ITGB3 | 0.3892571 | 7.32552E-20 | 3.21097E-18 |
| ITPKC | -0.304585 | 2.1811E-12 | 2.39138E-11 |
| ITPR1 | 0.3016577 | 3.61266E-12 | 3.79782E-11 |
| ITSN1 | 0.3231824 | 7.7251E-14 | 1.10941E-12 |
| JAK1 | 0.430033 | 2.51287E-24 | 2.65272E-22 |
| JAZF1 | 0.31717 | 2.33375E-13 | 3.06549E-12 |
| JMJD1C | 0.3105643 | 7.64314E-13 | 9.11346E-12 |
| JMJD7-PLA2G4B | -0.421013 | 2.76211E-23 | 2.34989E-21 |
| JMJD8 | -0.322285 | 9.12511E-14 | 1.28908E-12 |
| JOSD2 | -0.333685 | 1.05464E-14 | 1.77502E-13 |
| JUP | -0.354511 | 1.61258E-16 | 3.80287E-15 |
| KANK2 | 0.4217419 | 2.28159E-23 | 1.94931E-21 |
| KATNAL1 | 0.376047 | 1.52572E-18 | 5.19646E-17 |
| KBTBD2 | 0.3063318 | 1.60949E-12 | 1.80792E-11 |
| KBTBD4 | 0.415786 | 1.07213E-22 | 8.37885E-21 |
| KBTBD7 | 0.3277063 | 3.30744E-14 | 5.06747E-13 |

| | | | |
|---|---|---|---|
| KCNK7 | -0.372206 | 3.59555E-18 | 1.14169E-16 |
| KCNT2 | 0.3499764 | 4.11614E-16 | 9.02105E-15 |
| KCTD12 | 0.3405347 | 2.76015E-15 | 5.14828E-14 |
| KCTD20 | 0.3884187 | 8.919E-20 | 3.77802E-18 |
| KDELC1 | 0.3563994 | 1.08676E-16 | 2.65603E-15 |
| KDELR3 | 0.3512252 | 3.18473E-16 | 7.13487E-15 |
| KDM3B | 0.3196061 | 1.49555E-13 | 2.0279E-12 |
| KDR | 0.3181364 | 1.95707E-13 | 2.60293E-12 |
| KIAA0090 | 0.442936 | 7.17004E-26 | 1.07089E-23 |
| KIAA0196 | 0.3284151 | 2.89209E-14 | 4.48218E-13 |
| KIAA0240 | 0.3537927 | 1.87258E-16 | 4.35992E-15 |
| KIAA0247 | 0.3189966 | 1.67232E-13 | 2.24046E-12 |
| KIAA0406 | 0.3508451 | 3.44382E-16 | 7.63053E-15 |
| KIAA0494 | 0.399126 | 6.9187E-21 | 3.72005E-19 |
| KIAA0586 | 0.4436228 | 5.90789E-26 | 8.88961E-24 |
| KIAA0664 | -0.372785 | 3.16184E-18 | 1.01841E-16 |
| KIAA0754 | 0.300903 | 4.11092E-12 | 4.2704E-11 |
| KIAA0776 | 0.3846956 | 2.12304E-19 | 8.44317E-18 |
| KIAA0947 | 0.3602907 | 4.77835E-17 | 1.24962E-15 |
| KIAA1009 | 0.3897462 | 6.52917E-20 | 2.89336E-18 |
| KIAA1033 | 0.314857 | 3.54747E-13 | 4.47328E-12 |
| KIAA1107 | 0.3557427 | 1.24701E-16 | 3.0112E-15 |
| KIAA1109 | 0.3527171 | 2.34055E-16 | 5.38727E-15 |
| KIAA1143 | 0.3842868 | 2.33359E-19 | 9.18989E-18 |
| KIAA1257 | -0.304745 | 2.12145E-12 | 2.33233E-11 |
| KIAA1279 | 0.338153 | 4.4157E-15 | 8.01745E-14 |
| KIAA1377 | 0.3418787 | 2.11345E-15 | 4.0392E-14 |
| KIAA1524 | 0.4161693 | 9.71422E-23 | 7.68109E-21 |
| KIAA1543 | -0.394001 | 2.37984E-20 | 1.15905E-18 |
| KIAA1586 | 0.3345357 | 8.94506E-15 | 1.51755E-13 |
| KIAA1712 | 0.336473 | 6.13605E-15 | 1.08243E-13 |
| KIDINS220 | 0.3341379 | 9.66167E-15 | 1.63293E-13 |
| KIF14 | 0.3071856 | 1.38633E-12 | 1.58282E-11 |
| KIF15 | 0.3059028 | 1.73453E-12 | 1.93651E-11 |
| KIF18A | 0.3336165 | 1.06869E-14 | 1.79268E-13 |
| KIF20A | 0.3876266 | 1.07362E-19 | 4.50051E-18 |
| KIF20B | 0.3823026 | 3.6857E-19 | 1.39166E-17 |
| KIF23 | 0.3097063 | 8.89717E-13 | 1.04603E-11 |
| KIF2A | 0.3709386 | 4.7586E-18 | 1.46485E-16 |
| KIF3A | 0.3192159 | 1.60648E-13 | 2.1652E-12 |
| KIF4B | 0.352239 | 2.58381E-16 | 5.89336E-15 |
| KIFC2 | -0.395383 | 1.70921E-20 | 8.59271E-19 |
| KITLG | 0.3143519 | 3.88531E-13 | 4.86279E-12 |
| KLC3 | -0.320671 | 1.22967E-13 | 1.69473E-12 |
| KLF16 | -0.391789 | 4.02862E-20 | 1.87596E-18 |
| KLHDC1 | 0.3499473 | 4.14072E-16 | 9.06508E-15 |
| KLHDC4 | -0.341316 | 2.36381E-15 | 4.49213E-14 |
| KLHL20 | 0.3885734 | 8.6013E-20 | 3.66656E-18 |
| KLHL28 | 0.3204663 | 1.27686E-13 | 1.75855E-12 |
| KLHL5 | 0.4107402 | 3.8828E-22 | 2.68113E-20 |
| KLHL8 | 0.3721706 | 3.62358E-18 | 1.14697E-16 |
| KLK10 | -0.350852 | 3.43864E-16 | 7.62742E-15 |

| | | | |
|---|---|---|---|
| KLK11 | -0.336118 | 6.57579E-15 | 1.14596E-13 |
| KLK12 | -0.371253 | 4.43906E-18 | 1.377E-16 |
| KLK8 | -0.306217 | 1.64201E-12 | 1.84195E-11 |
| KLRG2 | -0.341001 | 2.51641E-15 | 4.75078E-14 |
| KNTC1 | 0.3637729 | 2.26863E-17 | 6.24045E-16 |
| KRBA2 | 0.3599726 | 5.11247E-17 | 1.32497E-15 |
| KRIT1 | 0.3041995 | 2.33161E-12 | 2.53983E-11 |
| KRT17 | -0.362133 | 3.22526E-17 | 8.75676E-16 |
| KRT78 | -0.304228 | 2.32014E-12 | 2.53281E-11 |
| KRTDAP | -0.319192 | 1.61351E-13 | 2.17177E-12 |
| KTELC1 | 0.3256604 | 4.86258E-14 | 7.21443E-13 |
| LAD1 | -0.406853 | 1.03108E-21 | 6.4968E-20 |
| LAMC1 | 0.3665985 | 1.23119E-17 | 3.53626E-16 |
| LASS4 | -0.359566 | 5.57275E-17 | 1.43138E-15 |
| LATS1 | 0.3011397 | 3.94782E-12 | 4.12103E-11 |
| LCA5 | 0.465223 | 1.06389E-28 | 2.71535E-26 |
| LDLRAD3 | 0.3600788 | 4.99843E-17 | 1.29876E-15 |
| LDOC1L | 0.3007485 | 4.22086E-12 | 4.37784E-11 |
| LEMD3 | 0.3076767 | 1.272E-12 | 1.45806E-11 |
| LEPROT | 0.4537813 | 3.19954E-27 | 6.26334E-25 |
| LGALS7 | -0.301305 | 3.83758E-12 | 4.01542E-11 |
| LHFP | 0.3341583 | 9.62366E-15 | 1.62787E-13 |
| LIFR | 0.3961636 | 1.41667E-20 | 7.15898E-19 |
| LIMS1 | 0.3664771 | 1.26411E-17 | 3.62563E-16 |
| LIN52 | 0.4961639 | 5.4774E-33 | 3.28669E-30 |
| LIN54 | 0.3115865 | 6.37351E-13 | 7.67677E-12 |
| LIN9 | 0.3410394 | 2.49722E-15 | 4.71897E-14 |
| LIPA | 0.386252 | 1.47944E-19 | 6.01412E-18 |
| LIX1L | 0.3630119 | 2.6718E-17 | 7.29966E-16 |
| LLGL2 | -0.386163 | 1.51054E-19 | 6.12816E-18 |
| LMAN1 | 0.4819793 | 5.73984E-31 | 2.41109E-28 |
| LMBRD1 | 0.3195428 | 1.51302E-13 | 2.04883E-12 |
| LMBRD2 | 0.3301674 | 2.0724E-14 | 3.32689E-13 |
| LMLN | 0.3488367 | 5.19686E-16 | 1.1171E-14 |
| LMNA | -0.371508 | 4.19618E-18 | 1.30769E-16 |
| LMNB1 | 0.3094436 | 9.31996E-13 | 1.09191E-11 |
| LMTK3 | -0.34985 | 4.22372E-16 | 9.21676E-15 |
| LNPEP | 0.3493177 | 4.71039E-16 | 1.01905E-14 |
| LOC100132707 | 0.3623549 | 3.07596E-17 | 8.36985E-16 |
| LOC100170939 | 0.3143406 | 3.89317E-13 | 4.8696E-12 |
| LOC100216545 | -0.31064 | 7.54065E-13 | 9.00191E-12 |
| LOC100271831 | -0.474955 | 5.30768E-30 | 1.87752E-27 |
| LOC144486 | 0.3117762 | 6.1618E-13 | 7.444E-12 |
| LOC146880 | -0.316667 | 2.55684E-13 | 3.32819E-12 |
| LOC283404 | -0.303904 | 2.45371E-12 | 2.65753E-11 |
| LOC284441 | 0.3194388 | 1.54218E-13 | 2.08551E-12 |
| LOC402377 | 0.3179157 | 2.03744E-13 | 2.70091E-12 |
| LOC440957 | -0.320067 | 1.37422E-13 | 1.87727E-12 |
| LOC642852 | 0.3198242 | 1.43687E-13 | 1.95754E-12 |
| LOC646214 | 0.3129436 | 5.00229E-13 | 6.12765E-12 |
| LOC646999 | 0.3382926 | 4.29623E-15 | 7.84647E-14 |
| LOC650623 | 0.3132924 | 4.69946E-13 | 5.78481E-12 |

| | | | |
|---|---|---|---|
| LOC653653 | 0.4160461 | 1.00273E-22 | 7.89767E-21 |
| LOC728024 | 0.3066495 | 1.52261E-12 | 1.71991E-11 |
| LOC729020 | 0.3191732 | 1.6191E-13 | 2.17784E-12 |
| LOX | 0.3589725 | 6.31984E-17 | 1.613E-15 |
| LPAR4 | 0.3112414 | 6.77717E-13 | 8.13866E-12 |
| LPCAT2 | 0.3278048 | 3.2464E-14 | 4.98531E-13 |
| LPPR4 | 0.3297761 | 2.23291E-14 | 3.56187E-13 |
| LRCH2 | 0.3058987 | 1.73577E-12 | 1.93682E-11 |
| LRCH3 | 0.3432964 | 1.59254E-15 | 3.12357E-14 |
| LRIG2 | 0.3716154 | 4.09763E-18 | 1.28293E-16 |
| LRRC16B | -0.320167 | 1.34917E-13 | 1.84805E-12 |
| LRRC40 | 0.6311575 | 6.45218E-58 | 5.02989E-54 |
| LRRC8C | 0.3526926 | 2.35245E-16 | 5.40232E-15 |
| LRRCC1 | 0.3636457 | 2.33158E-17 | 6.39613E-16 |
| LSM7 | -0.346077 | 9.10365E-16 | 1.86731E-14 |
| LY6G6C | -0.347251 | 7.1764E-16 | 1.50257E-14 |
| LYNX1 | -0.367385 | 1.03753E-17 | 3.01437E-16 |
| LYRM7 | 0.3418307 | 2.13374E-15 | 4.07411E-14 |
| LYSMD3 | 0.4618511 | 2.94002E-28 | 6.81374E-26 |
| LYSMD4 | -0.387424 | 1.12557E-19 | 4.68902E-18 |
| LZTFL1 | 0.3945417 | 2.09102E-20 | 1.03034E-18 |
| MAGI3 | 0.3515512 | 2.9779E-16 | 6.71626E-15 |
| MAGT1 | 0.3493606 | 4.66926E-16 | 1.01124E-14 |
| MAML1 | 0.3001581 | 4.66829E-12 | 4.80483E-11 |
| MAN1A1 | 0.3494417 | 4.59245E-16 | 9.96745E-15 |
| MAN1A2 | 0.3737709 | 2.539E-18 | 8.29724E-17 |
| MAN2A1 | 0.4160026 | 1.01402E-22 | 7.95555E-21 |
| MAN2B2 | 0.3251853 | 5.31562E-14 | 7.83471E-13 |
| MANEA | 0.4963823 | 5.09E-33 | 3.20718E-30 |
| MAP2K2 | -0.419206 | 4.42597E-23 | 3.65741E-21 |
| MAP2K3 | -0.306436 | 1.58046E-12 | 1.77764E-11 |
| MAP3K2 | 0.3741289 | 2.34422E-18 | 7.71379E-17 |
| MAP3K3 | 0.3411356 | 2.44997E-15 | 4.63403E-14 |
| MAP3K7 | 0.3364487 | 6.16521E-15 | 1.08662E-13 |
| MAP4K3 | 0.3382141 | 4.36299E-15 | 7.93962E-14 |
| MAPK8 | 0.4748994 | 5.40116E-30 | 1.87765E-27 |
| MAPK9 | 0.3266088 | 4.06853E-14 | 6.12192E-13 |
| MAPRE2 | 0.3568189 | 9.95156E-17 | 2.45899E-15 |
| 45352 | 0.3180734 | 1.97968E-13 | 2.62953E-12 |
| 45359 | 0.3693557 | 6.74173E-18 | 2.02886E-16 |
| MAT2B | 0.4248887 | 9.94783E-24 | 9.20083E-22 |
| MATR3 | 0.4317712 | 1.57009E-24 | 1.72993E-22 |
| MBD5 | 0.3282041 | 3.01006E-14 | 4.65072E-13 |
| MBLAC2 | 0.3022203 | 3.28015E-12 | 3.4791E-11 |
| MBNL1 | 0.4360489 | 4.87787E-25 | 6.07114E-23 |
| MBTPS1 | 0.3588567 | 6.47664E-17 | 1.64884E-15 |
| MBTPS2 | 0.3552463 | 1.38336E-16 | 3.31268E-15 |
| MCAT | -0.344671 | 1.20867E-15 | 2.42974E-14 |
| MCFD2 | 0.3091024 | 9.89845E-13 | 1.157E-11 |
| MCM4 | 0.3289614 | 2.60725E-14 | 4.0847E-13 |
| MCM8 | 0.3900503 | 6.07777E-20 | 2.71721E-18 |
| MDC1 | 0.3007053 | 4.25217E-12 | 4.40805E-11 |

| | | | |
|---|---|---|---|
| MDH2 | -0.393246 | 2.84971E-20 | 1.36806E-18 |
| MDM1 | 0.4084165 | 6.97206E-22 | 4.47699E-20 |
| MDN1 | 0.3276426 | 3.34749E-14 | 5.12493E-13 |
| ME2 | 0.313296 | 4.69642E-13 | 5.7846E-12 |
| MECOM | 0.3152127 | 3.327E-13 | 4.21636E-12 |
| MED13L | 0.3223116 | 9.0806E-14 | 1.28486E-12 |
| MED14 | 0.3473648 | 7.01286E-16 | 1.47446E-14 |
| MED23 | 0.4099261 | 4.76915E-22 | 3.19469E-20 |
| METRNL | -0.40964 | 5.12579E-22 | 3.38857E-20 |
| METTL14 | 0.4199931 | 3.60539E-23 | 3.01641E-21 |
| MEX3C | 0.3055295 | 1.85102E-12 | 2.05744E-11 |
| MFAP3 | 0.4679704 | 4.60863E-29 | 1.30879E-26 |
| MFSD2B | -0.388929 | 7.91221E-20 | 3.41614E-18 |
| MFSD3 | -0.369218 | 6.94859E-18 | 2.08179E-16 |
| MFSD5 | -0.360518 | 4.55268E-17 | 1.20151E-15 |
| MFSD8 | 0.3089849 | 1.01056E-12 | 1.17917E-11 |
| MGAT2 | 0.447156 | 2.16679E-26 | 3.41321E-24 |
| MGA | 0.3324104 | 1.34847E-14 | 2.23275E-13 |
| MGC16025 | -0.338571 | 4.06774E-15 | 7.45616E-14 |
| MGC70857 | -0.301317 | 3.82966E-12 | 4.00922E-11 |
| MGC72080 | -0.31609 | 2.83898E-13 | 3.65532E-12 |
| MIA3 | 0.3799498 | 6.31149E-19 | 2.27645E-17 |
| MIB1 | 0.3433527 | 1.57469E-15 | 3.09157E-14 |
| MIER1 | 0.5825028 | 1.41836E-47 | 4.7664E-44 |
| MIER2 | -0.314106 | 4.06111E-13 | 5.06709E-12 |
| MIER3 | 0.3667117 | 1.20126E-17 | 3.45522E-16 |
| MIF | -0.352757 | 2.32121E-16 | 5.34886E-15 |
| MINPP1 | 0.3903642 | 5.64395E-20 | 2.53454E-18 |
| MIS12 | 0.3694547 | 6.59685E-18 | 1.9912E-16 |
| MITF | 0.3438416 | 1.42777E-15 | 2.82128E-14 |
| MKL2 | 0.3181163 | 1.96424E-13 | 2.61074E-12 |
| MLF2 | -0.321458 | 1.06339E-13 | 1.48176E-12 |
| MLL5 | 0.3189238 | 1.69474E-13 | 2.26749E-12 |
| MLST8 | -0.316184 | 2.79105E-13 | 3.5982E-12 |
| MMAB | -0.310191 | 8.16559E-13 | 9.68487E-12 |
| MMACHC | 0.3217431 | 1.00884E-13 | 1.41259E-12 |
| MMD | 0.3375206 | 4.99903E-15 | 8.98355E-14 |
| MMP16 | 0.307788 | 1.24738E-12 | 1.43229E-11 |
| MOB2 | -0.405955 | 1.28952E-21 | 8.02489E-20 |
| MOBKL1A | 0.3409529 | 2.54046E-15 | 4.7783E-14 |
| MOBKL1B | 0.3675143 | 1.00862E-17 | 2.93884E-16 |
| MOCS2 | 0.3083639 | 1.12735E-12 | 1.30337E-11 |
| MON2 | 0.305482 | 1.86638E-12 | 2.07338E-11 |
| MORC3 | 0.35242 | 2.48891E-16 | 5.68334E-15 |
| MORF4 | 0.3414088 | 2.32057E-15 | 4.41829E-14 |
| MOSPD2 | 0.3911564 | 4.68034E-20 | 2.14477E-18 |
| MPG | -0.310114 | 8.27747E-13 | 9.80027E-12 |
| MPHOSPH9 | 0.4594635 | 5.99744E-28 | 1.34363E-25 |
| MPST | -0.438969 | 2.17507E-25 | 2.82942E-23 |
| MRAS | 0.3006086 | 4.32295E-12 | 4.47223E-11 |
| MRPL12 | -0.425182 | 9.20286E-24 | 8.64735E-22 |
| MRPL14 | -0.310871 | 7.23844E-13 | 8.67193E-12 |

| | | | |
|---|---|---|---|
| MRPL23 | -0.364664 | 1.87203E-17 | 5.21351E-16 |
| MRPL24 | -0.303543 | 2.61192E-12 | 2.80277E-11 |
| MRPL27 | -0.320052 | 1.37796E-13 | 1.8811E-12 |
| MRPL28 | -0.31547 | 3.17593E-13 | 4.0427E-12 |
| MRPL38 | -0.326554 | 4.11101E-14 | 6.17662E-13 |
| MRPL41 | -0.360343 | 4.72515E-17 | 1.24207E-15 |
| MRPL4 | -0.374127 | 2.34516E-18 | 7.71379E-17 |
| MRPL53 | -0.348068 | 6.07803E-16 | 1.29001E-14 |
| MRPL55 | -0.310212 | 8.13617E-13 | 9.66134E-12 |
| MRPS12 | -0.345285 | 1.06814E-15 | 2.16668E-14 |
| MRPS18A | -0.326593 | 4.08098E-14 | 6.13608E-13 |
| MRPS25 | -0.344323 | 1.29622E-15 | 2.57748E-14 |
| MRPS2 | -0.334587 | 8.85589E-15 | 1.50431E-13 |
| MRPS34 | -0.379149 | 7.57261E-19 | 2.68342E-17 |
| MRPS5 | -0.343193 | 1.62568E-15 | 3.18238E-14 |
| MSH2 | 0.359943 | 5.14475E-17 | 1.33162E-15 |
| MSRB3 | 0.3098505 | 8.67325E-13 | 1.02209E-11 |
| MTBP | 0.3891076 | 7.58751E-20 | 3.31141E-18 |
| MTF2 | 0.4989974 | 2.1064E-33 | 1.41571E-30 |
| MTG1 | -0.304104 | 2.37035E-12 | 2.57647E-11 |
| MTHFD1 | 0.3039034 | 2.45416E-12 | 2.65753E-11 |
| MTMR6 | 0.3227858 | 8.3159E-14 | 1.18665E-12 |
| MTOR | 0.3551801 | 1.40261E-16 | 3.34685E-15 |
| MTRR | 0.4124259 | 2.53213E-22 | 1.82994E-20 |
| MTR | 0.3318357 | 1.50593E-14 | 2.47467E-13 |
| MTX1 | -0.319144 | 1.62779E-13 | 2.18807E-12 |
| MTX3 | 0.3685201 | 8.09671E-18 | 2.40079E-16 |
| MUDENG | 0.3600717 | 5.00592E-17 | 1.29903E-15 |
| MUTED | 0.3544664 | 1.6277E-16 | 3.83402E-15 |
| MUT | 0.3208044 | 1.19977E-13 | 1.65805E-12 |
| MVD | -0.439228 | 2.02394E-25 | 2.66724E-23 |
| MVK | -0.318434 | 1.85361E-13 | 2.47185E-12 |
| MXRA5 | 0.3046835 | 2.14403E-12 | 2.35458E-11 |
| MYBL1 | 0.4548653 | 2.33038E-27 | 4.69875E-25 |
| MYEOV2 | -0.321804 | 9.97563E-14 | 1.39777E-12 |
| MYO1H | -0.310566 | 7.64109E-13 | 9.11346E-12 |
| MYSM1 | 0.3324008 | 1.35097E-14 | 2.23275E-13 |
| MYST2 | 0.3082072 | 1.15885E-12 | 1.33596E-11 |
| N4BP2 | 0.3443331 | 1.29366E-15 | 2.57494E-14 |
| NAA10 | -0.327138 | 3.68224E-14 | 5.59496E-13 |
| NACA2 | -0.349502 | 4.53615E-16 | 9.85586E-15 |
| NACA | -0.31649 | 2.64066E-13 | 3.42623E-12 |
| NAGA | 0.3801612 | 6.01465E-19 | 2.18905E-17 |
| NAIP | 0.3059256 | 1.72764E-12 | 1.92988E-11 |
| NARFL | -0.328456 | 2.86966E-14 | 4.45084E-13 |
| NARG2 | 0.3057179 | 1.79129E-12 | 1.99546E-11 |
| NBN | 0.4202627 | 3.36036E-23 | 2.83494E-21 |
| NBPF10 | 0.3010687 | 3.99608E-12 | 4.16398E-11 |
| NBPF15 | 0.3168305 | 2.48227E-13 | 3.23738E-12 |
| NBPF16 | 0.3095495 | 9.14719E-13 | 1.07354E-11 |
| NBPF1 | 0.340761 | 2.63905E-15 | 4.94988E-14 |
| NBPF9 | 0.4108251 | 3.80035E-22 | 2.63321E-20 |

| | | | |
|---|---|---|---|
| NBR1 | 0.3199974 | 1.39187E-13 | 1.89752E-12 |
| NCEH1 | 0.3251676 | 5.33328E-14 | 7.85092E-13 |
| NCOA2 | 0.3142457 | 3.96025E-13 | 4.94431E-12 |
| NCSTN | 0.3873288 | 1.15101E-19 | 4.7851E-18 |
| NDNL2 | 0.3342998 | 9.36358E-15 | 1.58654E-13 |
| NDOR1 | -0.448798 | 1.3541E-26 | 2.23793E-24 |
| NDUFA11 | -0.306677 | 1.51537E-12 | 1.7127E-11 |
| NDUFA13 | -0.383364 | 2.88709E-19 | 1.11305E-17 |
| NDUFAB1 | -0.306627 | 1.5286E-12 | 1.72571E-11 |
| NDUFB10 | -0.355279 | 1.37405E-16 | 3.29429E-15 |
| NDUFB2 | -0.391427 | 4.38975E-20 | 2.02079E-18 |
| NDUFB7 | -0.362716 | 2.84671E-17 | 7.75651E-16 |
| NDUFS7 | -0.351092 | 3.27301E-16 | 7.31638E-15 |
| NECAB1 | 0.3333147 | 1.13282E-14 | 1.89238E-13 |
| NEDD1 | 0.4942835 | 1.02769E-32 | 5.75591E-30 |
| NEDD4 | 0.3289739 | 2.6011E-14 | 4.07822E-13 |
| NEK11 | 0.304896 | 2.06642E-12 | 2.27804E-11 |
| NEK1 | 0.3548653 | 1.49785E-16 | 3.556E-15 |
| NEK4 | 0.3924305 | 3.45984E-20 | 1.62612E-18 |
| NEK9 | 0.3299096 | 2.17683E-14 | 3.48345E-13 |
| NENF | -0.308124 | 1.17599E-12 | 1.35417E-11 |
| NEU3 | 0.3159113 | 2.93234E-13 | 3.76112E-12 |
| NF1 | 0.3296112 | 2.30417E-14 | 3.66395E-13 |
| NFATC2 | 0.3067394 | 1.49887E-12 | 1.6969E-11 |
| NFIC | 0.3089853 | 1.01049E-12 | 1.17917E-11 |
| NFKBIB | -0.303089 | 2.82474E-12 | 3.0167E-11 |
| NFKBIL1 | -0.316253 | 2.75628E-13 | 3.55793E-12 |
| NHLRC2 | 0.3064377 | 1.58E-12 | 1.77764E-11 |
| NHLRC3 | 0.3714605 | 4.24041E-18 | 1.3174E-16 |
| NHSL2 | 0.3029497 | 2.89329E-12 | 3.08174E-11 |
| NIPBL | 0.3041098 | 2.3681E-12 | 2.5754E-11 |
| NLRX1 | -0.409491 | 5.32223E-22 | 3.50693E-20 |
| NME2P1 | -0.354706 | 1.54847E-16 | 3.66452E-15 |
| NME2 | -0.378144 | 9.50969E-19 | 3.34631E-17 |
| NME3 | -0.309462 | 9.28908E-13 | 1.08893E-11 |
| NMRAL1 | -0.372834 | 3.12767E-18 | 1.00901E-16 |
| NNT | 0.3285039 | 2.84377E-14 | 4.41573E-13 |
| NOC4L | -0.308271 | 1.14591E-12 | 1.32407E-11 |
| NOL12 | -0.36047 | 4.59959E-17 | 1.21231E-15 |
| NOL3 | -0.359292 | 5.90685E-17 | 1.51334E-15 |
| NOXA1 | -0.325465 | 5.04454E-14 | 7.46244E-13 |
| NPAT | 0.338439 | 4.17434E-15 | 7.63768E-14 |
| NPEPL1 | -0.30378 | 2.50713E-12 | 2.70472E-11 |
| NPTN | 0.3801224 | 6.0681E-19 | 2.20452E-17 |
| NR2C2 | 0.3977152 | 9.74183E-21 | 5.12858E-19 |
| NR2F2 | 0.3124906 | 5.42439E-13 | 6.60857E-12 |
| NR2F6 | -0.414245 | 1.59207E-22 | 1.2068E-20 |
| NR3C1 | 0.3219421 | 9.72377E-14 | 1.36723E-12 |
| NRAS | 0.411594 | 3.1278E-22 | 2.22848E-20 |
| NRP1 | 0.3158352 | 2.97303E-13 | 3.80604E-12 |
| NSDHL | -0.395373 | 1.71317E-20 | 8.59271E-19 |
| NSL1 | 0.4310214 | 1.92384E-24 | 2.10817E-22 |

| | | | |
|---|---|---|---|
| NT5C | -0.425392 | 8.70425E-24 | 8.35732E-22 |
| NTHL1 | -0.351867 | 2.7903E-16 | 6.31435E-15 |
| NTN4 | 0.3908642 | 5.01528E-20 | 2.27755E-18 |
| NUAK1 | 0.3020135 | 3.39877E-12 | 3.58605E-11 |
| NUDT21 | 0.3088653 | 1.0321E-12 | 1.2029E-11 |
| NUDT22 | -0.384553 | 2.19451E-19 | 8.71021E-18 |
| NUDT4 | 0.3962806 | 1.37732E-20 | 6.99997E-19 |
| NUDT8 | -0.304221 | 2.32291E-12 | 2.53313E-11 |
| NUP107 | 0.3232826 | 7.5824E-14 | 1.09203E-12 |
| NUP155 | 0.3706168 | 5.10856E-18 | 1.56541E-16 |
| NUP160 | 0.401081 | 4.29426E-21 | 2.41858E-19 |
| NUP54 | 0.3586269 | 6.79918E-17 | 1.72036E-15 |
| ORC3L | 0.3766499 | 1.33229E-18 | 4.58414E-17 |
| OSBPL11 | 0.3212376 | 1.10762E-13 | 1.53914E-12 |
| OSBPL8 | 0.4665659 | 7.07493E-29 | 1.877E-26 |
| OSBPL9 | 0.3814978 | 4.43242E-19 | 1.65502E-17 |
| OSGIN1 | -0.331908 | 1.48525E-14 | 2.44466E-13 |
| OSMR | 0.3203984 | 1.29291E-13 | 1.77581E-12 |
| OTUB1 | -0.377506 | 1.09846E-18 | 3.80804E-17 |
| OTUD4 | 0.3438337 | 1.43002E-15 | 2.82128E-14 |
| OVOL1 | -0.319122 | 1.63434E-13 | 2.1925E-12 |
| OVOL2 | -0.336957 | 5.58246E-15 | 9.90837E-14 |
| OXR1 | 0.3279092 | 3.1829E-14 | 4.89526E-13 |
| P2RY12 | 0.3228442 | 8.22611E-14 | 1.1755E-12 |
| P2RY14 | 0.3005654 | 4.35494E-12 | 4.49839E-11 |
| PABPC4L | 0.3309771 | 1.77531E-14 | 2.87284E-13 |
| PABPC5 | 0.3074415 | 1.32554E-12 | 1.51599E-11 |
| PACSIN3 | -0.379582 | 6.8627E-19 | 2.45342E-17 |
| PALM2-AKAP2 | 0.3483772 | 5.70748E-16 | 1.21778E-14 |
| PAPD4 | 0.374119 | 2.34936E-18 | 7.71499E-17 |
| PAPL | -0.313647 | 4.41011E-13 | 5.45863E-12 |
| PAPOLA | 0.3091717 | 9.77813E-13 | 1.1436E-11 |
| PAPOLG | 0.3716411 | 4.07436E-18 | 1.27763E-16 |
| PAPSS2 | 0.3753957 | 1.76584E-18 | 5.95394E-17 |
| PAQR8 | 0.3443663 | 1.28506E-15 | 2.56034E-14 |
| PARP8 | 0.3109904 | 7.08631E-13 | 8.49978E-12 |
| PATL1 | 0.3093189 | 9.52747E-13 | 1.11493E-11 |
| PCDH18 | 0.3168678 | 2.46551E-13 | 3.21857E-12 |
| PCDHGA12 | 0.3136956 | 4.37163E-13 | 5.41766E-12 |
| PCDHGA9 | 0.3040106 | 2.40911E-12 | 2.61296E-11 |
| PCGF6 | 0.3524753 | 2.46059E-16 | 5.63143E-15 |
| PCNP | 0.3829054 | 3.2089E-19 | 1.23006E-17 |
| PCYT2 | -0.312604 | 5.31511E-13 | 6.49114E-12 |
| PDDC1 | -0.31365 | 4.40749E-13 | 5.45863E-12 |
| PDE3A | 0.3029642 | 2.88609E-12 | 3.0757E-11 |
| PDE4DIP | 0.335206 | 7.85382E-15 | 1.34937E-13 |
| PDE6D | 0.3294225 | 2.38843E-14 | 3.7771E-13 |
| PDF | -0.314321 | 3.90715E-13 | 4.88103E-12 |
| PDLIM2 | -0.307079 | 1.41233E-12 | 1.61068E-11 |
| PDPR | 0.3295712 | 2.32178E-14 | 3.68615E-13 |
| PDS5B | 0.3870747 | 1.22135E-19 | 5.04632E-18 |
| PES1 | -0.349391 | 4.64073E-16 | 1.00614E-14 |

| | | | |
|---|---|---|---|
| PGAP2 | -0.345519 | 1.01901E-15 | 2.07121E-14 |
| PGCP | 0.3501205 | 3.99634E-16 | 8.78716E-15 |
| PGLS | -0.422162 | 2.04323E-23 | 1.76814E-21 |
| PGM2L1 | 0.3470299 | 7.50598E-16 | 1.55703E-14 |
| PHC3 | 0.3446373 | 1.21694E-15 | 2.44016E-14 |
| PHF12 | 0.3039003 | 2.45551E-12 | 2.65756E-11 |
| PHF3 | 0.367915 | 9.24181E-18 | 2.70062E-16 |
| PHF6 | 0.4442587 | 4.93644E-26 | 7.54042E-24 |
| PHLDA2 | -0.310162 | 8.20741E-13 | 9.72875E-12 |
| PHLDB3 | -0.493881 | 1.1753E-32 | 6.40477E-30 |
| PHLPP2 | 0.3137545 | 4.32565E-13 | 5.37388E-12 |
| PHTF1 | 0.3691305 | 7.08321E-18 | 2.11583E-16 |
| PHTF2 | 0.3673697 | 1.04093E-17 | 3.01991E-16 |
| PI3 | -0.357401 | 8.80444E-17 | 2.19436E-15 |
| PIBF1 | 0.3150371 | 3.43414E-13 | 4.34122E-12 |
| PIGK | 1 | 1E-58 | 1E-54 |
| PIK3C2A | 0.3054747 | 1.86875E-12 | 2.07484E-11 |
| PIK3CA | 0.3008718 | 4.13291E-12 | 4.29104E-11 |
| PIK3CG | 0.3051593 | 1.97406E-12 | 2.18457E-11 |
| PIK3R1 | 0.3526463 | 2.3751E-16 | 5.44814E-15 |
| PIK3R4 | 0.3306584 | 1.8869E-14 | 3.04365E-13 |
| PIKFYVE | 0.3839447 | 2.52547E-19 | 9.8684E-18 |
| PJA2 | 0.3949555 | 1.89369E-20 | 9.38143E-19 |
| PKD2 | 0.4220432 | 2.10807E-23 | 1.80872E-21 |
| PKIA | 0.3153018 | 3.27391E-13 | 4.15953E-12 |
| PKN2 | 0.5049175 | 2.77829E-34 | 2.24074E-31 |
| PKP1 | -0.316651 | 2.56469E-13 | 3.33625E-12 |
| PKP3 | -0.368291 | 8.51233E-18 | 2.50927E-16 |
| PLCD1 | -0.317216 | 2.31433E-13 | 3.04198E-12 |
| PLCL1 | 0.3700354 | 5.80646E-18 | 1.76585E-16 |
| PLCL2 | 0.3155574 | 3.12619E-13 | 3.98441E-12 |
| PLDN | 0.3865855 | 1.3689E-19 | 5.5986E-18 |
| PLEKHA3 | 0.3067578 | 1.49407E-12 | 1.69431E-11 |
| PLEKHH3 | -0.329052 | 2.56263E-14 | 4.02416E-13 |
| PLEKHJ1 | -0.355578 | 1.29062E-16 | 3.10534E-15 |
| PLEKHM1 | -0.304632 | 2.16327E-12 | 2.37441E-11 |
| PLEKHM3 | 0.3324052 | 1.34983E-14 | 2.23275E-13 |
| PLK4 | 0.3596889 | 5.42978E-17 | 1.39644E-15 |
| PLOD2 | 0.4624986 | 2.42081E-28 | 5.67568E-26 |
| PLSCR4 | 0.3111137 | 6.93282E-13 | 8.32063E-12 |
| PLXNC1 | 0.3663229 | 1.30716E-17 | 3.73318E-16 |
| PMPCA | -0.31742 | 2.23005E-13 | 2.94463E-12 |
| PMVK | -0.413582 | 1.88612E-22 | 1.39816E-20 |
| PNRC2 | 0.3519894 | 2.72049E-16 | 6.17088E-15 |
| POC5 | 0.4043852 | 1.90404E-21 | 1.13921E-19 |
| POFUT2 | 0.3426559 | 1.81007E-15 | 3.49919E-14 |
| POLA1 | 0.3964713 | 1.31549E-20 | 6.73201E-19 |
| POLD4 | -0.32533 | 5.17315E-14 | 7.64708E-13 |
| POLDIP2 | -0.301789 | 3.53234E-12 | 3.71725E-11 |
| POLK | 0.5479734 | 3.14489E-41 | 5.2842E-38 |
| POLR2B | 0.3188725 | 1.71074E-13 | 2.28737E-12 |
| POLR2E | -0.329537 | 2.33706E-14 | 3.70457E-13 |

| | | | |
|---|---|---|---|
| POLR2L | -0.324587 | 5.94487E-14 | 8.70489E-13 |
| POR | -0.392612 | 3.3141E-20 | 1.56859E-18 |
| POT1 | 0.3866677 | 1.34294E-19 | 5.51479E-18 |
| PPAN | -0.388796 | 8.16433E-20 | 3.49368E-18 |
| PPAP2B | 0.332998 | 1.20418E-14 | 2.00826E-13 |
| PPDPF | -0.34203 | 2.05072E-15 | 3.93423E-14 |
| PPFIA3 | -0.340601 | 2.72395E-15 | 5.08547E-14 |
| PPIF | -0.391993 | 3.83899E-20 | 1.79595E-18 |
| PPIL4 | 0.4591314 | 6.61978E-28 | 1.45081E-25 |
| PPIP5K2 | 0.4763063 | 3.47389E-30 | 1.25079E-27 |
| PPM1K | 0.3120226 | 5.89697E-13 | 7.14118E-12 |
| PPP1R12A | 0.4612071 | 3.56537E-28 | 8.16915E-26 |
| PPP1R2P3 | 0.3225754 | 8.64696E-14 | 1.22867E-12 |
| PPP1R8 | 0.4128191 | 2.29101E-22 | 1.67368E-20 |
| PPP2R1A | -0.305836 | 1.75467E-12 | 1.95683E-11 |
| PPP3CB | 0.3029978 | 2.86941E-12 | 3.05954E-11 |
| PPP4C | -0.383374 | 2.88073E-19 | 1.11272E-17 |
| PPT1 | 0.343665 | 1.47921E-15 | 2.90978E-14 |
| PPTC7 | 0.3800772 | 6.13097E-19 | 2.21539E-17 |
| PPWD1 | 0.3571781 | 9.22794E-17 | 2.29707E-15 |
| PRDM2 | 0.3519834 | 2.72384E-16 | 6.17088E-15 |
| PRDM8 | 0.4088818 | 6.20323E-22 | 4.04776E-20 |
| PRDX3 | 0.3473057 | 7.09748E-16 | 1.49069E-14 |
| PRDX6 | -0.31557 | 3.11919E-13 | 3.97801E-12 |
| PRIM1 | 0.3197176 | 1.46527E-13 | 1.99354E-12 |
| PRIM2 | 0.4649318 | 1.16205E-28 | 2.89264E-26 |
| PRKAA1 | 0.4100861 | 4.58044E-22 | 3.08881E-20 |
| PRKACB | 0.4813518 | 7.01632E-31 | 2.8294E-28 |
| PRKAR1A | 0.3961779 | 1.4118E-20 | 7.15232E-19 |
| PRKCZ | -0.361184 | 3.95084E-17 | 1.04955E-15 |
| PRKD3 | 0.4690881 | 3.27196E-29 | 9.42466E-27 |
| PRKDC | 0.3155752 | 3.11616E-13 | 3.97665E-12 |
| PRKG1 | 0.3000655 | 4.74251E-12 | 4.87377E-11 |
| PRMT10 | 0.3299051 | 2.17868E-14 | 3.48365E-13 |
| PRMT6 | 0.3365746 | 6.01547E-15 | 1.06395E-13 |
| PRPS1 | 0.3680316 | 9.00943E-18 | 2.64421E-16 |
| PRPS2 | 0.313336 | 4.66283E-13 | 5.75025E-12 |
| PRR11 | 0.4251748 | 9.22076E-24 | 8.64735E-22 |
| PRR24 | -0.306215 | 1.64253E-12 | 1.84195E-11 |
| PRR5 | -0.414054 | 1.67163E-22 | 1.25298E-20 |
| PRR7 | -0.321527 | 1.04996E-13 | 1.46406E-12 |
| PRRC1 | 0.412759 | 2.32633E-22 | 1.69298E-20 |
| PRSS27 | -0.341251 | 2.39447E-15 | 4.54183E-14 |
| PSMC5 | -0.319711 | 1.46695E-13 | 1.99448E-12 |
| PSMD4 | -0.300673 | 4.27594E-12 | 4.42814E-11 |
| PTAR1 | 0.426073 | 7.26223E-24 | 7.10817E-22 |
| PTBP2 | 0.3580982 | 7.60216E-17 | 1.91125E-15 |
| PTCD2 | 0.3354147 | 7.54143E-15 | 1.29964E-13 |
| PTDSS2 | -0.313237 | 4.74652E-13 | 5.83561E-12 |
| PTER | 0.3158405 | 2.97015E-13 | 3.80478E-12 |
| PTGER2 | 0.308486 | 1.1034E-12 | 1.27787E-11 |
| PTGES2 | -0.448957 | 1.29344E-26 | 2.17331E-24 |

| | | | |
|---|---|---|---|
| PTOV1 | -0.303477 | 2.64192E-12 | 2.82895E-11 |
| PTPDC1 | 0.3218461 | 9.898E-14 | 1.38785E-12 |
| PTPLB | 0.3811909 | 4.75486E-19 | 1.7656E-17 |
| PTPN11 | 0.3227559 | 8.36219E-14 | 1.19073E-12 |
| PTPRB | 0.3165401 | 2.61661E-13 | 3.40159E-12 |
| PTPRM | 0.3165038 | 2.63391E-13 | 3.42188E-12 |
| PTRH1 | -0.348261 | 5.84437E-16 | 1.24435E-14 |
| PURA | 0.3374352 | 5.08343E-15 | 9.11086E-14 |
| PUS1 | -0.328503 | 2.84401E-14 | 4.41573E-13 |
| PUS7L | 0.3722963 | 3.52404E-18 | 1.12074E-16 |
| PUSL1 | -0.321621 | 1.03181E-13 | 1.44275E-12 |
| PVRL3 | 0.4654848 | 9.82708E-29 | 2.5403E-26 |
| PVRL4 | -0.300958 | 4.07259E-12 | 4.23932E-11 |
| PVT1 | -0.337228 | 5.29443E-15 | 9.43036E-14 |
| PWWP2A | 0.348945 | 5.08317E-16 | 1.09383E-14 |
| PWWP2B | -0.422204 | 2.021E-23 | 1.75644E-21 |
| PXK | 0.3741838 | 2.31565E-18 | 7.67324E-17 |
| PYCARD | -0.400014 | 5.57376E-21 | 3.0706E-19 |
| QKI | 0.454006 | 2.99636E-27 | 5.94059E-25 |
| QTRTD1 | 0.3150756 | 3.41038E-13 | 4.31389E-12 |
| RAB11FIP2 | 0.440384 | 1.46656E-25 | 2.02536E-23 |
| RAB23 | 0.5687614 | 5.8496E-45 | 1.47432E-41 |
| RAB24 | -0.335797 | 7.00018E-15 | 1.21362E-13 |
| RAB25 | -0.404426 | 1.88514E-21 | 1.13125E-19 |
| RAB28 | 0.3069409 | 1.44699E-12 | 1.6437E-11 |
| RAB2B | 0.3279423 | 3.16305E-14 | 4.86844E-13 |
| RAB33B | 0.3778411 | 1.01834E-18 | 3.55854E-17 |
| RAB3D | -0.32078 | 1.20514E-13 | 1.66433E-12 |
| RAB8B | 0.3997196 | 5.98788E-21 | 3.2808E-19 |
| RAB9B | 0.302256 | 3.26009E-12 | 3.45965E-11 |
| RABEP2 | -0.360312 | 4.75706E-17 | 1.24567E-15 |
| RABGGTA | -0.400394 | 5.08003E-21 | 2.82952E-19 |
| RACGAP1 | 0.3132142 | 4.76574E-13 | 5.85568E-12 |
| RAD17 | 0.3855643 | 1.73585E-19 | 7.014E-18 |
| RAD18 | 0.3200477 | 1.37905E-13 | 1.88131E-12 |
| RAD1 | 0.3299703 | 2.15179E-14 | 3.4461E-13 |
| RAD21 | 0.3922227 | 3.63491E-20 | 1.70444E-18 |
| RAD51AP1 | 0.3567063 | 1.01897E-16 | 2.50554E-15 |
| RANGAP1 | -0.379196 | 7.49149E-19 | 2.65935E-17 |
| RAP1A | 0.5312935 | 2.02983E-38 | 2.55797E-35 |
| RAPGEF2 | 0.3225392 | 8.70532E-14 | 1.23609E-12 |
| RAPGEF6 | 0.3658757 | 1.44037E-17 | 4.07323E-16 |
| RAPGEFL1 | -0.33497 | 8.22236E-15 | 1.40508E-13 |
| RASA1 | 0.3038021 | 2.49753E-12 | 2.69581E-11 |
| RASAL1 | -0.32867 | 2.75544E-14 | 4.30348E-13 |
| RASGRP3 | 0.3036714 | 2.5546E-12 | 2.75152E-11 |
| RASSF3 | 0.3068074 | 1.48117E-12 | 1.68158E-11 |
| RASSF7 | -0.367161 | 1.08947E-17 | 3.14714E-16 |
| RASSF8 | 0.4747474 | 5.66424E-30 | 1.90347E-27 |
| RBBP4 | 0.5301455 | 3.1271E-38 | 3.50287E-35 |
| RBBP5 | 0.3404371 | 2.81402E-15 | 5.23906E-14 |
| RBBP9 | 0.3164453 | 2.66197E-13 | 3.44944E-12 |

| | | | |
|---|---|---|---|
| RBL1 | 0.4297021 | 2.74735E-24 | 2.83141E-22 |
| RBL2 | 0.3280996 | 3.07022E-14 | 4.73641E-13 |
| RBM12B | 0.3776317 | 1.06769E-18 | 3.71168E-17 |
| RBM16 | 0.3231241 | 7.80923E-14 | 1.12069E-12 |
| RBM27 | 0.426006 | 7.393E-24 | 7.20121E-22 |
| RBM42 | -0.302863 | 2.93665E-12 | 3.12628E-11 |
| RBM43 | 0.4489186 | 1.30793E-26 | 2.17949E-24 |
| RBM9 | 0.3362212 | 6.44513E-15 | 1.13003E-13 |
| RCBTB1 | 0.3662341 | 1.33261E-17 | 3.80049E-16 |
| RCHY1 | 0.3449634 | 1.13966E-15 | 2.30712E-14 |
| RDH13 | -0.436392 | 4.43857E-25 | 5.59343E-23 |
| RECQL | 0.4713543 | 1.62747E-29 | 4.89772E-27 |
| REEP4 | -0.319202 | 1.6105E-13 | 2.16918E-12 |
| REPS2 | 0.3325305 | 1.31767E-14 | 2.18848E-13 |
| REST | 0.3373137 | 5.20585E-15 | 9.29722E-14 |
| REV3L | 0.3381507 | 4.41769E-15 | 8.01745E-14 |
| RFC1 | 0.3267935 | 3.92937E-14 | 5.93467E-13 |
| RFC3 | 0.3266654 | 4.02533E-14 | 6.06292E-13 |
| RFWD3 | 0.3232096 | 7.68606E-14 | 1.10459E-12 |
| RFXANK | -0.343213 | 1.61945E-15 | 3.17328E-14 |
| RGMB | 0.3235942 | 7.15515E-14 | 1.03345E-12 |
| RGPD3 | 0.3271746 | 3.6569E-14 | 5.56064E-13 |
| RGPD4 | 0.3498397 | 4.23303E-16 | 9.2271E-15 |
| RGS12 | -0.328 | 3.1289E-14 | 4.82325E-13 |
| RGS18 | 0.3164946 | 2.6383E-13 | 3.42537E-12 |
| RHOBTB1 | 0.3144574 | 3.81223E-13 | 4.77428E-12 |
| RHOBTB3 | 0.3845163 | 2.21299E-19 | 8.7663E-18 |
| RHOV | -0.349 | 5.02596E-16 | 1.08268E-14 |
| RIC8B | 0.4947201 | 8.88323E-33 | 5.1175E-30 |
| RICTOR | 0.3946268 | 2.04884E-20 | 1.01252E-18 |
| RIF1 | 0.3413705 | 2.33828E-15 | 4.4478E-14 |
| RLF | 0.3446584 | 1.21179E-15 | 2.4336E-14 |
| RMI1 | 0.366051 | 1.38664E-17 | 3.93785E-16 |
| RNF115 | 0.3689086 | 7.43634E-18 | 2.21803E-16 |
| RNF126 | -0.367944 | 9.18377E-18 | 2.69146E-16 |
| RNF138P1 | 0.3313836 | 1.64236E-14 | 2.67271E-13 |
| RNF160 | 0.397843 | 9.44518E-21 | 4.98542E-19 |
| RNF180 | 0.3222825 | 9.12961E-14 | 1.28908E-12 |
| RNF187 | -0.358374 | 7.17209E-17 | 1.8099E-15 |
| RNF208 | -0.439123 | 2.08416E-25 | 2.72876E-23 |
| RNF219 | 0.3529848 | 2.2143E-16 | 5.11713E-15 |
| RNF39 | -0.302108 | 3.34404E-12 | 3.53941E-11 |
| RNF41 | 0.3788507 | 8.10228E-19 | 2.86608E-17 |
| RNFT1 | 0.4370993 | 3.65134E-25 | 4.65962E-23 |
| RNGTT | 0.3711846 | 4.50701E-18 | 1.39593E-16 |
| RNH1 | -0.332612 | 1.2972E-14 | 2.15625E-13 |
| ROBLD3 | -0.338864 | 3.83933E-15 | 7.07608E-14 |
| ROCK1 | 0.3750236 | 1.91934E-18 | 6.42852E-17 |
| ROCK2 | 0.3539567 | 1.80982E-16 | 4.23335E-15 |
| ROMO1 | -0.328338 | 2.93466E-14 | 4.54467E-13 |
| ROR1 | 0.3890266 | 7.73321E-20 | 3.36045E-18 |
| RP2 | 0.3602131 | 4.85779E-17 | 1.26687E-15 |

| | | | |
|---|---|---|---|
| RPAP2 | 0.3510431 | 3.30636E-16 | 7.38273E-15 |
| RPGRIP1L | 0.4297653 | 2.70094E-24 | 2.82171E-22 |
| RPL10A | -0.320969 | 1.164E-13 | 1.61304E-12 |
| RPL10 | -0.335564 | 7.32568E-15 | 1.26462E-13 |
| RPL11 | -0.323907 | 6.74979E-14 | 9.79813E-13 |
| RPL12 | -0.334588 | 8.85466E-15 | 1.50431E-13 |
| RPL13AP20 | -0.327809 | 3.24365E-14 | 4.98488E-13 |
| RPL13AP3 | -0.372917 | 3.07075E-18 | 9.92237E-17 |
| RPL13A | -0.305647 | 1.81346E-12 | 2.01904E-11 |
| RPL13 | -0.416709 | 8.45186E-23 | 6.7625E-21 |
| RPL14 | -0.33497 | 8.22298E-15 | 1.40508E-13 |
| RPL18A | -0.391002 | 4.8541E-20 | 2.21433E-18 |
| RPL18 | -0.401967 | 3.45579E-21 | 1.99541E-19 |
| RPL19P12 | -0.328729 | 2.72484E-14 | 4.25898E-13 |
| RPL19 | -0.405839 | 1.32743E-21 | 8.21014E-20 |
| RPL22L1 | -0.340934 | 2.54982E-15 | 4.79142E-14 |
| RPL23A | -0.364194 | 2.07178E-17 | 5.76183E-16 |
| RPL23P8 | -0.316751 | 2.51818E-13 | 3.28209E-12 |
| RPL23 | -0.351489 | 3.01602E-16 | 6.77949E-15 |
| RPL24 | -0.308677 | 1.06696E-12 | 1.23995E-11 |
| RPL27A | -0.376401 | 1.40896E-18 | 4.82323E-17 |
| RPL28 | -0.355558 | 1.29623E-16 | 3.11513E-15 |
| RPL29 | -0.396543 | 1.2931E-20 | 6.64581E-19 |
| RPL31 | -0.302216 | 3.28266E-12 | 3.47994E-11 |
| RPL32 | -0.319404 | 1.55192E-13 | 2.09587E-12 |
| RPL34 | -0.328528 | 2.83056E-14 | 4.41397E-13 |
| RPL35 | -0.371713 | 4.01032E-18 | 1.2595E-16 |
| RPL36 | -0.39166 | 4.15447E-20 | 1.93011E-18 |
| RPL37A | -0.37944 | 7.08751E-19 | 2.52483E-17 |
| RPL38 | -0.398413 | 8.22622E-21 | 4.38797E-19 |
| RPL39 | -0.368536 | 8.06889E-18 | 2.39641E-16 |
| RPL3 | -0.304295 | 2.29353E-12 | 2.50647E-11 |
| RPL41 | -0.320277 | 1.32222E-13 | 1.81237E-12 |
| RPL4 | -0.375174 | 1.85576E-18 | 6.2259E-17 |
| RPL7A | -0.346325 | 8.65845E-16 | 1.78325E-14 |
| RPL8 | -0.358626 | 6.80022E-17 | 1.72036E-15 |
| RPLP0 | -0.336088 | 6.61422E-15 | 1.15067E-13 |
| RPLP1 | -0.38882 | 8.11699E-20 | 3.48219E-18 |
| RPLP2 | -0.412981 | 2.19857E-22 | 1.61199E-20 |
| RPS10 | -0.333403 | 1.11376E-14 | 1.86209E-13 |
| RPS12 | -0.337914 | 4.62757E-15 | 8.36072E-14 |
| RPS13 | -0.3043 | 2.29122E-12 | 2.50573E-11 |
| RPS15A | -0.343993 | 1.38505E-15 | 2.74599E-14 |
| RPS15 | -0.427192 | 5.38767E-24 | 5.35131E-22 |
| RPS16 | -0.348306 | 5.79124E-16 | 1.23434E-14 |
| RPS17 | -0.331737 | 1.53468E-14 | 2.51371E-13 |
| RPS19BP1 | -0.303663 | 2.55832E-12 | 2.75406E-11 |
| RPS19 | -0.364834 | 1.80478E-17 | 5.04014E-16 |
| RPS21 | -0.326969 | 3.80123E-14 | 5.76273E-13 |
| RPS24 | -0.361666 | 3.56431E-17 | 9.5568E-16 |
| RPS25 | -0.320683 | 1.22689E-13 | 1.69205E-12 |
| RPS27A | -0.337411 | 5.1081E-15 | 9.14694E-14 |

| | | | |
|---|---|---|---|
| RPS29 | -0.344751 | 1.18936E-15 | 2.39571E-14 |
| RPS2 | -0.353609 | 1.94528E-16 | 4.51874E-15 |
| RPS3 | -0.330792 | 1.83947E-14 | 2.96951E-13 |
| RPS4X | -0.319545 | 1.51235E-13 | 2.04883E-12 |
| RPS5 | -0.311436 | 6.54656E-13 | 7.87109E-12 |
| RPS6KA3 | 0.309807 | 8.74024E-13 | 1.02938E-11 |
| RPS9 | -0.38408 | 2.4476E-19 | 9.62006E-18 |
| RPUSD1 | -0.347076 | 7.43644E-16 | 1.54578E-14 |
| RQCD1 | 0.3965354 | 1.29534E-20 | 6.64581E-19 |
| RRAGC | 0.3264898 | 4.16069E-14 | 6.23547E-13 |
| RRM2B | 0.405117 | 1.58825E-21 | 9.61679E-20 |
| RRP1 | -0.410517 | 4.10846E-22 | 2.81765E-20 |
| RRP9 | -0.332431 | 1.34302E-14 | 2.22875E-13 |
| RSBN1 | 0.4237367 | 1.34943E-23 | 1.20392E-21 |
| RSPRY1 | 0.3900351 | 6.0995E-20 | 2.72089E-18 |
| RSRC1 | 0.3965382 | 1.29447E-20 | 6.64581E-19 |
| RYK | 0.3070294 | 1.42474E-12 | 1.62025E-11 |
| S100A11 | -0.331778 | 1.52267E-14 | 2.49606E-13 |
| S100A14 | -0.367918 | 9.23474E-18 | 2.70062E-16 |
| S100A16 | -0.40713 | 9.62084E-22 | 6.10016E-20 |
| S100A6 | -0.313932 | 4.19024E-13 | 5.21529E-12 |
| S100PBP | 0.4801572 | 1.02715E-30 | 4.06087E-28 |
| S100P | -0.374462 | 2.1761E-18 | 7.25234E-17 |
| S1PR3 | 0.3540349 | 1.78064E-16 | 4.17485E-15 |
| SACM1L | 0.302343 | 3.21171E-12 | 3.41369E-11 |
| SACS | 0.3426782 | 1.80203E-15 | 3.48699E-14 |
| SAPS2 | -0.336724 | 5.84237E-15 | 1.03424E-13 |
| SAR1B | 0.3116796 | 6.26879E-13 | 7.55966E-12 |
| SASS6 | 0.476784 | 2.98904E-30 | 1.11578E-27 |
| SAV1 | 0.3101094 | 8.28482E-13 | 9.80322E-12 |
| SBF2 | 0.3239131 | 6.74237E-14 | 9.79441E-13 |
| SBNO1 | 0.3276159 | 3.36447E-14 | 5.14703E-13 |
| SBSN | -0.340962 | 2.53589E-15 | 4.77824E-14 |
| SCAND1 | -0.41083 | 3.79564E-22 | 2.63321E-20 |
| SCARB2 | 0.366393 | 1.28741E-17 | 3.68721E-16 |
| SCLT1 | 0.4371734 | 3.57736E-25 | 4.59429E-23 |
| SCMH1 | 0.3042074 | 2.32842E-12 | 2.53772E-11 |
| SCNN1D | -0.355183 | 1.40182E-16 | 3.34685E-15 |
| SCP2 | 0.4627841 | 2.22174E-28 | 5.27024E-26 |
| SDCBP2 | -0.313913 | 4.2044E-13 | 5.22969E-12 |
| SEC22B | 0.4427686 | 7.51604E-26 | 1.11431E-23 |
| SEC23A | 0.3243335 | 6.23368E-14 | 9.10136E-13 |
| SEC23IP | 0.3588016 | 6.55265E-17 | 1.66399E-15 |
| SEC24D | 0.3701062 | 5.71672E-18 | 1.74118E-16 |
| SEC31A | 0.3186689 | 1.77565E-13 | 2.36946E-12 |
| SEC62 | 0.3512681 | 3.15675E-16 | 7.08004E-15 |
| SECISBP2L | 0.3207512 | 1.21159E-13 | 1.6721E-12 |
| SEL1L | 0.3863936 | 1.43146E-19 | 5.83082E-18 |
| SELO | -0.449646 | 1.06091E-26 | 1.81281E-24 |
| SEMA5A | 0.34064 | 2.70314E-15 | 5.05128E-14 |
| SENP1 | 0.4448182 | 4.21332E-26 | 6.4928E-24 |
| SENP6 | 0.3273278 | 3.55264E-14 | 5.41844E-13 |

| | | | |
|---|---|---|---|
| SENP7 | 0.3997027 | 6.01254E-21 | 3.28539E-19 |
| 45550 | 0.4570559 | 1.22385E-27 | 2.54397E-25 |
| 45537 | 0.3361482 | 6.53755E-15 | 1.14226E-13 |
| 45542 | 0.3605373 | 4.5341E-17 | 1.19818E-15 |
| SERF2 | -0.309894 | 8.60652E-13 | 1.01481E-11 |
| SERINC1 | 0.460898 | 3.91052E-28 | 8.8593E-26 |
| SERINC3 | 0.3233988 | 7.42031E-14 | 1.07021E-12 |
| SF3B5 | -0.317745 | 2.10201E-13 | 2.78285E-12 |
| SFN | -0.344314 | 1.29866E-15 | 2.5798E-14 |
| SFRS12 | 0.3050347 | 2.01727E-12 | 2.22873E-11 |
| SFRS13A | 0.4846333 | 2.44363E-31 | 1.07111E-28 |
| SGCB | 0.3928395 | 3.13917E-20 | 1.4893E-18 |
| SGCE | 0.3227722 | 8.33681E-14 | 1.18879E-12 |
| SGK269 | 0.3459467 | 9.3463E-16 | 1.91514E-14 |
| SGOL1 | 0.3019482 | 3.43705E-12 | 3.62265E-11 |
| SGOL2 | 0.3709933 | 4.7015E-18 | 1.44949E-16 |
| SGSM3 | -0.328283 | 2.96557E-14 | 4.58549E-13 |
| SGTB | 0.3779291 | 9.98295E-19 | 3.49455E-17 |
| SH2B3 | 0.3657204 | 1.4897E-17 | 4.20094E-16 |
| SH2D3A | -0.306341 | 1.60679E-12 | 1.8059E-11 |
| SH3D20 | -0.355671 | 1.26596E-16 | 3.05329E-15 |
| SH3GL1 | -0.367801 | 9.47488E-18 | 2.76472E-16 |
| SH3GLB1 | 0.4721736 | 1.26271E-29 | 3.91692E-27 |
| SH3GLB2 | -0.433747 | 9.16863E-25 | 1.06245E-22 |
| SHB | -0.325112 | 5.38942E-14 | 7.92033E-13 |
| SHE | 0.3439081 | 1.40887E-15 | 2.79047E-14 |
| SHFM1 | -0.305472 | 1.86975E-12 | 2.07484E-11 |
| SHPRH | 0.3453319 | 1.05811E-15 | 2.14852E-14 |
| SHQ1 | 0.3200735 | 1.37253E-13 | 1.87622E-12 |
| SIKE1 | 0.4003191 | 5.17337E-21 | 2.86568E-19 |
| SIRT1 | 0.3869258 | 1.26452E-19 | 5.20337E-18 |
| SIRT7 | -0.472868 | 1.01794E-29 | 3.25789E-27 |
| SLC10A7 | 0.4122793 | 2.6283E-22 | 1.89266E-20 |
| SLC15A4 | 0.3752163 | 1.83825E-18 | 6.1857E-17 |
| SLC1A3 | 0.3122001 | 5.71323E-13 | 6.92699E-12 |
| SLC25A10 | -0.419466 | 4.13631E-23 | 3.4463E-21 |
| SLC25A24 | 0.4870985 | 1.09809E-31 | 5.032E-29 |
| SLC25A39 | -0.357614 | 8.41913E-17 | 2.10093E-15 |
| SLC25A40 | 0.4011217 | 4.25173E-21 | 2.40134E-19 |
| SLC25A46 | 0.4178173 | 6.34695E-23 | 5.16022E-21 |
| SLC26A2 | 0.4485859 | 1.439E-26 | 2.35891E-24 |
| SLC27A4 | -0.383863 | 2.57339E-19 | 1.00169E-17 |
| SLC2A10 | 0.3296069 | 2.30607E-14 | 3.66409E-13 |
| SLC2A13 | 0.3579329 | 7.87192E-17 | 1.97415E-15 |
| SLC30A5 | 0.3447117 | 1.19886E-15 | 2.41245E-14 |
| SLC30A6 | 0.4200522 | 3.55016E-23 | 2.98258E-21 |
| SLC30A7 | 0.5679817 | 8.163E-45 | 1.82878E-41 |
| SLC33A1 | 0.325262 | 5.2398E-14 | 7.73427E-13 |
| SLC34A3 | -0.426974 | 5.71202E-24 | 5.64566E-22 |
| SLC35A3 | 0.302335 | 3.21615E-12 | 3.41661E-11 |
| SLC35A5 | 0.4244099 | 1.12935E-23 | 1.02112E-21 |
| SLC35B4 | 0.3419413 | 2.08726E-15 | 3.99672E-14 |

| | | | |
|---|---|---|---|
| SLC35D1 | 0.3663346 | 1.30386E-17 | 3.72903E-16 |
| SLC38A9 | 0.3429127 | 1.71956E-15 | 3.34345E-14 |
| SLC39A10 | 0.3639817 | 2.1689E-17 | 6.00707E-16 |
| SLC39A4 | -0.336097 | 6.60254E-15 | 1.14963E-13 |
| SLC41A2 | 0.3251664 | 5.33441E-14 | 7.85092E-13 |
| SLC5A10 | -0.338126 | 4.43925E-15 | 8.04934E-14 |
| SLC6A17 | 0.3708134 | 4.89185E-18 | 1.50128E-16 |
| SLC8A1 | 0.3546325 | 1.57235E-16 | 3.71668E-15 |
| SLC9A3R1 | -0.321593 | 1.03717E-13 | 1.44901E-12 |
| SLFN11 | 0.4220644 | 2.09638E-23 | 1.80638E-21 |
| SLPI | -0.32506 | 5.4417E-14 | 7.99133E-13 |
| SLURP1 | -0.329353 | 2.42009E-14 | 3.82416E-13 |
| SMAD1 | 0.3809426 | 5.03245E-19 | 1.86182E-17 |
| SMAD5 | 0.3100426 | 8.38331E-13 | 9.91394E-12 |
| SMARCA5 | 0.3548614 | 1.49908E-16 | 3.556E-15 |
| SMARCAD1 | 0.4024233 | 3.08946E-21 | 1.79002E-19 |
| SMARCD2 | -0.344834 | 1.16971E-15 | 2.35849E-14 |
| SMC1A | 0.3404701 | 2.7957E-15 | 5.20977E-14 |
| SMC2 | 0.3931688 | 2.90237E-20 | 1.38346E-18 |
| SMC3 | 0.3598229 | 5.27755E-17 | 1.36319E-15 |
| SMC4 | 0.384717 | 2.11258E-19 | 8.41817E-18 |
| SMOX | -0.324536 | 6.00261E-14 | 8.77669E-13 |
| SMPD2 | -0.447609 | 1.90344E-26 | 3.04595E-24 |
| SMURF2 | 0.306528 | 1.55529E-12 | 1.7529E-11 |
| SNAP23 | 0.4493104 | 1.16861E-26 | 1.98006E-24 |
| SNHG7 | -0.358391 | 7.14686E-17 | 1.80579E-15 |
| SNHG8 | -0.323614 | 7.12913E-14 | 1.03043E-12 |
| SNIP1 | 0.3798779 | 6.41566E-19 | 2.30586E-17 |
| SNRNP27 | 0.3212277 | 1.10965E-13 | 1.5409E-12 |
| SNRNP35 | -0.303565 | 2.60197E-12 | 2.79508E-11 |
| SNRNP48 | 0.3263412 | 4.27868E-14 | 6.39519E-13 |
| SNRNP70 | -0.346577 | 8.22779E-16 | 1.69802E-14 |
| SNX13 | 0.3367795 | 5.77944E-15 | 1.024E-13 |
| SNX4 | 0.3486945 | 5.34986E-16 | 1.14877E-14 |
| SOAT1 | 0.4319324 | 1.50288E-24 | 1.66498E-22 |
| SOCS4 | 0.4251524 | 9.27577E-24 | 8.65868E-22 |
| SOCS5 | 0.3777638 | 1.03629E-18 | 3.61499E-17 |
| SOX30 | 0.3768748 | 1.26648E-18 | 4.37261E-17 |
| SP3 | 0.3730402 | 2.98751E-18 | 9.66889E-17 |
| SP4 | 0.474797 | 5.57707E-30 | 1.90347E-27 |
| SPATA13 | 0.308892 | 1.02726E-12 | 1.19796E-11 |
| SPATA1 | 0.3034383 | 2.65953E-12 | 2.84478E-11 |
| SPATA2L | -0.488558 | 6.81725E-32 | 3.35259E-29 |
| SPATA5 | 0.3282875 | 2.96284E-14 | 4.58479E-13 |
| SPATA6 | 0.3001406 | 4.68222E-12 | 4.81672E-11 |
| SPCS3 | 0.4300686 | 2.4888E-24 | 2.64115E-22 |
| SPIN1 | 0.3800783 | 6.1294E-19 | 2.21539E-17 |
| SPINK7 | -0.30864 | 1.07387E-12 | 1.24654E-11 |
| SPOP | 0.3747513 | 2.03989E-18 | 6.80967E-17 |
| SPRED1 | 0.315286 | 3.2833E-13 | 4.16884E-12 |
| SPRED2 | 0.3096699 | 8.95472E-13 | 1.05218E-11 |
| SPRR1A | -0.331302 | 1.6681E-14 | 2.71023E-13 |

| | | | |
|---|---|---|---|
| SPRR1B | -0.319662 | 1.48026E-13 | 2.00851E-12 |
| SRBD1 | 0.4932342 | 1.45759E-32 | 7.73405E-30 |
| SRFBP1 | 0.4480736 | 1.66665E-26 | 2.68837E-24 |
| SSH2 | 0.3171207 | 2.35479E-13 | 3.0891E-12 |
| SSNA1 | -0.345794 | 9.63984E-16 | 1.96928E-14 |
| SSX2IP | 0.5081608 | 9.0043E-35 | 7.89364E-32 |
| ST14 | -0.313731 | 4.34363E-13 | 5.3929E-12 |
| ST3GAL2 | 0.3715 | 4.20353E-18 | 1.30796E-16 |
| STAG1 | 0.3529821 | 2.21557E-16 | 5.11713E-15 |
| STAM2 | 0.4632546 | 1.92839E-28 | 4.6846E-26 |
| STAP2 | -0.450233 | 8.95901E-27 | 1.62739E-24 |
| STARD10 | -0.353843 | 1.85309E-16 | 4.32451E-15 |
| STARD13 | 0.3430584 | 1.67019E-15 | 3.25688E-14 |
| STIL | 0.458733 | 7.45088E-28 | 1.6154E-25 |
| STK17B | 0.3979074 | 9.299E-21 | 4.92115E-19 |
| STK4 | 0.4140879 | 1.6573E-22 | 1.24687E-20 |
| STT3B | 0.3515099 | 3.00332E-16 | 6.75847E-15 |
| STUB1 | -0.394269 | 2.23187E-20 | 1.09226E-18 |
| STX2 | 0.3800969 | 6.10354E-19 | 2.21341E-17 |
| STX4 | -0.335204 | 7.85677E-15 | 1.34937E-13 |
| STXBP2 | -0.326496 | 4.15615E-14 | 6.23516E-13 |
| STXBP3 | 0.4497854 | 1.0193E-26 | 1.76279E-24 |
| STXBP4 | 0.3602079 | 4.86314E-17 | 1.26687E-15 |
| SUCNR1 | 0.3289498 | 2.61304E-14 | 4.09059E-13 |
| SULT2B1 | -0.401268 | 4.10251E-21 | 2.32356E-19 |
| SURF2 | -0.373077 | 2.96322E-18 | 9.60568E-17 |
| SUSD5 | 0.3950852 | 1.83572E-20 | 9.13914E-19 |
| SYNE1 | 0.3891952 | 7.43282E-20 | 3.25093E-18 |
| SYNJ1 | 0.4407517 | 1.3234E-25 | 1.84025E-23 |
| SYNJ2BP | 0.3046915 | 2.14108E-12 | 2.35263E-11 |
| SYTL1 | -0.340975 | 2.52946E-15 | 4.77095E-14 |
| TAF10 | -0.331238 | 1.68874E-14 | 2.74156E-13 |
| TAF1L | 0.315538 | 3.13716E-13 | 3.99587E-12 |
| TAF1 | 0.3021259 | 3.33378E-12 | 3.53041E-11 |
| TAF2 | 0.4028812 | 2.7602E-21 | 1.61785E-19 |
| TALDO1 | -0.4399 | 1.67878E-25 | 2.27176E-23 |
| TAOK3 | 0.3005148 | 4.39275E-12 | 4.53513E-11 |
| TAZ | -0.314504 | 3.78014E-13 | 4.73999E-12 |
| TBC1D15 | 0.3885494 | 8.64996E-20 | 3.67952E-18 |
| TBC1D1 | 0.3221359 | 9.38098E-14 | 1.32272E-12 |
| TBC1D8B | 0.323028 | 7.95E-14 | 1.14008E-12 |
| TBCCD1 | 0.3570561 | 9.46769E-17 | 2.34806E-15 |
| TBCEL | 0.3279785 | 3.14144E-14 | 4.83888E-13 |
| TBL3 | -0.381462 | 4.46878E-19 | 1.66551E-17 |
| TBRG4 | -0.303633 | 2.5715E-12 | 2.76529E-11 |
| TBX15 | 0.3241768 | 6.41873E-14 | 9.35122E-13 |
| TCEANC | 0.3324032 | 1.35034E-14 | 2.23275E-13 |
| TCEB2 | -0.313715 | 4.35662E-13 | 5.40237E-12 |
| TCF12 | 0.4104068 | 4.22422E-22 | 2.88722E-20 |
| TCF19 | 0.3485305 | 5.5319E-16 | 1.18157E-14 |
| TCF7 | 0.3340354 | 9.85527E-15 | 1.66147E-13 |
| TCTN1 | 0.3741351 | 2.34094E-18 | 7.71379E-17 |

| | | | |
|---|---|---|---|
| TCTN2 | 0.3444756 | 1.25715E-15 | 2.50969E-14 |
| TDRKH | 0.3050902 | 1.99789E-12 | 2.20974E-11 |
| TEAD1 | 0.3223527 | 9.01157E-14 | 1.27688E-12 |
| TECR | -0.316294 | 2.73601E-13 | 3.53629E-12 |
| TEK | 0.3014516 | 3.74257E-12 | 3.92211E-11 |
| TEX264 | -0.371722 | 4.00222E-18 | 1.25892E-16 |
| TEX9 | 0.3619722 | 3.33851E-17 | 9.04763E-16 |
| TFAM | 0.3411367 | 2.44945E-15 | 4.63403E-14 |
| TFPI | 0.3115531 | 6.41157E-13 | 7.71801E-12 |
| TGFBRAP1 | 0.3218694 | 9.85556E-14 | 1.38287E-12 |
| TGOLN2 | 0.3510321 | 3.31384E-16 | 7.39125E-15 |
| THAP10 | 0.3375043 | 5.01512E-15 | 9.00444E-14 |
| THAP1 | 0.356546 | 1.05384E-16 | 2.58185E-15 |
| THAP2 | 0.33242 | 1.34598E-14 | 2.23183E-13 |
| THAP4 | -0.356707 | 1.01883E-16 | 2.50554E-15 |
| THAP6 | 0.3419666 | 2.07679E-15 | 3.98044E-14 |
| THBS1 | 0.3061284 | 1.66761E-12 | 1.868E-11 |
| THOP1 | -0.303834 | 2.4836E-12 | 2.68505E-11 |
| THRAP3 | 0.3374432 | 5.0755E-15 | 9.10474E-14 |
| THUMPD1 | 0.350029 | 4.07203E-16 | 8.93409E-15 |
| TIAF1 | -0.381631 | 4.29926E-19 | 1.61126E-17 |
| TIAM2 | 0.3102071 | 8.14272E-13 | 9.66343E-12 |
| TICAM2 | 0.401709 | 3.68186E-21 | 2.10304E-19 |
| TIMELESS | 0.3041245 | 2.36207E-12 | 2.5711E-11 |
| TIMM13 | -0.408836 | 6.27529E-22 | 4.08157E-20 |
| TIMM16 | -0.32684 | 3.89521E-14 | 5.88748E-13 |
| TIMM8B | -0.372414 | 3.4329E-18 | 1.09695E-16 |
| TKT | -0.410236 | 4.41069E-22 | 3.00448E-20 |
| TLR6 | 0.3122732 | 5.63913E-13 | 6.84127E-12 |
| TM2D1 | 0.3900072 | 6.13981E-20 | 2.73282E-18 |
| TM6SF1 | 0.3548526 | 1.50181E-16 | 3.55829E-15 |
| TM7SF2 | -0.408565 | 6.7169E-22 | 4.35475E-20 |
| TM9SF2 | 0.3152019 | 3.33351E-13 | 4.2193E-12 |
| TMCO3 | 0.3318291 | 1.50785E-14 | 2.47579E-13 |
| TMED10P1 | 0.3743003 | 2.25622E-18 | 7.4946E-17 |
| TMED10 | 0.3380686 | 4.48947E-15 | 8.12648E-14 |
| TMED2 | 0.3429623 | 1.70259E-15 | 3.31364E-14 |
| TMED5 | 0.5015784 | 8.75202E-34 | 6.08507E-31 |
| TMED7 | 0.517945 | 2.79277E-36 | 2.81553E-33 |
| TMEFF1 | 0.3660827 | 1.37712E-17 | 3.92188E-16 |
| TMEM102 | -0.317088 | 2.36868E-13 | 3.10353E-12 |
| TMEM106B | 0.3449507 | 1.14257E-15 | 2.31069E-14 |
| TMEM115 | -0.301022 | 4.02787E-12 | 4.19494E-11 |
| TMEM120A | -0.303012 | 2.86249E-12 | 3.05378E-11 |
| TMEM133 | 0.3190927 | 1.64314E-13 | 2.20283E-12 |
| TMEM141 | -0.347153 | 7.32067E-16 | 1.52802E-14 |
| TMEM147 | -0.302259 | 3.2582E-12 | 3.45946E-11 |
| TMEM150C | 0.317373 | 2.24914E-13 | 2.96596E-12 |
| TMEM167A | 0.3034989 | 2.63185E-12 | 2.82116E-11 |
| TMEM167B | 0.4418426 | 9.74984E-26 | 1.39423E-23 |
| TMEM182 | 0.3018254 | 3.51023E-12 | 3.69591E-11 |
| TMEM184A | -0.372621 | 3.27901E-18 | 1.05111E-16 |

| | | | |
|---|---|---|---|
| TMEM184C | 0.428782 | 3.51903E-24 | 3.58355E-22 |
| TMEM191A | -0.388274 | 9.22655E-20 | 3.9001E-18 |
| TMEM194A | 0.4344263 | 7.61484E-25 | 8.97883E-23 |
| TMEM200A | 0.3577868 | 8.11811E-17 | 2.03084E-15 |
| TMEM209 | 0.3362412 | 6.41997E-15 | 1.1266E-13 |
| TMEM26 | 0.3499077 | 4.1745E-16 | 9.12912E-15 |
| TMEM2 | 0.3527947 | 2.30323E-16 | 5.31351E-15 |
| TMEM30A | 0.4327042 | 1.21845E-24 | 1.36487E-22 |
| TMEM38B | 0.3380682 | 4.48986E-15 | 8.12648E-14 |
| TMEM39A | 0.3726038 | 3.29164E-18 | 1.05348E-16 |
| TMEM40 | -0.314488 | 3.7912E-13 | 4.7509E-12 |
| TMEM43 | 0.3470875 | 7.41886E-16 | 1.54372E-14 |
| TMEM47 | 0.3102687 | 8.05433E-13 | 9.58109E-12 |
| TMEM48 | 0.3684396 | 8.24058E-18 | 2.43272E-16 |
| TMEM54 | -0.335068 | 8.06683E-15 | 1.38192E-13 |
| TMEM55A | 0.3226867 | 8.47031E-14 | 1.20527E-12 |
| TMEM56 | 0.3012824 | 3.85257E-12 | 4.02902E-11 |
| TMEM67 | 0.4140266 | 1.68347E-22 | 1.25718E-20 |
| TMEM79 | -0.310345 | 7.94602E-13 | 9.46341E-12 |
| TMF1 | 0.3191373 | 1.62977E-13 | 2.18928E-12 |
| TMOD2 | 0.3187608 | 1.74607E-13 | 2.33307E-12 |
| TMPO | 0.3597027 | 5.41394E-17 | 1.39414E-15 |
| TMPRSS13 | -0.311521 | 6.44839E-13 | 7.75769E-12 |
| TMUB1 | -0.36025 | 4.81977E-17 | 1.25882E-15 |
| TMX1 | 0.3763108 | 1.43789E-18 | 4.91394E-17 |
| TMX3 | 0.4193803 | 4.22963E-23 | 3.50955E-21 |
| TMX4 | 0.3426386 | 1.81634E-15 | 3.50794E-14 |
| TNFRSF9 | 0.3131197 | 4.84709E-13 | 5.952E-12 |
| TNFSF8 | 0.3038273 | 2.48666E-12 | 2.68551E-11 |
| TNIK | 0.335738 | 7.08157E-15 | 1.22668E-13 |
| TNPO1 | 0.3321441 | 1.41931E-14 | 2.34187E-13 |
| TNS3 | 0.4266671 | 6.19885E-24 | 6.09695E-22 |
| TOLLIP | -0.425287 | 8.94945E-24 | 8.51169E-22 |
| TOM1 | -0.344541 | 1.2407E-15 | 2.47932E-14 |
| TOMM40 | -0.300422 | 4.46268E-12 | 4.6026E-11 |
| TOMM7 | -0.317473 | 2.20843E-13 | 2.91799E-12 |
| TOP1MT | -0.335104 | 8.01053E-15 | 1.37344E-13 |
| TOP2A | 0.3981261 | 8.81909E-21 | 4.67946E-19 |
| TOPBP1 | 0.3361305 | 6.56015E-15 | 1.14422E-13 |
| TOR1AIP1 | 0.3904868 | 5.48293E-20 | 2.47875E-18 |
| TOR1B | 0.3540964 | 1.75803E-16 | 4.13137E-15 |
| TOX | 0.3108014 | 7.32823E-13 | 8.75869E-12 |
| TPI1 | -0.336521 | 6.07862E-15 | 1.07323E-13 |
| TPP1 | 0.309753 | 8.82407E-13 | 1.03864E-11 |
| TPRA1 | -0.389054 | 7.68442E-20 | 3.34646E-18 |
| TPRN | -0.432046 | 1.45718E-24 | 1.62327E-22 |
| TRABD | -0.435388 | 5.84986E-25 | 7.06292E-23 |
| TRADD | -0.314013 | 4.12972E-13 | 5.14315E-12 |
| TRAK2 | 0.4497731 | 1.0229E-26 | 1.76279E-24 |
| TRAM1 | 0.3671774 | 1.08546E-17 | 3.14006E-16 |
| TRAM2 | 0.3617185 | 3.52458E-17 | 9.46286E-16 |
| TRAP1 | -0.376572 | 1.3558E-18 | 4.65706E-17 |

| | | | |
|---|---|---|---|
| TRAPPC2L | -0.342467 | 1.87978E-15 | 3.62352E-14 |
| TRAPPC5 | -0.365332 | 1.62029E-17 | 4.53749E-16 |
| TREX2 | -0.301567 | 3.66911E-12 | 3.85314E-11 |
| TRIM11 | -0.441194 | 1.16938E-25 | 1.64882E-23 |
| TRIM23 | 0.433603 | 9.53693E-25 | 1.09882E-22 |
| TRIM29 | -0.336194 | 6.47927E-15 | 1.13404E-13 |
| TRIM32 | 0.3146912 | 3.65503E-13 | 4.59453E-12 |
| TRIM37 | 0.3839564 | 2.51865E-19 | 9.86087E-18 |
| TRIM44 | 0.3102181 | 8.12679E-13 | 9.65589E-12 |
| TRIM52 | 0.3326647 | 1.28405E-14 | 2.13792E-13 |
| TRIM59 | 0.3477292 | 6.51228E-16 | 1.37783E-14 |
| TRIO | 0.375424 | 1.75467E-18 | 5.93112E-17 |
| TRIP6 | -0.353831 | 1.85792E-16 | 4.33078E-15 |
| TRMT1 | -0.311766 | 6.17251E-13 | 7.45247E-12 |
| TRPM7 | 0.3232653 | 7.60685E-14 | 1.09477E-12 |
| TRUB1 | 0.3949561 | 1.89342E-20 | 9.38143E-19 |
| TSEN54 | -0.393965 | 2.40025E-20 | 1.16617E-18 |
| TSPAN2 | 0.4005136 | 4.93336E-21 | 2.75544E-19 |
| TSPO | -0.435779 | 5.2543E-25 | 6.49954E-23 |
| TSSC4 | -0.414437 | 1.51572E-22 | 1.15327E-20 |
| TSTD2 | 0.4356311 | 5.47189E-25 | 6.64637E-23 |
| TST | -0.373883 | 2.47616E-18 | 8.105E-17 |
| TTC17 | 0.395434 | 1.6883E-20 | 8.51032E-19 |
| TTC21B | 0.3109469 | 7.14132E-13 | 8.56067E-12 |
| TTC26 | 0.3467036 | 8.01931E-16 | 1.65669E-14 |
| TTC30B | 0.3316742 | 1.55335E-14 | 2.53838E-13 |
| TTC33 | 0.5182635 | 2.48942E-36 | 2.6418E-33 |
| TTC37 | 0.4500589 | 9.42044E-27 | 1.68092E-24 |
| TTC5 | 0.314717 | 3.6381E-13 | 4.57611E-12 |
| TTF2 | 0.3557732 | 1.23908E-16 | 2.99562E-15 |
| TTK | 0.3561027 | 1.15647E-16 | 2.81277E-15 |
| TTLL12 | -0.300247 | 4.59825E-12 | 4.73758E-11 |
| TTLL7 | 0.3832896 | 2.93733E-19 | 1.13026E-17 |
| TUBB2C | -0.375741 | 1.63416E-18 | 5.54707E-17 |
| TUBB4 | -0.350913 | 3.39584E-16 | 7.54079E-15 |
| TUFM | -0.331485 | 1.61064E-14 | 2.62533E-13 |
| TWSG1 | 0.3671023 | 1.10338E-17 | 3.18275E-16 |
| TXN2 | -0.317922 | 2.03516E-13 | 2.69967E-12 |
| TXNDC12 | 0.3216651 | 1.0235E-13 | 1.43211E-12 |
| TXNDC15 | 0.3505409 | 3.66596E-16 | 8.08717E-15 |
| TXNDC16 | 0.4242631 | 1.17411E-23 | 1.05216E-21 |
| TXNDC5 | 0.331505 | 1.60457E-14 | 2.61756E-13 |
| TXN | -0.335565 | 7.32423E-15 | 1.26462E-13 |
| TYSND1 | -0.4056 | 1.40875E-21 | 8.65995E-20 |
| TYW3 | 0.343893 | 1.41314E-15 | 2.79619E-14 |
| UBA52 | -0.398162 | 8.74276E-21 | 4.6512E-19 |
| UBE2J1 | 0.3085092 | 1.0989E-12 | 1.2734E-11 |
| UBE2W | 0.345767 | 9.69177E-16 | 1.97589E-14 |
| UBIAD1 | 0.3007586 | 4.21359E-12 | 4.37255E-11 |
| UBL5 | -0.305205 | 1.95857E-12 | 2.1722E-11 |
| UBQLN4 | -0.336431 | 6.18684E-15 | 1.08948E-13 |
| UBR1 | 0.3424097 | 1.90122E-15 | 3.65436E-14 |

| | | | |
|---|---|---|---|
| UBR2 | 0.307602 | 1.28877E-12 | 1.47561E-11 |
| UBR7 | 0.4058428 | 1.3262E-21 | 8.21014E-20 |
| UBTD2 | 0.3580584 | 7.66635E-17 | 1.92499E-15 |
| UBXN1 | -0.306435 | 1.58077E-12 | 1.77764E-11 |
| UBXN4 | 0.3055557 | 1.84258E-12 | 2.04919E-11 |
| UFSP1 | -0.365893 | 1.43507E-17 | 4.06396E-16 |
| UGGT2 | 0.4346728 | 7.11768E-25 | 8.49194E-23 |
| UHMK1 | 0.3128416 | 5.09451E-13 | 6.23304E-12 |
| UHRF1BP1L | 0.4112155 | 3.44274E-22 | 2.41028E-20 |
| UNC45A | -0.315398 | 3.21727E-13 | 4.09015E-12 |
| UPP1 | -0.384065 | 2.45623E-19 | 9.6352E-18 |
| UQCR10 | -0.307506 | 1.31066E-12 | 1.49982E-11 |
| UQCR11 | -0.344859 | 1.16379E-15 | 2.34889E-14 |
| USH1G | -0.300655 | 4.28873E-12 | 4.4391E-11 |
| USMG5 | -0.309895 | 8.60494E-13 | 1.01481E-11 |
| USP12 | 0.3428741 | 1.73286E-15 | 3.36282E-14 |
| USP16 | 0.3656417 | 1.51529E-17 | 4.26716E-16 |
| USP1 | 0.5029766 | 5.42129E-34 | 4.0485E-31 |
| USP24 | 0.3182978 | 1.90024E-13 | 2.53069E-12 |
| USP33 | 0.5993864 | 5.79632E-51 | 2.92178E-47 |
| USP37 | 0.3702448 | 5.54494E-18 | 1.69398E-16 |
| USP45 | 0.3128794 | 5.06012E-13 | 6.19474E-12 |
| USP49 | 0.328508 | 2.84158E-14 | 4.41573E-13 |
| USP51 | 0.3895778 | 6.79329E-20 | 2.98416E-18 |
| UTP20 | 0.3506657 | 3.57318E-16 | 7.89978E-15 |
| VAMP7 | 0.324875 | 5.63359E-14 | 8.26109E-13 |
| VAMP8 | -0.34862 | 5.43139E-16 | 1.16279E-14 |
| VANGL1 | 0.3088036 | 1.04339E-12 | 1.21536E-11 |
| VASH2 | 0.3049305 | 2.0541E-12 | 2.26569E-11 |
| VCPIP1 | 0.3140935 | 4.07008E-13 | 5.07514E-12 |
| VDAC2 | -0.327255 | 3.60212E-14 | 5.48147E-13 |
| VEZF1 | 0.3732762 | 2.83471E-18 | 9.21876E-17 |
| VGLL1 | -0.306687 | 1.51265E-12 | 1.71058E-11 |
| VPS13C | 0.324326 | 6.2424E-14 | 9.10748E-13 |
| VPS18 | -0.322646 | 8.53498E-14 | 1.21362E-12 |
| VPS28 | -0.340009 | 3.06264E-15 | 5.68096E-14 |
| VPS4A | -0.30706 | 1.41704E-12 | 1.61331E-11 |
| WASH3P | -0.313435 | 4.58116E-13 | 5.6619E-12 |
| WASH5P | -0.318308 | 1.89674E-13 | 2.5277E-12 |
| WBP11P1 | 0.3373561 | 5.16287E-15 | 9.22863E-14 |
| WDFY1 | 0.3358427 | 6.93859E-15 | 1.20502E-13 |
| WDHD1 | 0.3974095 | 1.04895E-20 | 5.50778E-19 |
| WDR11 | 0.3578892 | 7.94477E-17 | 1.98994E-15 |
| WDR13 | -0.303282 | 2.73199E-12 | 2.91919E-11 |
| WDR18 | -0.30165 | 3.61737E-12 | 3.80078E-11 |
| WDR19 | 0.3382537 | 4.3292E-15 | 7.89952E-14 |
| WDR31 | 0.3197003 | 1.46992E-13 | 1.99582E-12 |
| WDR35 | 0.4258018 | 7.80568E-24 | 7.53043E-22 |
| WDR36 | 0.3134326 | 4.58276E-13 | 5.6619E-12 |
| WDR48 | 0.3228375 | 8.23639E-14 | 1.17614E-12 |
| WDR74 | -0.325986 | 4.57456E-14 | 6.81217E-13 |
| WDR7 | 0.3550301 | 1.44723E-16 | 3.44515E-15 |

| | | | |
|---|---|---|---|
| WIBG | -0.335435 | 7.5116E-15 | 1.29561E-13 |
| WRB | 0.3305905 | 1.91155E-14 | 3.07849E-13 |
| XAB2 | -0.303622 | 2.57657E-12 | 2.76927E-11 |
| XRN1 | 0.3302437 | 2.04243E-14 | 3.28402E-13 |
| XRN2 | 0.3674723 | 1.01792E-17 | 2.96165E-16 |
| YIPF5 | 0.4508043 | 7.59648E-27 | 1.39243E-24 |
| YTHDF3 | 0.3382039 | 4.37175E-15 | 7.94839E-14 |
| YY2 | 0.3259277 | 4.62455E-14 | 6.88154E-13 |
| ZBTB10 | 0.3463336 | 8.64308E-16 | 1.78191E-14 |
| ZBTB11 | 0.3219901 | 9.63768E-14 | 1.35607E-12 |
| ZBTB1 | 0.4025818 | 2.97129E-21 | 1.7315E-19 |
| ZBTB2 | 0.3556547 | 1.27017E-16 | 3.0598E-15 |
| ZBTB38 | 0.3166876 | 2.54751E-13 | 3.31818E-12 |
| ZBTB41 | 0.3781034 | 9.59693E-19 | 3.37113E-17 |
| ZBTB6 | 0.335178 | 7.89663E-15 | 1.35506E-13 |
| ZBTB8A | 0.4183796 | 5.48598E-23 | 4.47829E-21 |
| ZC3H7A | 0.4511956 | 6.78362E-27 | 1.26646E-24 |
| ZCCHC11 | 0.4559086 | 1.71581E-27 | 3.49453E-25 |
| ZCCHC4 | 0.3777187 | 1.04692E-18 | 3.64576E-17 |
| ZCCHC8 | 0.3000739 | 4.73576E-12 | 4.8693E-11 |
| ZCCHC9 | 0.3125486 | 5.36843E-13 | 6.54436E-12 |
| ZDHHC17 | 0.5134192 | 1.41203E-35 | 1.29412E-32 |
| ZDHHC20 | 0.329342 | 2.42529E-14 | 3.82937E-13 |
| ZEB1 | 0.3201117 | 1.36293E-13 | 1.86437E-12 |
| ZEB2 | 0.3437417 | 1.45666E-15 | 2.86823E-14 |
| ZFP106 | 0.3050465 | 2.01312E-12 | 2.22536E-11 |
| ZFP161 | 0.3726231 | 3.27754E-18 | 1.05111E-16 |
| ZFP1 | 0.4519545 | 5.44458E-27 | 1.02597E-24 |
| ZFP37 | 0.3277221 | 3.29755E-14 | 5.05794E-13 |
| ZFP90 | 0.3472741 | 7.14313E-16 | 1.49765E-14 |
| ZFP91 | 0.3105152 | 7.71E-13 | 9.18775E-12 |
| ZFPM2 | 0.3701311 | 5.68548E-18 | 1.73429E-16 |
| ZFR | 0.4234459 | 1.45712E-23 | 1.2886E-21 |
| ZFX | 0.3593715 | 5.80787E-17 | 1.48987E-15 |
| ZFYVE16 | 0.3822467 | 3.7333E-19 | 1.407E-17 |
| ZGPAT | -0.351114 | 3.25863E-16 | 7.29231E-15 |
| ZKSCAN4 | 0.340696 | 2.67331E-15 | 5.00482E-14 |
| ZMPSTE24 | 0.4253709 | 8.75305E-24 | 8.36434E-22 |
| ZMYM1 | 0.4019578 | 3.46374E-21 | 1.99541E-19 |
| ZMYM4 | 0.4155086 | 1.1514E-22 | 8.9291E-21 |
| ZMYM6 | 0.3900943 | 6.015E-20 | 2.69512E-18 |
| ZMYND19 | -0.34243 | 1.89373E-15 | 3.64343E-14 |
| ZNF124 | 0.3154638 | 3.17953E-13 | 4.04473E-12 |
| ZNF132 | 0.3152079 | 3.32989E-13 | 4.21738E-12 |
| ZNF136 | 0.4246451 | 1.06115E-23 | 9.76982E-22 |
| ZNF146 | 0.3530742 | 2.17368E-16 | 5.03191E-15 |
| ZNF148 | 0.3073745 | 1.34121E-12 | 1.53304E-11 |
| ZNF175 | 0.3795013 | 6.98942E-19 | 2.49429E-17 |
| ZNF17 | 0.3952596 | 1.76048E-20 | 8.8081E-19 |
| ZNF180 | 0.40979 | 4.93565E-22 | 3.28947E-20 |
| ZNF181 | 0.3292843 | 2.45208E-14 | 3.86865E-13 |
| ZNF184 | 0.3787481 | 8.29314E-19 | 2.92845E-17 |

| | | | |
|---|---|---|---|
| ZNF187 | 0.3881212 | 9.56307E-20 | 4.02547E-18 |
| ZNF192 | 0.4190285 | 4.6351E-23 | 3.79909E-21 |
| ZNF205 | -0.349856 | 4.21921E-16 | 9.21676E-15 |
| ZNF214 | 0.3365654 | 6.0263E-15 | 1.06493E-13 |
| ZNF221 | 0.3335788 | 1.0765E-14 | 1.80427E-13 |
| ZNF222 | 0.3172381 | 2.30505E-13 | 3.03175E-12 |
| ZNF225 | 0.4675833 | 5.18761E-29 | 1.45275E-26 |
| ZNF227 | 0.3685352 | 8.07004E-18 | 2.39641E-16 |
| ZNF22 | 0.3973789 | 1.05672E-20 | 5.53419E-19 |
| ZNF230 | 0.3748486 | 1.99597E-18 | 6.6741E-17 |
| ZNF23 | 0.3134235 | 4.5903E-13 | 5.66774E-12 |
| ZNF25 | 0.3371412 | 5.38475E-15 | 9.56588E-14 |
| ZNF260 | 0.3670646 | 1.11247E-17 | 3.2044E-16 |
| ZNF268 | 0.3629113 | 2.73009E-17 | 7.44882E-16 |
| ZNF280D | 0.3566896 | 1.02256E-16 | 2.51079E-15 |
| ZNF281 | 0.4534014 | 3.57449E-27 | 6.93005E-25 |
| ZNF284 | 0.3843624 | 2.29319E-19 | 9.04844E-18 |
| ZNF28 | 0.3258357 | 4.70515E-14 | 6.99631E-13 |
| ZNF292 | 0.3336225 | 1.06745E-14 | 1.79209E-13 |
| ZNF295 | 0.3426897 | 1.79789E-15 | 3.48231E-14 |
| ZNF296 | -0.396278 | 1.37826E-20 | 6.99997E-19 |
| ZNF302 | 0.3456329 | 9.9577E-16 | 2.02601E-14 |
| ZNF347 | 0.3338069 | 1.03007E-14 | 1.73511E-13 |
| ZNF354C | 0.3597573 | 5.35153E-17 | 1.37983E-15 |
| ZNF367 | 0.3009304 | 4.09166E-12 | 4.25259E-11 |
| ZNF37A | 0.3059572 | 1.71816E-12 | 1.92036E-11 |
| ZNF382 | 0.3576238 | 8.40171E-17 | 2.09918E-15 |
| ZNF385A | -0.343182 | 1.62943E-15 | 3.18664E-14 |
| ZNF397OS | 0.3944262 | 2.14966E-20 | 1.05459E-18 |
| ZNF408 | -0.350533 | 3.67156E-16 | 8.09067E-15 |
| ZNF417 | 0.347197 | 7.25587E-16 | 1.51764E-14 |
| ZNF41 | 0.4130152 | 2.17939E-22 | 1.60376E-20 |
| ZNF420 | 0.3480345 | 6.12007E-16 | 1.29757E-14 |
| ZNF432 | 0.3082181 | 1.15663E-12 | 1.33416E-11 |
| ZNF441 | 0.3416716 | 2.20239E-15 | 4.19724E-14 |
| ZNF451 | 0.3588384 | 6.50184E-17 | 1.65317E-15 |
| ZNF45 | 0.4727606 | 1.05236E-29 | 3.31543E-27 |
| ZNF461 | 0.4054452 | 1.46394E-21 | 8.91762E-20 |
| ZNF468 | 0.3255605 | 4.95459E-14 | 7.34014E-13 |
| ZNF484 | 0.4229648 | 1.65418E-23 | 1.45014E-21 |
| ZNF487 | 0.339721 | 3.24223E-15 | 5.99753E-14 |
| ZNF507 | 0.3035434 | 2.61171E-12 | 2.80277E-11 |
| ZNF510 | 0.4120748 | 2.76849E-22 | 1.98651E-20 |
| ZNF524 | -0.352093 | 2.66303E-16 | 6.05352E-15 |
| ZNF527 | 0.3361605 | 6.52193E-15 | 1.14052E-13 |
| ZNF529 | 0.3603277 | 4.74083E-17 | 1.24303E-15 |
| ZNF532 | 0.3264112 | 4.22268E-14 | 6.31617E-13 |
| ZNF546 | 0.3285668 | 2.81004E-14 | 4.38535E-13 |
| ZNF548 | 0.3505649 | 3.64792E-16 | 8.05619E-15 |
| ZNF551 | 0.3909883 | 4.87027E-20 | 2.21669E-18 |
| ZNF559 | 0.342633 | 1.81835E-15 | 3.50846E-14 |
| ZNF566 | 0.3621312 | 3.22684E-17 | 8.75676E-16 |

| | | | |
|---|---|---|---|
| ZNF567 | 0.411475 | 3.22365E-22 | 2.27772E-20 |
| ZNF581 | -0.346947 | 7.63264E-16 | 1.58167E-14 |
| ZNF585B | 0.3718295 | 3.90799E-18 | 1.2312E-16 |
| ZNF593 | -0.366071 | 1.38074E-17 | 3.92664E-16 |
| ZNF598 | -0.361774 | 3.4828E-17 | 9.36317E-16 |
| ZNF599 | 0.3560468 | 1.1701E-16 | 2.83566E-15 |
| ZNF611 | 0.3157459 | 3.02142E-13 | 3.86309E-12 |
| ZNF615 | 0.3218928 | 9.81287E-14 | 1.37783E-12 |
| ZNF616 | 0.3036954 | 2.54403E-12 | 2.7416E-11 |
| ZNF619 | 0.3471237 | 7.36461E-16 | 1.53401E-14 |
| ZNF621 | 0.3172931 | 2.2821E-13 | 3.00352E-12 |
| ZNF627 | 0.4276935 | 4.71186E-24 | 4.70323E-22 |
| ZNF641 | 0.4036086 | 2.30701E-21 | 1.36411E-19 |
| ZNF643 | 0.3403549 | 2.86022E-15 | 5.32016E-14 |
| ZNF644 | 0.5611907 | 1.43313E-43 | 2.88962E-40 |
| ZNF668 | -0.34081 | 2.61363E-15 | 4.90676E-14 |
| ZNF678 | 0.388909 | 7.94986E-20 | 3.41776E-18 |
| ZNF680 | 0.3561796 | 1.13799E-16 | 2.77452E-15 |
| ZNF684 | 0.5884235 | 9.6854E-49 | 3.90574E-45 |
| ZNF689 | 0.4500812 | 9.36007E-27 | 1.68092E-24 |
| ZNF699 | 0.3855955 | 1.72333E-19 | 6.9774E-18 |
| ZNF701 | 0.3471783 | 7.2834E-16 | 1.52182E-14 |
| ZNF709 | 0.3163171 | 2.72458E-13 | 3.52589E-12 |
| ZNF718 | 0.3539623 | 1.80774E-16 | 4.23335E-15 |
| ZNF765 | 0.3931715 | 2.90053E-20 | 1.38346E-18 |
| ZNF776 | 0.3269576 | 3.80968E-14 | 5.7712E-13 |
| ZNF780B | 0.343096 | 1.65767E-15 | 3.23559E-14 |
| ZNF791 | 0.3854548 | 1.78052E-19 | 7.18013E-18 |
| ZNF804A | 0.313568 | 4.47287E-13 | 5.53291E-12 |
| ZNF813 | 0.3077497 | 1.25581E-12 | 1.44032E-11 |
| ZNF81 | 0.3290964 | 2.54122E-14 | 3.99366E-13 |
| ZNF826 | 0.3067434 | 1.49784E-12 | 1.6969E-11 |
| ZNF827 | 0.4138971 | 1.74014E-22 | 1.29471E-20 |
| ZNF828 | 0.3147901 | 3.59052E-13 | 4.51908E-12 |
| ZNF845 | 0.3036964 | 2.54358E-12 | 2.7416E-11 |
| ZNF92 | 0.3353689 | 7.60895E-15 | 1.31016E-13 |
| ZNHIT6 | 0.3942128 | 2.26222E-20 | 1.10443E-18 |
| ZRANB2 | 0.3446351 | 1.21748E-15 | 2.44016E-14 |
| ZRANB3 | 0.3906176 | 5.31617E-20 | 2.40876E-18 |
| ZSCAN20 | 0.3221948 | 9.27922E-14 | 1.30929E-12 |
| ZWILCH | 0.3396679 | 3.27645E-15 | 6.05527E-14 |
| ZYG11B | 0.4370234 | 3.72872E-25 | 4.72843E-23 |
| ZZZ3 | 0.5538813 | 2.91078E-42 | 5.33545E-39 |

## Supplementary Table 2. Cancer-related annotations of *PIGK* co-expressed genes identified by IPA

| Diseases or Functions Annotation | *P*-value | Predicted Activation State | Activation z-score | Molecules |
|---|---|---|---|---|
| Cancer | 6.79E-180 | Increased | 2.049 | 1882 |
| Organization of cytoplasm | 3.08E-17 | Increased | 3.661 | 329 |
| Repair of DNA | 1.01E-16 | Increased | 4.367 | 97 |
| Cell death of osteosarcoma cells | 1.56E-13 | Increased | 3.244 | 38 |
| Cell viability | 7.66E-13 | Increased | 3.916 | 310 |
| Cell survival | 2.18E-12 | Increased | 4.135 | 318 |
| Microtubule dynamics | 8.76E-12 | Increased | 3.522 | 253 |
| Organization of cytoskeleton | 1.01E-11 | Increased | 3.635 | 281 |
| Cell proliferation of tumor cell lines | 2.08E-11 | Increased | 4.182 | 435 |
| DNA replication | 6.33E-11 | Increased | 2.212 | 63 |
| Cell viability of tumor cell lines | 9.58E-11 | Increased | 3.974 | 218 |
| Metabolism of DNA | 1.22E-09 | Increased | 2.047 | 82 |
| Formation of cellular protrusions | 3.96E-09 | Increased | 2.965 | 203 |
| Interphase | 1.47E-08 | Increased | 2.244 | 151 |
| Repair of cells | 3.83E-08 | Increased | 3.636 | 45 |
| Double-stranded DNA break repair | 5.61E-08 | Increased | 2.813 | 48 |
| Necrosis of tumor | 9.29E-08 | Increased | 2.126 | 88 |
| Cell death of tumor cells | 1.19E-07 | Increased | 2.126 | 86 |
| Invasive cancer | 1.32E-07 | Increased | 3.576 | 288 |
| Repair of tumor cell lines | 1.47E-07 | Increased | 3.666 | 37 |
| Double-stranded DNA break repair of tumor cell lines | 1.6E-07 | Increased | 2.568 | 27 |
| Double-stranded DNA break repair of cells | 2.77E-07 | Increased | 2.781 | 32 |
| Cell death of fibroblast cell lines | 4.18E-07 | Increased | 2.518 | 78 |

## Supplementary Table 3. PIGK-interacting proteins from BioGRID

| #BioGRID Interaction ID | Official Symbol Interactor A | Official Symbol Interactor B | Experimental System | Organism Name Interactor B |
|---|---|---|---|---|
| 3180044 | B3GAT3 | PIGK | Affinity Capture-MS | Homo sapiens |
| 2638007 | BST2 | PIGK | Affinity Capture-MS | Homo sapiens |
| 2981524 | CALR3 | PIGK | Proximity Label-MS | Homo sapiens |
| 3349740 | CANX | PIGK | Affinity Capture-MS | Homo sapiens |
| 3376722 | CCDC47 | PIGK | Affinity Capture-MS | Homo sapiens |
| 3581082 | CLEC16A | PIGK | Affinity Capture-MS | Homo sapiens |
| 3159099 | CLEC2B | PIGK | Affinity Capture-MS | Homo sapiens |
| 3352167 | COPE | PIGK | Affinity Capture-MS | Homo sapiens |
| 3181172 | CST9L | PIGK | Affinity Capture-MS | Homo sapiens |
| 2798069 | E | PIGK | Proximity Label-MS | Homo sapiens |
| 2887886 | E5 | PIGK | Affinity Capture-MS | Homo sapiens |
| 2782375 | EMC1 | PIGK | Affinity Capture-MS | Homo sapiens |
| 2804000 | ESR1 | PIGK | Affinity Capture-MS | Homo sapiens |
| 2520291 | ESR2 | PIGK | Affinity Capture-MS | Homo sapiens |
| 2935761 | FAM20C | PIGK | Affinity Capture-MS | Homo sapiens |
| 3171217 | FBXO2 | PIGK | Affinity Capture-MS | Homo sapiens |
| 912908 | FBXO6 | PIGK | Affinity Capture-MS | Homo sapiens |
| 3148831 | GP9 | PIGK | Affinity Capture-MS | Homo sapiens |
| 286659 | GPAA1 | PIGK | Affinity Capture-Western | Homo sapiens |
| 3624752 | GRM2 | PIGK | Affinity Capture-MS | Homo sapiens |
| 3517642 | HIST1H3C | PIGK | Proximity Label-MS | Homo sapiens |
| 3148134 | HS2ST1 | PIGK | Affinity Capture-MS | Homo sapiens |
| 3173155 | IDS | PIGK | Affinity Capture-MS | Homo sapiens |
| 2831861 | KIF20A | PIGK | Affinity Capture-MS | Homo sapiens |
| 3193231 | KLK15 | PIGK | Affinity Capture-MS | Homo sapiens |
| 3162195 | LPAR1 | PIGK | Affinity Capture-MS | Homo sapiens |
| 2995093 | LRRC59 | PIGK | Proximity Label-MS | Homo sapiens |
| 1189086 | LYPD3 | PIGK | Affinity Capture-MS | Homo sapiens |
| 2933123 | NR3C1 | PIGK | Proximity Label-MS | Homo sapiens |
| 2800159 | nsp4 | PIGK | Proximity Label-MS | Homo sapiens |
| 2876161 | nsp4ab | PIGK | Affinity Capture-MS | Homo sapiens |
| 2800561 | nsp6 | PIGK | Proximity Label-MS | Homo sapiens |
| 3495571 | ORF10 | PIGK | Proximity Label-MS | Homo sapiens |
| 2801287 | ORF14 | PIGK | Proximity Label-MS | Homo sapiens |
| 2802155 | ORF7a | PIGK | Proximity Label-MS | Homo sapiens |
| 2802629 | ORF7b | PIGK | Proximity Label-MS | Homo sapiens |
| 3580506 | ORF8 | PIGK | Proximity Label-MS | Homo sapiens |
| 3165396 | P2RX2 | PIGK | Affinity Capture-MS | Homo sapiens |
| 3402867 | PARK2 | PIGK | Affinity Capture-MS | Homo sapiens |
| 2999388 | PDIA4 | PIGK | Proximity Label-MS | Homo sapiens |
| 3362777 | PGRMC1 | PIGK | Affinity Capture-MS | Homo sapiens |
| 1450065 | PIGS | PIGK | Affinity Capture-MS | Homo sapiens |
| 3177773 | PIGT | PIGK | Affinity Capture-MS | Homo sapiens |
| 3396818 | PLD3 | PIGK | Affinity Capture-MS | Homo sapiens |
| 3396925 | PLD4 | PIGK | Affinity Capture-MS | Homo sapiens |
| 3396998 | PLD6 | PIGK | Affinity Capture-MS | Homo sapiens |
| 2749571 | PLEKHA4 | PIGK | Affinity Capture-MS | Homo sapiens |
| 3486678 | POMK | PIGK | Affinity Capture-MS | Homo sapiens |
| 3167930 | PRG2 | PIGK | Affinity Capture-MS | Homo sapiens |

| | | | | |
|---|---|---|---|---|
| 1190099 | PRSS50 | PIGK | Affinity Capture-MS | Homo sapiens |
| 3181011 | PSCA | PIGK | Affinity Capture-MS | Homo sapiens |
| 3379303 | RAB7A | PIGK | Proximity Label-MS | Homo sapiens |
| 3517814 | RAF1 | PIGK | Affinity Capture-MS | Homo sapiens |
| 3331714 | RHOA | PIGK | Affinity Capture-MS | Homo sapiens |
| 3335602 | RHOF | PIGK | Affinity Capture-MS | Homo sapiens |
| 2492930 | RNF4 | PIGK | Affinity Capture-MS | Homo sapiens |
| 3630456 | RPA1 | PIGK | Proximity Label-MS | Homo sapiens |
| 3633488 | RPA2 | PIGK | Proximity Label-MS | Homo sapiens |
| 3635861 | RPA3 | PIGK | Proximity Label-MS | Homo sapiens |
| 3004896 | RPN1 | PIGK | Proximity Label-MS | Homo sapiens |
| 2637122 | SLC15A3 | PIGK | Affinity Capture-MS | Homo sapiens |
| 3162906 | ST8SIA5 | PIGK | Affinity Capture-MS | Homo sapiens |
| 3008711 | SYNE3 | PIGK | Proximity Label-MS | Homo sapiens |
| 1430907 | TCTN3 | PIGK | Proximity Label-MS | Homo sapiens |
| 3563984 | TGOLN2 | PIGK | Proximity Label-MS | Homo sapiens |
| 3091860 | THY1 | PIGK | Affinity Capture-MS | Homo sapiens |
| 3373319 | TMED10 | PIGK | Affinity Capture-MS | Homo sapiens |
| 3186940 | TMEM106A | PIGK | Affinity Capture-MS | Homo sapiens |
| 3306216 | TMEM106B | PIGK | Proximity Label-MS | Homo sapiens |
| 2689982 | TRIM28 | PIGK | Affinity Capture-MS | Homo sapiens |
| 3522049 | TRIM67 | PIGK | Affinity Capture-MS | Homo sapiens |
| 3155209 | TSPAN15 | PIGK | Affinity Capture-MS | Homo sapiens |
| 2637564 | UNC93B1 | PIGK | Affinity Capture-MS | Homo sapiens |
| 3374737 | VAPA | PIGK | Affinity Capture-MS | Homo sapiens |
| 3148953 | VAPB | PIGK | Affinity Capture-MS | Homo sapiens |
| 3472271 | ZRANB1 | PIGK | Affinity Capture-MS | Homo sapiens |

**Supplementary Table 4. PIGK-interacting proteins significantly correlated with *PIGK* expression in TCGA/HNC**

| Official Symbol Interactor A | Correlation significance | Correlation coefficient |
|---|---|---|
| PIGS | *** | 0.508 |
| IDS | *** | 0.327 |
| EMC1 | *** | 0.662 |
| CCDC47 | *** | 0.414 |
| B3GAT3 | * | 0.108 |
| NR3C1 | *** | 0.59 |
| CLEC16A | *** | 0.403 |
| PGRMC1 | *** | 0.462 |
| CANX | *** | 0.615 |
| CALR3 | *** | 0.173 |
| HS2ST1 | *** | 0.71 |
| LYPD3 | *** | -0.279 |
| LRRC59 | *** | 0.37 |
| FAM20C | *** | 0.217 |
| PDIA4 | *** | 0.463 |
| PARK2 | *** | 0.31 |
| HIST1H3C | * | 0.101 |
| GRM2 | ** | 0.346 |
| GPAA1 | ** | 0.249 |
| ESR1 | *** | 0.333 |
| LPAR1 | *** | 0.454 |
| KIF20A | *** | 0.54 |
| ESR2 | *** | 0.237 |
| PIGT | *** | 0.464 |
| PLD3 | *** | 0.353 |
| PLD4 | *** | 0.252 |
| PLD6 | *** | 0.207 |
| PLEKHA4 | *** | 0.231 |
| POMK | *** | 0.451 |
| PRG2 | ** | 0.146 |
| PRSS50 | * | 0.109 |
| PSCA | * | -0.115 |
| RAB7A | *** | 0.243 |
| RAF1 | *** | 0.422 |
| RHOA | *** | 0.446 |
| RNF4 | *** | 0.481 |
| RPA1 | *** | 0.494 |
| RPA2 | *** | 0.469 |
| RPA3 | *** | 0.302 |
| RPN1 | *** | 0.44 |
| SLC15A3 | *** | 0.164 |
| ST8SIA5 | *** | 0.198 |
| SYNE3 | *** | 0.433 |
| TCTN3 | *** | 0.528 |
| TGOLN2 | *** | 59 |
| THY1 | *** | 0.292 |
| TMED10 | *** | 0.6 |
| TMEM106A | *** | 0.505 |
| TMEM106B | *** | 0.5 |

| | | |
|---|---|---|
| TRIM28 | *** | 0.179 |
| TRIM67 | *** | 0.28 |
| TSPAN15 | *** | 0.289 |
| UNC93B1 | *** | 0.296 |
| VAPA | *** | 0.37 |
| VAPB | *** | 0.475 |
| ZRANB1 | *** | 0.45 |

*$P < 0.05$, **$P < 0.01$, ***$P < 0.001$

**Supplementary Table 5. Refined physical interactors of PIGK used for PPI network construction**

| Gene |
|---|
| B3GAT3 |
| CALR3 |
| CANX |
| CCDC47 |
| EMC1 |
| ESR1 |
| ESR2 |
| FAM20C |
| GPAA1 |
| HS2ST1 |
| KIF20A |
| LPAR1 |
| LRRC59 |
| LYPD3 |
| NR3C1 |
| PDIA4 |
| PGRMC1 |
| PIGS |
| PIGT |
| PLD3 |
| PLD4 |
| PLEKHA4 |
| PRSS50 |
| RAB7A |
| RAF1 |
| RHOA |
| RNF4 |
| RPA1 |
| RPA2 |
| RPA3 |
| RPN1 |
| TCTN3 |
| TGOLN2 |
| THY1 |
| TMED10 |
| TMEM106B |
| TRIM28 |
| UNC93B1 |
| VAPA |
| VAPB |
